# LipidAnalyst: A Comprehensive Tool for Lipidomic Data Visualization and Analysis

**DOI:** 10.3390/metabo16080526

**Published:** 2026-07-25

**Authors:** Xinyi Liu, Alla Karnovsky, Subramaniam Pennathur, Farsad Afshinnia

**Affiliations:** 1 Department of Computational Medicine and Bioinformatics, University of Michigan, Ann Arbor, MI 48109, USA; lxylily@umich.edu (X.L.); akarnovs@med.umich.edu (A.K.); 2 Department of Internal Medicine, Division of Nephrology, University of Michigan, Ann Arbor, MI 48109, USA; 3 Department of Molecular and Integrative Physiology, University of Michigan, Ann Arbor, MI 48109, USA

**Keywords:** lipidomics, data processing, visualization, analysis, software

## Abstract

**Introduction:** Proper analysis of high-throughput lipidomic data requires specialized tools for data processing, normalization, visualization, and statistical and bioinformatic analysis. However, limitations in lipid parsing, data processing, and visualization capabilities in existing software packages create challenges for comprehensive lipidomic data analysis. To address these limitations, we developed LipidAnalyst (v 1.0.3), a user-friendly tool designed to facilitate efficient lipid parsing and processing, visualization, and analysis of lipidomic datasets. **Methods:** LipidAnalyst was developed using the R Shiny framework. It is hosted on MiServer for online work but can also be downloaded from GitHub. **Results:** LipidAnalyst provides functionalities in three major areas: data processing, visualization, and statistical analysis. Data processing features include quality control filtering, normalization, internal standard-based quantification, and unique capabilities for missing-value imputation, lipid parsing, and aggregation. Visualization tools include box and violin plots for data distribution assessment, principal component analysis (PCA) plots, hierarchical clustering and differential abundance heatmaps, volcano plots, correlation plots, and Debiased Sparse Partial Correlation (DSPC) clustering plots. Statistical analysis modules include *t*-test, analysis of variance (ANOVA), Partial Least Squares Differential Analysis (PLS-DA), Orthogonal Partial Least Squares Differential Analysis (OPLS-DA), and Random Forest (RF) modeling. **Conclusions:** LipidAnalyst is a comprehensive platform for optimal processing, visualization, and analysis of lipidomic data. By integrating advanced data processing workflows with extensive visualization and statistical analysis capabilities, LipidAnalyst enables researchers to explore lipidomic datasets more effectively and develop informed analytical strategies.

## 1. Introduction

Historically, clinical lipid research has been limited to the measurement of total cholesterol, serum lipoproteins, and total triglyceride in circulation. However, lipids are the most diverse metabolites in circulation [1,2], with various species and diverse roles in different aspects of metabolism and cell physiology. There are over thousands of different lipids that can be classified into major lipid classes beyond the lipid panels measured in clinical practice [3,4]. Technological advances in mass spectrometry provide opportunities to explore the lipidome in biological samples with high granularity and to investigate its relevance to disease in various studies [5,6,7,8,9,10,11]. Both targeted and untargeted lipidomic platforms can rapidly measure and identify thousands of lipid species, generating large and complex datasets for clinical studies [12,13]. Proper analysis of such data, particularly from untargeted lipidomic platforms, presents significant challenges. These challenges are compounded when data-driven analytical approaches are applied to datasets in which the number of measured lipid species exceeds the sample size. Consequently, careful data processing and the development of an appropriate analytical strategy are essential for obtaining reliable and biologically meaningful results.

Historically, the study of the differential phenotypic expression of various metabolites in large datasets has been primarily focused on the identification of the top differentially expressed metabolites, irrespective of the relevance of the remaining metabolites to differential system biology [14,15,16]. This approach heavily relies on univariate compound-by-compound analysis, often using volcano plots to visualize the results. A fundamental assumption for the application of a compound-by-compound analysis is that the variability of the candidate metabolite is almost entirely explained by the phenotype of interest and its severity. Univariate approaches work well for biomarkers whose variability is dominated by phenotype (e.g., troponin in myocardial injury [17,18]); they are less appropriate for heterogeneous chronic diseases such as chronic kidney disease. Furthermore, the application of system biology approaches unravels the existence of ongoing meaningful differential biology by disease state that may not simply be limited to the top differentially identified candidates proposed by a compound-by-compound approach [17,18]. There is a need for data analysis platforms that provide both system-level and compound-level views of lipidomic data, incorporating lipid structural features and classification by class, carbon chain length, and saturation, to facilitate the discovery of disease-associated metabolic pathways.

Alterations in lipidomic profiles reflect the net effect of multiple biological processes, including dietary factors, de novo lipogenesis, lipolysis, elongation, and desaturation [19]. These processes may be differentially affected by disease states, resulting in changes in the abundance and composition of lipids within and across lipid classes. Consequently, data visualization becomes an integral part of data analysis, as it can help understand the structure of the data and guide the selection of appropriate data analysis techniques. Although a number of excellent online tools (MetaboAnalyst [20], LipidSig [21], LipidOne [22], LipidSuite [23], LipidSigR [24], and Lipidr [25]) can help guide data, there are still gaps in lipid parsing, data processing, data normalization, and visualization. First, many existing software tools have limitations in lipid parsing because they rely heavily on database-dependent name matching or require lipids to follow strict nomenclature conventions. As a result, their flexibility in handling diverse or inconsistently annotated lipid names is reduced. Second, while most software provides general data processing and normalization methods, they often lack methods tailored to lipidomic datasets, such as merging lipid duplicates or adducts and internal standard normalization. Because lipid expression levels may vary substantially across different lipid classes, class-level normalization would be beneficial in removing the systematic difference between lipid classes. However, these lipid-specific normalization utilities remain limited in currently available software tools. Third, systematic interpretation of lipidomic data often requires the analysis of lipid structural features rather than focusing on individual lipid species. Visualization tools that incorporate structural information, such as lipid saturation status and acyl chain length distributions, can facilitate the identification of biologically meaningful patterns and support hypothesis generation from lipidomic datasets. In response to these gaps, we have designed “LipidAnalyst”, a user-friendly R-based interactive data management program for optimized normalization and visualization of lipidomic data, which is suitable for users who are not comfortable with command line tools, allowing the choice of proper analytical plan.

## 2. Methods

### 2.1. LipidAnalyst Overview

**Figure 1:**
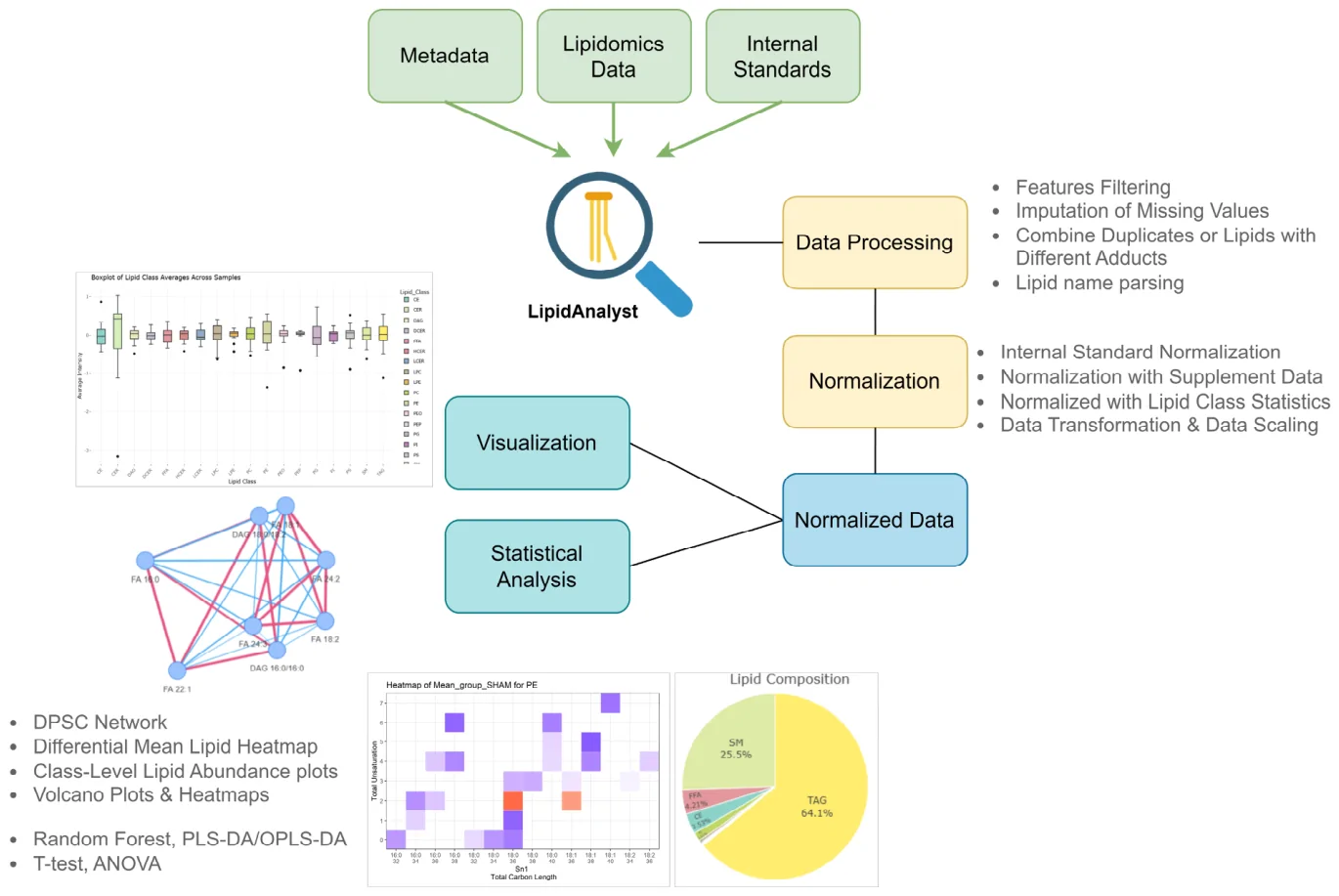
Overview of LipidAnalyst (icon created with BioRender.com).

LipidAnalyst was developed in R (v 4.4.3) using shiny(v 1.14.0). It is hosted on MiServer. As shown in Figure 1, LipidAnalyst accepts three types of input files: lipidomic data tables, metadata to specify the sample grouping information, and an optional file containing internal standard information that can be used for data normalization (see Example Data File in the software for sample file format). The first step in the LipidAnalyst pipeline is data processing. It includes feature filtering, removal and imputation of missing values, and optional grouping of different isomers into singular features. The second step is data normalization, which includes internal standard normalization, normalization using user-supplied metadata, sample normalization, data transformation, and scaling. The normalized data can then be used in visualization and statistical analysis.

### 2.2. Input Data, Quality Control, and Data Normalization

**Data processing:** Data processing in LipidAnalyst includes data filtering, imputation, and feature aggregation of both processed and annotated lipids.

Data filtering helps to eliminate noise and irrelevant features, ensuring that subsequent analyses focus on meaningful biological variations. Users can apply filters to remove low-quality, low-abundance, and low-variance features. The low-quality filter removes features with a high percentage of missing values. We recommend 50% as a default threshold, i.e., features with missing values in more than half of the samples will be filtered out. The low-abundance filter removes features with a mean lower than a specified percentile across all features. Since lipid abundances can vary by several orders of magnitude, applying this filter could remove low-abundance lipids, leading to the loss of meaningful biological information. The low-variance filter removes features with low variability across samples, which would not be useful for distinguishing between different conditions. In large datasets, this filtering step can reduce the number of hypotheses tested, thereby improving statistical power after multiple-testing correction. However, because many lipidomic datasets contain a moderate number of features, the benefit is often limited, and we therefore consider this step optional rather than a recommended default.

We categorize missingness into two major types: (1) Group-level missingness: A lipid is almost completely missing within one experimental group (e.g., all disease group samples have NA). This often suggests true biological absence or very low abundance. In these cases, we recommend imputing group-level missing values using one-fifth of the minimum observed value, as lipid features missing in ≥95% of samples within a group are generally considered to be below the limit of detection. (2) General missingness: Values are sporadically missing across samples but not confined to a single group. This pattern typically reflects technical noise or stochastic signal dropout. For this scenario, K-Nearest Neighbors (KNN) imputation (sample-wise) is recommended, as it leverages similarity among samples to estimate reasonable values. We also encourage users to examine the missing-value heatmap to assess the likely causes of missingness.

In lipidomic data, it is common to see different chemical adducts for the same lipid species (e.g., [M+H]+, [M+Na]+, [M+K]+). Combining these adducts into a single representative entry can help streamline data analysis and interpretation. We recommend combining lipid features to deal with duplicated lipids or lipids with different adducts. Users can select different methods to combine lipids such as using the mean or sum of the duplicated lipids. When aggregation of different lipid isoforms into a single lipid feature is desired, summing duplicate features is recommended. Whereas, when the duplicate features represent repeated measurements of the same lipid species, we recommend averaging their values to obtain a single representative measurement.

**Lipid parsing:** The lipid parsing function ensures that lipid names are correctly interpreted and helps identify the lipid class and lipid chains. Lipid parsing in LipidAnalyst implements a hybrid dictionary- and rule-based framework. General lipid names lacking explicit structural annotations are first standardized into shorthand nomenclature through lookup-table matching against the LIPID MAPS^®^ Structure Database (LMSD) [26,27,28]. Standardized lipid names are subsequently processed using class-specific regular expression rules to extract lipid class, fatty acyl composition, total carbon number, and double-bond content.

Lipid class information is extracted from the prefix of each lipid name and further harmonized using predefined class-mapping rules, such as converting TAG, DAG, MAG, and FFA to TG, DG, MG, and FA, respectively. Ether-linked lipids containing O- or P- prefixes were identified separately and annotated as lipid-class-specific subclasses.

Fatty acyl chain information is extracted using regular expression patterns that capture carbon number and double-bond annotations in the format “number”, such as 18:1 or 20:4. For lipids containing multiple chain annotations, individual chains are parsed separately.

Additional class-specific parsing rules are implemented. For sphingolipids, including SM, Cer, HexCer, LacCer, and dhCer, when only one chain annotation is available for these lipid classes, the parser infers the sphingoid backbone based on class-specific assumptions and calculates the remaining fatty acyl chain accordingly. Specifically, if only one chain is reported in the sphingolipid, and that chain has a reported carbon number of more than 30, then LipidAnalyst assumes the sum notation is adopted.

Given that TG lipids are expected to contain three acyl chains, and DG, PC, PE, PE(O), PE(P), PI, and PS lipids are expected to contain two acyl chains, when LipidAnalyst encounters annotations reporting only a single value for the carbon number of these lipid classes, the reported carbon number is interpreted as the sum of the carbon numbers from all acyl chains as the composition nomenclature.

Furthermore, the lipid parsing results generated by LipidAnalyst are fully editable, enabling users to review and modify annotations when inconsistencies with their prior knowledge or dataset-specific conventions are identified.

**Normalization:** Normalization is a crucial step to remove technical differences and data skewness for lipidomic data analysis. We present internal standard normalization, normalization by supplementing data such as cell counts or dilution factors, sample-wise normalization, lipid class-aware normalization, data transformation methods, and data scaling methods.

Internal standard normalization is performed by normalizing lipid expression levels to their corresponding internal standards. We recommend quantifying lipids using their corresponding internal standards whenever available. If no matching internal standard is included in the experiment, then an internal standard with a similar lipid class may be selected.

Mean normalization can be used before unsupervised exploration like PCA or OPLS-DA. It is not recommended for primary sample-level scaling in lipidomics because lipid abundance is usually highly skewed and sensitive to extreme values.

Median normalization can be considered for large-scale untargeted datasets with systematic instrument variations or when working with samples where total lipid abundance varies significantly, or upon inconsistencies in sample matrices or volumes. It is resilient against distortion by extreme data and is therefore ideal for studies where there are many lipid species and the bulk of the lipids are expected to be unaffected by experimental conditions. It may cause artifacts if a large, highly abundant lipid class such as triglycerides or structural phospholipids naturally fluctuates between control and treatment groups.

Class sum normalization is recommended when biological interpretation requires observing structural shifts or relative proportions inside a specific lipid class commensurate with differential elongation and desaturation. Because this pipeline relies heavily on ratios and relative proportions rather than absolute levels, it may obscure total lipid mass changes across different samples or treatment groups. So, if the biological question hinges on observing a global knockdown/increase in total lipid levels or expecting up- or down-regulation of inter-class lipid converting pathways, then sample-wise sum normalization is likely more suitable than lipid class sum normalization. Whereas, when intra-class alterations of elongation or desaturation are suspected, class sum normalization is preferred.

Quantile normalization is appropriate in lipidomic analysis when there is a need to align global abundance distributions across multiple samples to remove technical batch effects or injection-order biases, provided the underlying distribution of lipid species remains largely unchanged between the experimental conditions, ensuring that sample-to-sample variation is driven by biological variation rather than technical drift. It is well-suited for untargeted lipidomic workflows where sample intensity distributions are heavily skewed by extreme values. This method assumes that the global lipid profile does not change between groups. So, if a massive biological shift is expected (e.g., a specific treatment causing an extreme lipid accumulation or depletion), then this method may artificially wipe out the biological differences in lipids intended to be measured.

Log transformation is appropriate when the lipidomic data are right-skewed and exhibit heteroscedasticity. It stabilizes variance across the dynamic range, converts multiplicative changes into additive ones, and makes data more normally distributed for parametric statistical tests.

For sum-normalized data (ranging between 0 and 1), logit transformation is highly recommended as it handles proportional data much more effectively than standard logarithmic transformations, which can skew ratios. It stabilizes the variance and establishes normally distributed data without boundaries (from −ꝏ to +ꝏ).

Square root transformation is appropriate when dealing with severely right-skewed, heteroscedastic lipidomic datasets where global sample distributions must be strictly aligned and variance needs to be stabilized without artificially inducing false positives. It forces all samples to share the same empirical distribution. It performs best when the dataset contains a large number of detected lipid features (typically >1000). When the research aim is detecting differences in statistical distributions (rather than absolute fold-change magnitude) across different sample cohorts, quantile transformation can provide increased testing power.

Cube root transformation is appropriate for lipidomic analyses when dealing with heavily right-skewed data that contain numerous zero, near-zero, or negative intensity values. It stabilizes variance across the abundance spectrum while making samples directly comparable for downstream univariate or multivariate statistical modeling. It has the potential to artificially suppress true biological variation if global lipid abundance genuinely differs between the experimental groups.

Auto-scaling is appropriate when the study priority is treating all lipid species equally, regardless of their natural abundance, and when preparing data for variance-sensitive multivariate models like Principal Component Analysis (PCA) or Partial Least Squares Discriminant Analysis (PLS-DA). This prevents masking alterations of low-abundance lipids such as long-chain acylcarnitines by high-abundance lipids in the same class such as L-carnitine. It is well-suited as an input step for downstream classification algorithms like Support Vector Machines (SVMs) and Artificial Neural Networks (ANNs), which rely heavily on feature correlation structures rather than raw concentration magnitudes. It allows interpreting data in terms of “fold standard deviations”—specifically, how many standard deviations a lipid is above or below the study average. It may cause noise amplification for low-abundance lipids. Hence, when the aim is to identify subtle, biologically relevant changes in lower-abundance lipids, pareto scaling can be considered. It reduces the influence of large fold changes without inflating baseline noise.

Range scaling is appropriate in lipidomic analysis for multivariate exploratory analysis like PCA, dose–response modeling, and comparing lipid species with vastly different baseline concentrations. Where there are diverse lipids from highly abundant species to trace lipids, range scaling gives equal weight to all lipids, preventing the highly abundant lipids from skewing statistical models. It is sensitive to extreme outliers and may inflate noise in studies with low sample size.

### 2.3. LipidAnalyst Analysis Methods

**Univariate statistical analysis:** LipidAnalyst provides univariate statistical testing to identify individual lipid species or lipid classes that differ significantly between experimental groups. Supported methods include Student’s *t*-test and Welch’s *t*-test to identify differential lipids in two groups, and analysis of variance (ANOVA) to identify differential lipids in more than two groups, depending on study design and variance assumptions. Fold change analysis and volcano plots are implemented to jointly visualize effect size and statistical significance, enabling rapid identification of biologically meaningful lipid alterations. Differential Mean Lipid Class Heatmaps enable hypothesis-driven exploration of disease-associated lipid alterations by mapping mean abundance differences across lipid carbon chain length and saturation states.

**Multivariate projection and dimension reduction:** To capture global lipidomic patterns and assess sample-level structure, LipidAnalyst implements multivariate projection and dimension reduction techniques including principal component analysis (PCA), an unsupervised method recommended for exploratory analysis to visualize sample clustering and detect outliers; Partial Least Squares Discriminant Analysis (PLS-DA) and Orthogonal PLS-DA (OPLS-DA), supervised methods designed to maximize separation between groups and identify discriminatory lipids; and RF, a supervised ensemble learning method used to rank lipid features according to their contribution to group discrimination (Supplement Methods). These methods facilitate visualization of group separation, detection of outliers, and identification of lipids contributing most strongly to phenotypic differences. For PLS-DA and OPLS-DA, cross-validation and permutation tests are implemented to estimate predictive performance on unseen data and reduce the risk of overfitting. For RF, LipidAnalyst relies on the inherent bootstrap aggregation (bagging) procedure of the algorithm and provides multiple performance metrics, including accuracy, accuracy *p*-value, Cohen’s kappa, and McNemar’s test *p*-value, to assist users in evaluating model reliability. All analyses are deterministic and reproducible. Hierarchical clustering and heatmaps are provided for both lipid- and sample-centric exploratory analysis. For algorithms with stochastic components, a fixed random seed is used to ensure reproducible outputs.

**Network and dependency analysis:** Beyond abundance-based analyses, LipidAnalyst supports network-based approaches to investigate lipid–lipid dependency structures. Pearson Correlation networks and Differential Sparse Partial Correlation (DSPC) networks [29] are implemented to identify coordinated lipid regulation and condition-specific network rewiring. DSPC is intended for exploratory network analysis by building sparse partial correlation networks that prioritize lipid–lipid associations. A fixed random seed is used to ensure reproducible outputs. These analyses enable the detection of altered lipid interaction patterns that may not be apparent from univariate statistics alone.

**Software availability:** All software development was conducted in R (v 4.4.3). The computational environment, including R and all package dependencies, is encapsulated within a Docker image to ensure reproducibility across systems. LipidAnalyst provides a unified interface that is containerized using Docker (v 25.0.3) and deployed on the MiServer using Podman (v 5.8.2). It is numbered LipidAnalyst v 1.0.3 and is made publicly available on GitHub (https://github.com/LilyLiuX/LipidAnalyst (accessed on 22 July 2026)) and archived on Zenodo (10.5281/zenodo.21326421). LipidAnalyst is distributed under the MIT License. It is currently available at (https://lipidanalyst.miserver.it.umich.edu/lipidanalyst/ (accessed on 22 July 2026)).

**Hardware requirements:** No specific operating system requirements are imposed on users, as LipidAnalyst is accessed through a web browser. The platform can be accessed through any modern web browser (e.g., Chrome, Firefox, Edge, or Safari) on Windows, macOS, or Linux operating systems. However, users who wish to deploy LipidAnalyst locally using the provided container image will require a system capable of running Docker or Podman. We recommend a minimum of 2 CPU cores and 4 GB RAM for routine analyses. The public web instance is currently hosted on a Linux server with 2 CPU cores and 4 GB RAM. To download the software for local deployment, follow the downloading instructions (Supplements: Downloading instructions from GitHub).

## 3. Results

Working with LipidAnalyst starts with the welcome page (Figure S1). Users can upload their own lipidomic data or load the example lipidomic dataset [30] (Figure S2) and visualize the data (Figure S3). Next, users can upload the metadata (Figure S4) and the file containing internal standards (optional) (Figure S5). Subsequently, users have the option to filter the data by setting the missing, abundance, or variance thresholds, or simply skip data filtering (Figure S6). Next, the extent of missingness can be assessed (Figure S8) and imputed using various imputation techniques (Figures S7 and S9). Some users may prefer to combine various isoforms of a certain lipid into one entry. For example, TG48:0 represented three isoforms with different acyl chains at Sn1 carbon including TAG48:0-FA14:0, TAG48:0-FA16:0, and TAG48:0-FA18:0 in the example lipidomic data. To do so, users can choose the desired lipid class in which their members need to be combined along with the method of combining the corresponding isoforms into a single value, or skip this step (Figure S10). In the next step, users can verify class level of each lipid, carbon numbers, and number of double bonds (unsaturation) at Sn1 to Sn3 carbons using Lipid Parsing Controls (Figures S11 and S12). Data Preview allows a global preview of data distribution (Figure S13) before digging into downstream processing. If internal standards are provided, then users then can choose the appropriate internal standards for normalization of various lipid classes in the next step (Figures S14 and S15). Further normalization by a user-defined constant value to account for dilution, weight, or volume factors can be performed or skipped in the next step (Figure S16A,B). Values can also be normalized by the sample-specific metadata factors, such as cell count or protein concentration, if the user provided them in the metadata they uploaded (Figure S16A,B). Next, users may further normalize the data by various statistics (sum, mean, median), transform the data distribution, and apply various scaling methods, or skip any one of the normalization methods (Figure S17).

**Figure 2:**
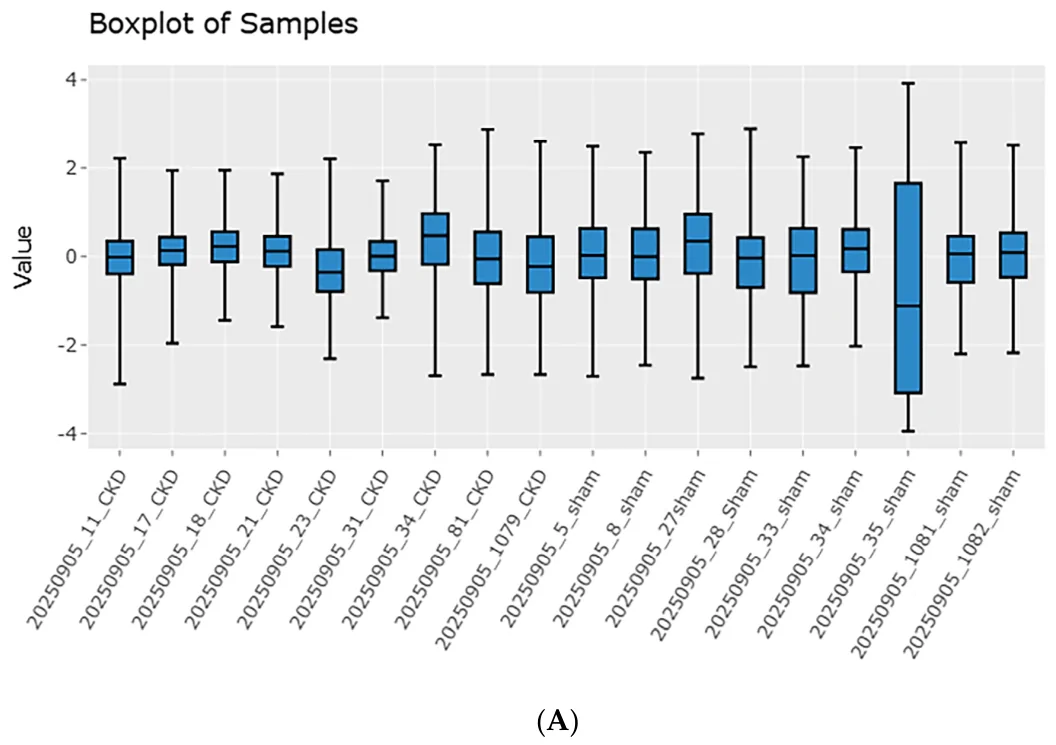

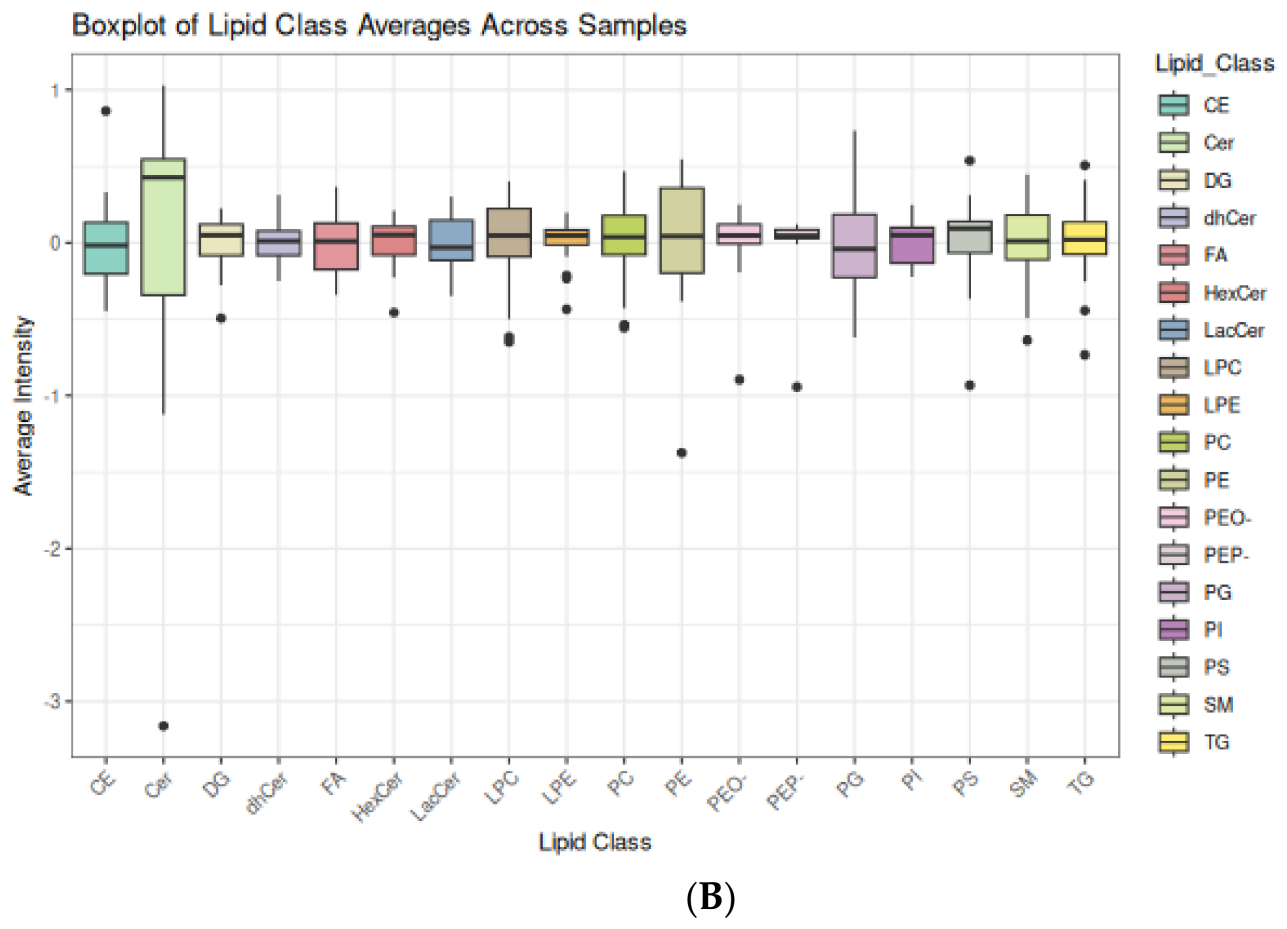
(**A**) Overview of “Global Distribution Boxplot”. boxplots for samples show the sample-based lipidomic distribution of data across study samples. (**B**) Boxplots for lipid class show the distribution of lipids within each lipid class across various classes of lipids.

**Plots:** Visualization begins with “Global Distribution Boxplots” (Figure S18) for samples (Figure 2A), lipids classes (Figure 2B), and single lipids.

The PCA plot provides graphical distribution of samples by study group using the lipidomic data in two-dimensional (Figure S19A) or three-dimensional (Figure S19B) view.

Heatmaps and hierarchical clustering generate hierarchical clustering heatmaps of lipidomic data across study samples (Figure S20).

**Figure 3:**
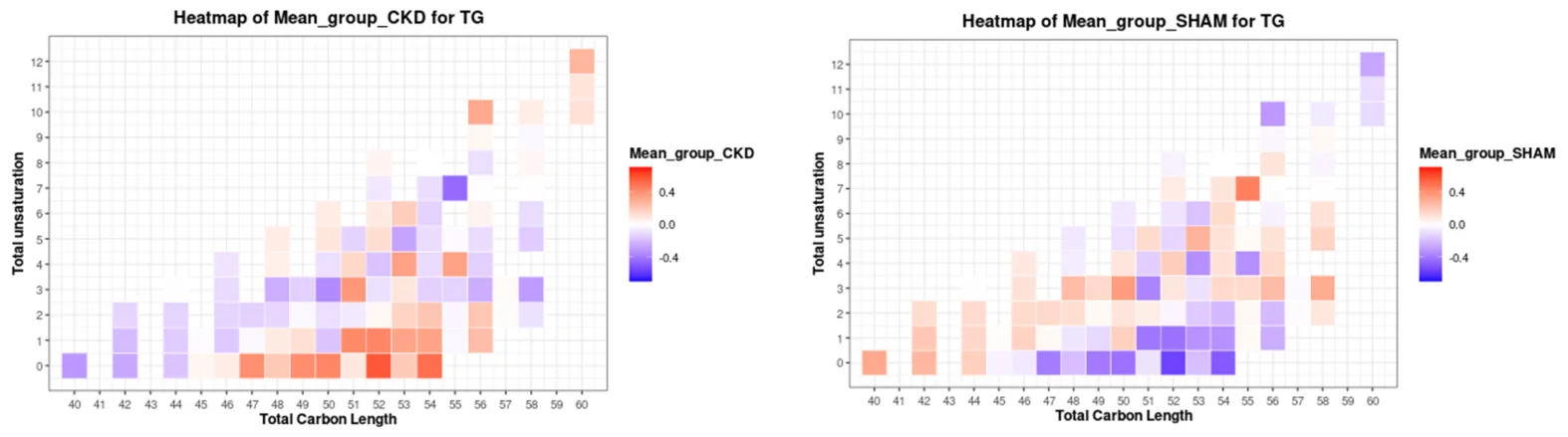
Differential Mean Lipid Heatmap of triacylglycerols (TGs) compares the mean of each lipid by study group. X-axis represents the total number of carbons in three acyl chains, and Y-axis represents the total number of double bonds in three acyl chains.

Differential Mean Lipid Heatmap (DMLH) is introduced to visualize the mean values of various lipids within each lipid class distributed by the number of acyl chain carbons in the X-axis and the number of acyl chain double bonds in Y-axis across study groups (Figure S21). For lipids with a single acyl chain or those with more than one acyl chain but combined by their isoforms into a single value, the X-axis represents the total number of carbons in all chains; for example, in triacylglycerols (TGs, Figure 3).

**Figure 4:**
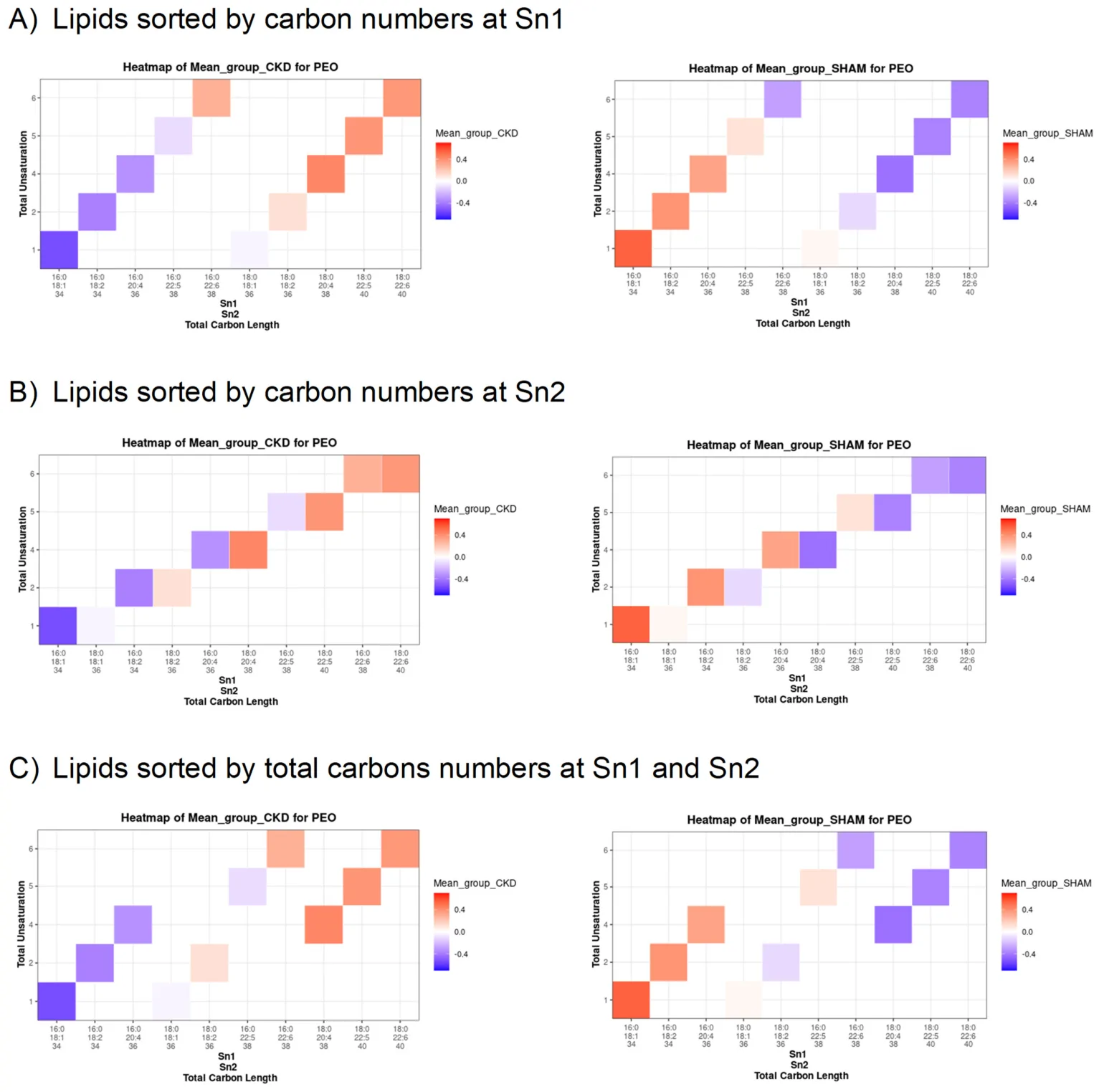
Differential Mean Lipid Heatmap of phosphatidylethanolamine-O (PE-O) compares the mean lipid of each lipid by study group. X-axis represents the number of carbons in acyl chain at Sn1 carbon, number of carbons in acyl chain at Sn2 carbon, or the total number of carbons in acyl chains at Sn1 and Sn2. Y-axis represents the total number of double bonds in the two acyl chains. Users have the flexibility to sort the lipids by carbon number at Sn1 (**A**), Sn2 (**B**), or by total carbon number (**C**) for optimal view.

In lipids with two chains such as diacylglycerols, phosphatidylethanolamines, and phosphatidylcholines, the X-axis can be sorted by the number of carbons at the Sn1 acyl chain, Sn2, or total carbon numbers (sum of Sn1 and Sn2 acyl chain carbon numbers) for optimal visualization (Figure 4A–C).

**Figure 5:**
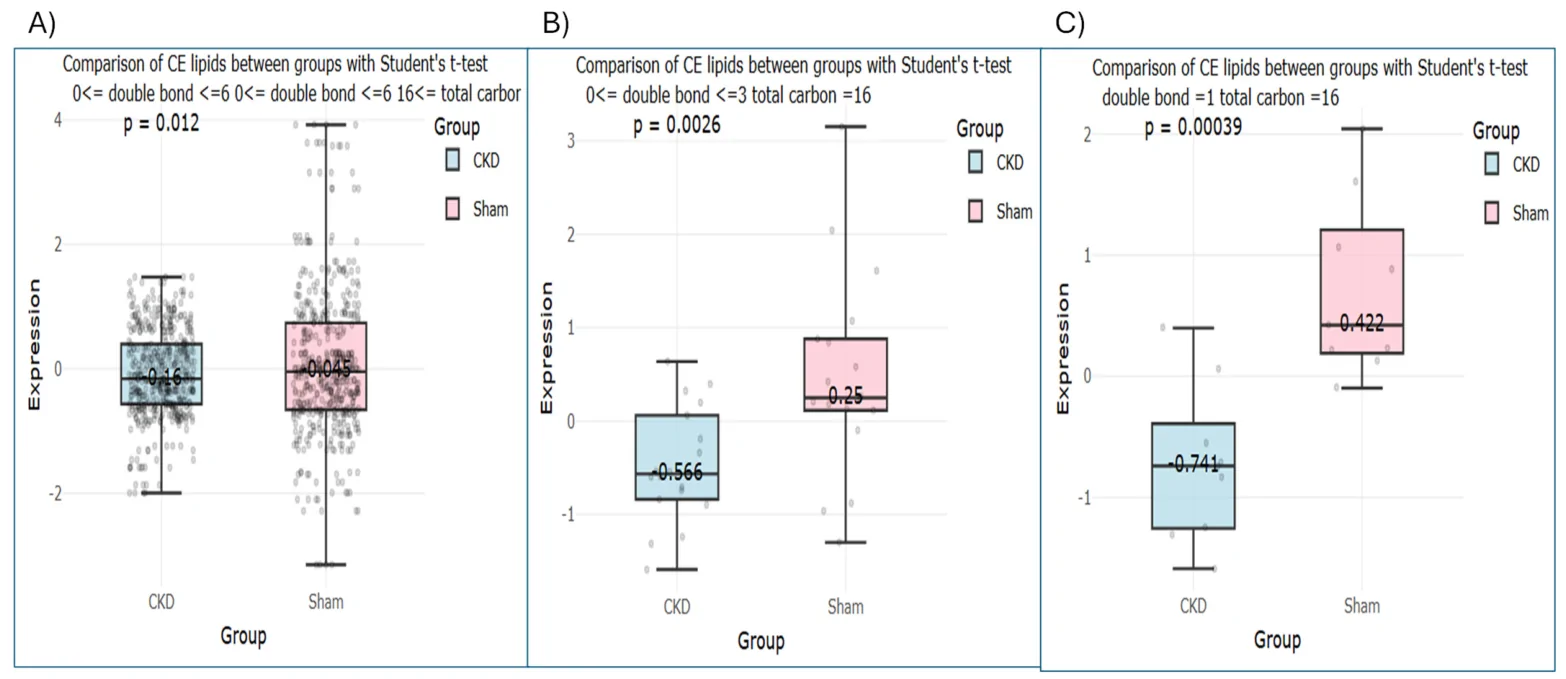
Class-level lipid comparison: Using filters to change the number of carbons or the number of double bonds, the boxplots can represent the distribution of all lipids within each lipid class (**A**), combination of a select number of lipids within each lipid class (**B**), or a single lipid by study group (**C**).

Class-level lipid comparison: Using boxplots, users can compare the distribution of lipids by study group (Figure S22). Using filters to change the number of carbons or the number of double bonds, the boxplots can represent the distribution of all lipids within each lipid class (Figure 5A), the combination of a select number of lipids within each lipid class (Figure 5B), or a single lipid by study group (Figure 5C). Alternatively, violin plots can be used to visualize the data. Box or violin plots of individual lipids by study group can also be viewed in the Individual Lipid Comparison section (Figure S23).

**Figure 6:**
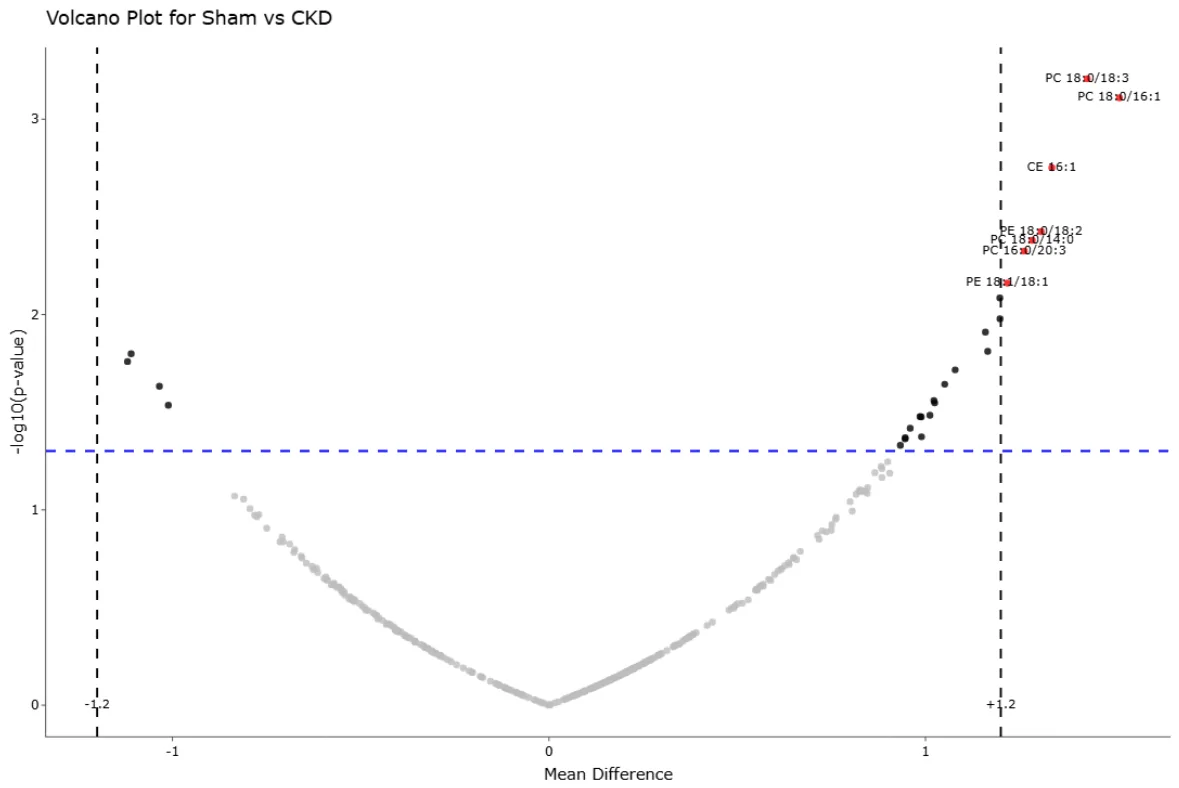
Volcano plot showing the distribution of significance of top differentially measured lipids by study group. *p*-value threshold, mean difference, and adjusted *p*-value can be explored as available options. The blue dashed line in the volcano plot indicates the statistical significance threshold (−log10(0.05)). The black dashed line indicates the mean difference threshold.

Volcano plots can be generated in the next step, aimed at identifying the top differentially measured lipids by study group (Figure S24 and Figure 6). *p*-value threshold, fold change, and adjusted *p*-value can be explored as available options. 

**Statistical Tests:** “Lipidomics Mean Calculator” presents the mean of each lipid by study group. In a lipidomic study of CKD in a mouse model of atherosclerosis [30], the mean distribution of the top 10 lipids in CKD and sham groups is presented (Figure S25). “T-Test” shows the nominal and adjusted *p*-value of a compound-by-compound *t*-test comparing the statistical differences between the study groups (Figure S26A). Figure S26A shows that PC18:0/16:1 had a significantly higher abundance in the sham as compared to the CKD group (*p* nominal = 0.0001, adjusted *p*-value = 0.0404). A PCA plot can show separation of the two groups by the incorporation of the significant lipids identified by *t*-test using the nominal or adjusted *p*-value (Figure S26B). Similarly, “One Way ANOVA” tests the differences between lipid means in more than two groups. “Correlation” provides a bivariate correlation heatmap across lipids by study group (Figure S27A–C). Comparing the bivariate correlation heatmaps in the CKD (Figure S27B) and sham groups (Figure S27C) shows global significant alterations of bivariate correlations among the studied lipids in CKD, suggesting an effect of disease state on lipid metabolic pathways.

**Figure 7:**
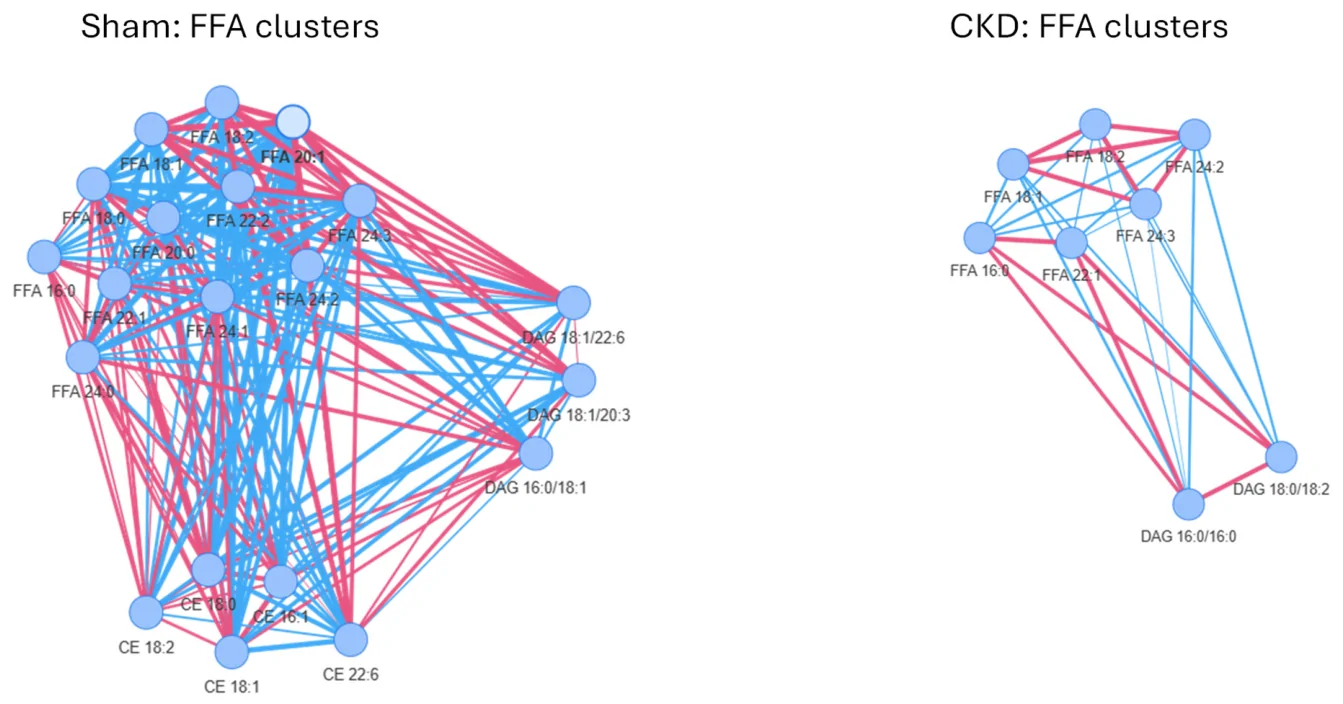
Debiased Sparse Partial Correlation (DSPC) allows comparing subnetworks of differentially correlated lipids using partial correlations. In this example, correlates of free fatty acids (FFA) in sham model of chronic kidney disease (CKD, **left panel**) shows preserved interrelationship, whereas loss of significant correlations with FFA in CKD model (**right panel**) suggests disruption of physiologic pathways. The blue edges represent negative correlation, while the pink edges represent positive correlation.

Debiased Sparse Partial Correlation (DSPC) Network generates clusters of differentially correlated lipids by study group using partial correlations (Figure S28). In this example lipidomic study [30], correlates of free fatty acids (FFA) in CKD and sham groups were compared using the DSPC approach. In CKD, significant correlates of FFA significantly diminished compared to the sham model (Figure 7), suggesting significant disruption of FFA metabolic pathways by the CKD disease state.

Partial Least Square Discriminant Analysis (PLS-DA) allows the identification of the top lipid candidates by study group (Figure S29A–D). The example study revealed the cumulative proportion of variance in the predictor matrix (R^2^X) at 0.264, and the cumulative proportion of variance in the response variable (R^2^Y), or the goodness of fit, at 0.593 with a permutation test *p*-value of 0.05. Model prediction ability by cross-validation (Q^2^) was 0.048 with a permutation test *p*-value of 0.2 (Figure S29B) and a high root mean square error of estimation (RMSEE) of 0.338. Figure S29D illustrates the top 10 candidate lipids to separate the two groups, with PC 18:0/16:1 being the top candidate, having a VIP value of 2.87 and a P1 loading score of 0.077. Overall, the borderline and/or non-significant R^2^Y and Q^2^ and high RMSEE argue against the validity of the proposed PLS-DA model in this example. Similarly, Orthogonal Partial Least Square Discriminant Analysis (OPLS-DA) is used to identify the predictive components associated with the study groups as well as the orthogonal components unrelated to group differences (Figure S30A–E).

Finally, RF can be used as a machine learning technique to rank the top lipid candidates by RF importance (Figure S31A–C). In the example lipidomic study, RF revealed an accuracy of 1 with confidence interval of 0.3976 to 1, null accuracy of 0.5 (accuracy *p*-value = 0.0625), and Cohen’s kappa of 1 (Figure S31B). Figure S31C illustrates the top 10 proposed lipids by the model, with TG54:0 as the top lipid candidate, having a mean decrease Gini value of 0.44 and a Mean Decrease Accuracy value of 3.56. An accuracy of 1 in RF is a warning sign and is an indicator of overfitting. While a perfect Cohen’s kappa score can be the best possible score, a coexistent similarly high accuracy score points to overfitting. The insignificant null accuracy of 0.5 in binary classification denotes a lack of predictivity and that the model’s predictivity is no better than a coin toss. At the end, users can download all the data or the plots generated in the working session (Figure S32).

**Validation:**

**Lipid parsing and lipid nomenclature recognition benchmark:** To systematically evaluate parsing performance, we benchmarked all platforms using the standard Metabolon lipidomic output panel containing 1102 unique lipid annotations [31] from the standard Metablon output (Table S11) against the latest software versions (Table 1). LipidAnalyst successfully recognized all 1102 lipids (100%) and accurately assigned lipid characteristics including lipid class and acyl chain information. In contrast, most of the platforms available could not handle diverse nomenclature systems. LipidSig [21] and LipidOne [22] recognized 535 and 547 lipid annotations, respectively, whereas MetaboAnalyst [20] identified only 464 lipids and did not extract lipid class or acyl chain information. LipidSuite [23] successfully parsed most lipid names but failed to recognize complex annotations such as TAG 60:10-FA22:5.

As summarized in Table 2, LipidAnalyst supports a broader range of lipid nomenclature formats than commonly used lipidomic platforms, including common lipid names, vendor-specific abbreviations, hydroxyl annotations, and complex triacylglycerol (TG) annotations.

**Statistical analysis and plot reproducibility:** To systematically evaluate the reproducibility of normalization methods, statistical analyses, and plot generation using a real-world lipidomic dataset, we analyzed the publicly available HFrEF plasma lipidomic dataset across multiple lipidomic platforms including LipidAnalyst [32]. This dataset includes human plasma samples from Heart Failure with Reduced Ejection Fraction (HFrEF) patients (n = 13) and non-HFrEF controls (n = 10).

We performed a controlled downstream analysis benchmark across multiple lipidomic platforms. To ensure comparability, the same processed data matrix was used for all platforms. First, we removed 33 features that were missing for more than half of the samples to avoid unreliable imputation. The rest of the missing values were imputed using a sample-wise KNN algorithm. The data then went through various normalization methods including log2 transformation followed by z-score standardization in one occasion, and then by sample-wise sum normalization followed by square root transformation and pareto scaling in another occasion by LipidAnalyst and MetaboAnalyst separately. The resulting processed dataset was subsequently used as the common input for benchmarking downstream analyses across all lipidomic platforms.

LipidAnalyst reproduced statistical results that were identical compared with those generated by MetaboAnalyst across different normalization methods, including identical outputs in *t*-test (Tables S1 and S5), PLS-DA (Tables S2 and S6), OPLS-DA (Tables S3 and S7), and PCA, with a Pearson correlation coefficient of 1 between outputs of the two platforms. RF results were 70% concordant (Tables S4, S5, S9 and S10). The reason for the lower concordance is that LipidAnalyst uses a user-defined training/test partition, whereas MetaboAnalyst builds the model using the full dataset, which is expected to produce differences in feature importance, and yet 70% concordance is achieved. The observation of significantly altered PE species from the LipidAnalyst results is consistent with previous lipidomic studies of pressure overload-induced heart failure, supporting the biological plausibility of our findings.

Plots: LipidAnalyst compared the PCA (Figure S34) and OPLS-DA plots (Figure S35) against MetaboAnalyst and the volcano plot (Figure S36) against LipidSuite. DMLH plots of a lipidomic study of voclosporin [31] were replicated and validated with 100% reproducibility (Figures S37–S43). DMLH accurately reflects the changes posed by various methods of sum normalization (lipid class versus sample-wise). Further sensitivity analysis revealed its robustness across various normalization methods (Figures S44–S50).

## 4. Discussion

Shotgun lipidomics was pioneered in the early 2000s by Han and Gross [33] and was established as a direct-infusion mass spectrometry approach designed to rapidly identify and quantify individual lipid molecular species based on their total carbon number and total number of double bonds in fatty acyl chains, rather than through prior chromatographic separation. Technological advancements in targeted mass spectrometry, particularly when combined with separation techniques like liquid chromatography or ion mobility spectrometry, enables the separation and identification of lipid isomers that differ in their acyl chain composition, including variations in chain length, unsaturation, and positioning (sn-position) [34,35,36]. This approach overcomes the limitations of conventional mass spectrometry in distinguishing isobaric species (compounds with the same mass but different structures). While these advances have improved the analytical capabilities of lipidomic platforms, they have also increased the need for specialized computational tools capable of processing, visualizing, and analyzing increasingly complex lipidomic datasets. To address this need, we developed LipidAnalyst, a comprehensive platform designed to facilitate lipidomic data processing, visualization, and statistical analysis.

LipidAnalyst has a flexible lipid name parsing capability, which allows rule-based shorthand parsing, matching against the LIPID MAPS database, and user-guided manual correction of parsed annotations. This functionality enables users to verify the assignment of carbon chain length and degree of unsaturation at individual Sn positions in glycerophospholipids, as well as within the sphingoid base and N-acyl fatty acid moieties of sphingolipids. In addition, LipidAnalyst reports the total number of carbons and double bonds across all lipid chains. Beyond facilitating accurate lipid annotation, this feature serves as an important quality control step and provides the structural information required for generating DMLHs.

Several existing software platforms provide functionality for lipidomic and metabolomic data analysis; however, they differ substantially in their support for lipid-specific annotation and structural parsing. For example, MetaboAnalyst [20] is a widely used platform for metabolomic data analysis, but it is primarily designed for general metabolomic workflows and lacks the lipid-specific parsing and structural annotation capabilities required for comprehensive lipidomic analyses [37]. LipidSig [21] and its companion R package, LipidSigR [21], utilize Goslin [38] for automated lipid parsing, promoting nomenclature standardization but limiting support for vendor-specific and non-standard lipid names. Similarly, LipidOne [22], LipidSuite [23], and lipidr [25] also rely on standardized lipid nomenclature prior to parsing, which may require additional curation by users. Although LipidOne [22] and MetaboAnalyst [20] include their own lipid name conversion utilities, and standalone tools such as LipidLynxX [39] converter are also available, lipid name conversion alone does not fully address the challenges of lipid parsing, particularly for vendor-specific abbreviations and complex lipid annotations. By supporting both standardized and non-standard lipid annotations, LipidAnalyst provides a more flexible framework for lipidomic data processing and quality control.

The identification of various isomers with the same mass by targeted lipidomic platforms leads to an exponentially higher number of quantified lipids as the number of acyl chains increases. For example, triacylglycerols can have numerous isoforms per mass, owing to three fatty acyl chains on their Sn1 to Sn3 glycerol carbons. Some users may prefer to combine the abundance of these isomers into a single mass. Similarly, some platforms report different adducts of the same lipids, which technically necessitates their combination into a single value. LipidAnalyst provides the ability to combine lipid isomers of a lipid with a certain mass, or different adducts of a certain lipid into a single variable, which eliminates the need for manually cleaning the data sheet and merging the corresponding species.

Normalization by internal standards is a crucial step to reduce technical variability, to improve the accuracy and consistency of peak area measurements, and to quantify the lipids in targeted platforms. Most mass spectrometry software, especially in targeted platforms, allow normalization and quantification of lipids from spectral peaks by the incorporation of authenticated internal standards with known concentrations. Therefore, the downloaded data from such a processing step can be used for further downstream normalization and analyses without any further need for internal standard normalization. However, in many instances, users may download the raw data representing the peak spectral abundance of lipidomic and internal standards for each sample prior to internal standard normalization. To that end, upon proper uploading of the corresponding internal standard data file, the users may choose a certain internal standard for each lipid class to be normalized by. For final quantification, users may further normalize the data by a user-defined constant value to account for fixed factors (dilution factor, weight, volume, etc.) or sample-specific factors (such as protein concentration).

Normalization by variable factors necessitates that the corresponding variables be listed in the metadata. While MetaboAnalyst [20] also provides metadata-based normalization options, it requires manual specification of normalization values for individual samples, which can be a prohibitive task in large datasets.

Intraclass lipids (lipid species belonging to the same chemical class, such as phospholipids or triglycerides) exhibit significantly different concentrations and molecular structures, often spanning several orders of magnitude in abundance within a single sample. While they share a common backbone or functional group, variations in fatty acid chain length, saturation, and branching lead to high diversity in their concentration, ranging from mmol/mg to nmol/mg of protein [40,41]. Such a magnitude of differences is further notable between lipid classes. Hence, it is imperative that other than sample-wise normalization, intra-lipid class normalization be considered prior to downstream analysis when supported by the underpinning plausible biology. Lipid class sum normalization expresses each lipid species as a proportion of its corresponding lipid class total, facilitating the identification of the proportional contribution of each feature, even within low-abundance lipid classes. The availability of these functions streamlines the analysis workflow and saves time for researchers. In addition to sample-wise normalization by sum and central measures of distribution, LipidAnalyst provides lipid class normalization by such measures. The logit transformation maps proportions constrained between 0 and 1 to a continuous, unbounded scale (−ꝏ to +ꝏ), enabling linear modeling on bounded data, ensuring symmetric distributions, and stabilizing variance [42]. This transformation is ideal when sum normalization (bounding data between 0 and 1) is applied. For quantified data with original scale that are not sum-normalized, other forms of data transformation such as log, square root, and cubic root are available, which can be used as appropriate. At the end, various data scaling methods can be applied for comparability.

Visualization tools in LipidAnalyst contain a number of different options. Boxplots and violin plots illustrate the distribution by sample, lipid class, subgroup of lipids within each class, or individual lipid. PCA plots reveal the separation of study groups using lipidomic data reduced to the top two principal components. Heatmaps for hierarchical clustering reorganize rows and columns to bring similar lipids together.

Another unique feature of LipidAnalyst is the ability to generate DMLH, a powerful tool for hypothesis generation and biological interpretation. Our group was the first to apply this style of class-wise chain-length × saturation visualization approach at scale in chronic kidney disease (CKD) lipidomic studies [10]. The abundance of intraclass lipids is alerted by changes in the number of carbons and double bonds, in response to environmental factors, disease state, or metabolic alterations [8,9,10,43,44,45,46]. The rationale for DMLH is to illustrate and compare the relative abundance of various lipids within each class by changing carbon number and the number of double bonds, by projecting the carbon numbers on the X-axis and the number of double bonds on Y-axis, while the heatmap is color-coded to represent the z-score standardized mean values for comparability. For lipids with more than one acyl chain, users have the option to sort the carbon numbers by chain-1, chain-2, or total carbon number to achieve the most appropriate visualization. Depending on lipid class and the pattern of lipid alteration, it may unravel underpinning mechanisms such as alterations in elongation and desaturation [10], up- or down-regulation of de novo lipogenesis [8,44], impairment of β-oxidation [8,10], alterations in phospholipase A1,2 activities [9], worsening insulin resistance [9], up-regulation of acyl-CoA synthetase 1 [30], and many others. More broadly, DMLHs enable researchers to identify biologically meaningful lipidomic patterns that may not be apparent from analyses of individual lipid species alone, making them a valuable tool for exploratory lipidomic research and hypothesis generation. One advantage of DMLH over the hierarchical clustering heatmap is that as it sorts the abundance by carbon number in X-axis and the number of double bonds in the Y-axis, it unravels the relationship by structural alteration. Unlike comparable hierarchical clustering heatmaps (for example: Supplement Figure S20), where the heatmap shows the data for individual samples, the data are shown by mean value for experimental groups, making the heatmap simpler and facilitating visual pattern recognition by structural changes as well as by experimental group. This is a greater advantage for studies with a large sample size where the inclusion of data at the individual sample level adds to the complexity of the heatmap and can potentially make pattern recognition more difficult.

The integrated statistical framework in LipidAnalyst enables comprehensive analysis of lipidomic datasets, combining differential testing, supervised modeling, and network-based analysis within a unified workflow. PLS-DA and OPLS-DA are both supervised classification methods widely used in omics studies for the separation of groups and identification of biomarkers. The primary difference is that OPLS-DA mathematically separates the variation into predictive and orthogonal (unrelated) components, resulting in much clearer, more interpretable visualizations. Overfitting is minimized by limiting model complexity through internal cross-validation for model optimization, evaluating for Q^2^Y (predictive ability) alongside R^2^Y (explained variance), applying permutation testing to assess model significance, and reducing noise through appropriate feature filtering and data processing. LipidAnalyst implements a standardized non-nested cross-validation strategy. Feature selection is conducted by post hoc selection using Variable Importance in Projection (VIP). The model robustness is assessed using cross-validation and permutation testing, while VIP scores are reported as post hoc measures of variable importance. Users are encouraged to interpret VIP values together with other statistical evidence, such as univariate significance, effect size, and biological relevance. Identification of differential lipid candidates should preferably be supported by multiple complementary metrics, such as VIP scores from the model, univariate statistical significance, and effect size, rather than relying on a single ranking criterion. [47]. There should be at least 10 biological replicates per group [47], although group or class imbalance will require a larger sample size.

RF is a highly versatile supervised machine learning algorithm that combines the outputs of multiple decision trees to reach a single, more accurate result. Overfitting is reduced through bootstrap sampling, random feature selection, and ensemble learning. LipidAnalyst uses the default classification setting of $mtry=\left\lfloor \sqrt{p}\right\rfloor$, where *p* is the number of predictor variables, together with a user-defined number of trees. Prevention of overfitting in RF can be achieved by constraining tree complexity, increasing ensembling randomness, optimizing ensemble parameters, and by noise removal. There is no universal minimum sample size requirement, but empirical studies suggest at least 25 to 200 samples to generate a stable, accurate prediction. Stability of RF importance scores can be achieved by increasing the number of trees, the use of Mean Decrease Accuracy over Gini importance, and eliminating multicollinearity.

One approach to put differentially measured significant findings into clinical context is to map the differentially measured metabolites onto known biochemical pathways, which is a commonly used method in metabolomic studies [48,49,50]. However, applying pathway-based approaches to lipidomic data remains challenging because lipid metabolic pathways are incompletely represented in existing pathway databases [51]. As an alternative to knowledge-based approaches such as pathway mapping, data-driven network methods can be used to visualize coordinated changes among groups of lipids and generate biological hypotheses. To support this type of analysis, LipidAnalyst implements the Debiased Sparse Partial Correlation (DSPC) algorithm previously developed [29] and validated [52] by our group for metabolomic and lipidomic datasets. To illustrate the application of DSPC in LipidAnalyst, we used the example dataset to generate differential correlates of free fatty acids in a mouse model of CKD (Figure 7). The network generates clusters of differentially correlated lipids by study group using partial correlations (Figure S28 and Figure 7). Application of graphical lasso-based partial correlation coefficients (as edge weights) allows sparsity and makes it suitable for network analysis where the number of metabolites exceeds the sample size [29]. By employing graphical lasso-based sparse partial correlation modeling, DSPC prioritizes direct lipid–lipid associations while minimizing indirect correlations, which is particularly advantageous in high-dimensional lipidomic datasets. As it is primarily intended for exploratory network analysis and hypothesis generation rather than predictive modeling, traditional concerns regarding model overfitting are less directly applicable. The regularization parameter (often denoted as λ) is primarily selected using statistical-theory-driven formulas [29] and allows for controlling the sparsity of the network. Network stability is not directly estimated in the current implementation, but it can be evaluated externally by subsampling, k-fold resampling, or repeated network re-estimation. DSPC requires that the minimum sample size be more than s(log p)^0.5^, where p is the number of features and s is the maximum number of true non-zero edges per node [53]. While DSPC can handle p > n, an extremely large p (e.g., thousands of metabolic features) with a very small n (e.g., sample size < 50) may yield unstable confidence intervals or reduced statistical power. The resulting networks can be exported for further refinement and functional interpretation in Metscape [54], thereby enhancing biological interpretability and hypothesis generation.

LipidAnalyst integrates multiple data processing, normalization, and visualization modules with analytical frameworks within a unified workflow compared with existing lipidomic tools. Despite its relative strengths compared to the other available packages [20,21,22,23,24,25] summarized in Table 3, such excellent tools continue to play a significant role in the landscape of lipidomic research and data analysis, with unique roles and aims pertinent to the corresponding tool. For example, LipidOne is primarily focused on functional lipidomics, lipid building-block analysis, pathway-oriented interpretation, lipid reaction/network analysis, and the use of lipid functional indices as biologically interpretable variables [22].

Despite the advantages of LipidAnalyst, it also has limitations. First, the package does not include all statistical techniques available in more advanced packages such as SAS. However, the users can download the data at different stages of data evolution and export it to other packages of their choice if analysis beyond the scope of LipidAnalyst is needed. Second, pathway analysis and gene network are not integrated into LipidAnalyst. Nevertheless, users can similarly export the analysis results from LipidAnalyst to dedicated apps such as BioPan [55], Lion/Web [56], lipid set enrichment analysis (LSEA) in LipidSig [21], LipidOne [22], and lipidr [25,55]. Sparsity imposes limitations on statistical analysis irrespective of the choice of software. LipidAnalyst includes missingness visualization and filtering tools that allow users to assess data sparsity before downstream analyses. Features with excessive missingness can be removed if appropriate, while imputation methods can be applied to moderate levels of missingness. Because highly sparse datasets may limit statistical reliability regardless of software choice, users are encouraged to evaluate data quality and sparsity patterns before interpretation. Similarly, a low sample size contributes to low study power. LipidAnalyst performs exploratory and statistical analyses regardless of sample size; however, the software does not attempt to compensate for insufficient statistical power. Users are advised to interpret significance testing results cautiously when sample sizes are small. DMLH is least informative in sparse lipid classes and is useless when the number of identified lipids within the class is reduced to one. Under this condition, alteration of lipid abundance by structural alterations within experimental groups is no longer relevant. As supervised learning approaches are prone to misuse in omics studies, users are encouraged to interpret supervised learning results together with statistical analyses and biological evidence, particularly for high-dimensional lipidomic datasets with limited sample sizes. As with all high-dimensional omics datasets, the risk of overfitting cannot be eliminated, particularly when sample sizes are limited. Therefore, we recommend that predictive models and candidate biomarkers identified by LipidAnalyst be validated using independent datasets whenever possible. For troubleshooting of common errors, please see the supplements.

## 5. Conclusions

LipidAnalyst is a powerful specialized tool for processing, normalization, visualization, and analysis of lipidomic data, empowering researchers to optimally process, visualize, and analyze lipidomic data by implementing the integration of lipid-specific processing with widely accepted statistical analyses. LipidAnalyst allows the comprehensive visualization of lipidomic data, empowering researchers to develop appropriate analytical plans accordingly.

## Figures and Tables

**Table 1:** Lipid parsing benchmark using the standard Metabolon lipidomic output.

Platform	Recognized Lipids	% Recognized
LipidAnalyst	1102/1102	100
MetaboAnalyst	464/1102	42.1
LipidSig	535/1102	48.5
LipidSuite	584/1102	53.0
LipidOne	547/1102	49.6
LipidSigR	535/1102	48.5
Lipidr	584/1102	53.0

**Table 2:** Comparison of lipid nomenclature support among commonly used lipidomic platforms.

Lipid Annotation Format	Example	Lipid-Analyst	Metabo-Analyst	Lipid-Sig	Lipid-Suite	Lipid-One	LipidSigR	Lipidr
Common lipid names	Palmitic acid, Oleic acid	✓	✓	✗	✗	✓	✗	✗
Sum composition	PC 34:2	✓	✗	✓	✓	✓	✓	✓
Molecular species	PC 16:0_18:2, PC 16:0/18:2	✓	✓	✓	✓	✓	✓	✓
Vendor-specific abbreviations	DCER 18:0	✓	✗	✗	✓	✗	✗	✓
Complex TAG annotation	TAG 60:10-FA22:5	✓	✗	✗	✗	✗	✗	✗
O-/P- ether lipid notation	PC O- 16:0/16:1	✓	limited	✓	limited	✓	✓	limited

**Table 3:** Comparison of LipidAnalyst with existing lipidomic analysis tools.

Feature	LipidAnalyst	MetaboAnalyst	LipidSig	LipidSuite	LipidOne	LipidSigR	Lipidr
**User interface**	Web-based GUI	Web-based GUI	Web-based GUI	Web-based GUI	Web-based GUI	An R package	An R package
Supported input format	.csv/.tsv/.xlsx	.csv/.tsv/.zip, mzTab, xlsx from Metabolon	.csv/.tsv/.xlsx	numerical matrix (.csv), skyline export, and mwTab	.csv/.txt	Lipid abundance matrix (.csv/.txt)	numerical matrix (.csv), skyline export, and mwTab
Feature filtering	Yes	Yes	Limited (restricted to filtering features with high missingness)	Lipids can be filtered by their %CV	No	Limited (restricted to filtering features with high missingness)	Lipids can be filtered by their %CV
Customizable data imputation	Yes	Limited (no group-specific missing-value imputation)	Limited (no group-specific missing-value imputation)	Limited (no group-specific missing-value imputation)	No	Limited (no group-specific missing-value imputation)	Limited (no group-specific missing-value imputation)
Merging duplicate lipids and adduct variants	Yes	No	No	Limited (cannot aggregate across different adducts)	No	No	Limited (cannot aggregate across different adducts)
Lipid Parsing	Yes	Limited (name matching against LIPID MAPS, no chain information provided)	Limited (constrained by supported nomenclature rules)	Limited (constrained by supported nomenclature rules)	Yes (no parsing validation reference provided)	Limited (constrained by supported nomenclature rules)	Limited (constrained by supported nomenclature rules)
Method coverage of normalization	Yes	Yes	Yes	Yes	No	Yes	Yes
Internal standard normalization	Yes	No	No	Yes	No	No	Yes
Normalization by user defined factors	Yes	Yes (require manual input for each sample)	No	No	No	No	No
Lipid class sum normalization	Yes	No	No	No	No	No	No
Differential Mean Lipid Heatmap	Yes	No	No	No	No	Yes, but only total carbon length is supported on the X-axis, limiting flexibility for multi-chain lipids.	No
DSPC network	Yes	Yes	No	No	No	No	No

## Data Availability

The data presented in this study are openly available in this software as example files and is published previously [30].

## Supplementary Materials

The following supporting information can be downloaded at: https://www.mdpi.com/article/10.3390/metabo16080526/s1, Figure S1: Welcome to LipidAnalyst. Figure S2: Users may upload an example lipidomic data by clicking on “Load Example Lipidomic Data” or upload their own file. Figure S3: Upon clicking on “Load Example Lipidomics Data” (red arrow), the data file is opened and can be viewed. Figure S4: Metadata provides the necessary information about the grouping variables and how they are linked to the lipidomic and internal standard data files through the shared research identification number. Figure S5: Users may upload the example internal standards data file by clicking on “Load Example Internal Standard Information” or simply upload their own internal standards data file. Figure S6: Data filtering allows eliminating noise and irrelevant features. Figure S7: Missing Value Imputation. Figure S8: Illustration of missing value heatmap can be achieved by clicking on “View Missing Value Heatmap” tab. Figure S9: To impute the general missing values, users may choose various methods including “KNN-featurewise”, “KNN-samplewise”, “median”, “mean”, and “Limit of Detection (LoD) 1/5 minimum value” selecting number of nearest neighbors for the imputation methods. Figure S10: Combine and Data Integration. Figure S11: Lipid Parsing. Figure S12: After clicking on “Parse Lipid Names”, further lipid characteristics for each lipid appear in adjacent columns. Figure S13: Data Preview. Figure S14: Quantification by Internal Standard. Figure S15: Upon normalization by internal standards, the normalized data can be viewed under “Normalized Data Preview”. Figure S16A: Quantification by User Defined Factor. Figure S16B: Quantification by User Defined Factor: Similarly, users may further normalize the data by metadata. Figure S17: Further normalization can be pursued. Figure S18: Global distribution boxplot. Figure S19A: PCA plot. Figure S19B: 3D PCA plot. Figure S20: Heatmap and Hierarchical Clustering. Figure S21: Differential Mean Lipid Heatmap (DMLH). Figure S22: Class Level Lipid Comparison. Figure S23: Individual Lipid Comparison. Figure S24: Volcano plots. Figure S25: Lipidomics Mean Calculator. Figure S26A: T-test. Figure S26B: T-test PCA plot. Figure S27A: Correlation plot. Figure S27B: Bivariate correlation heatmap for CKD group. Figure S27C: Bivariate correlation heatmap for Sham group. Figure S28: DSPC network. Figure S29A: PLS-DA (Partial Least Square Discriminant Analysis). Figure S29B: PLS-DA Model Performance and Validation Metrics. Figure S29C: PLS-DA overview plot and permutation plot. Figure S29D: The PLS-DA Variable Importance in Projection (VIP) plot. Figure S30A: OPLS-DA (Orthogonal Partial Least Square Discriminant Analysis). Figure S30B: OPLS-DA Model Performance and Validation Metrics. Figure S30C: OPLS-DA overview plot and permutation plot. Figure S30D: OPLS-DA score plot and outlier plot. Figure S30E: OPLS VIP plots and VIP table. Figure S31A: Random Forest model. Figure S31B: Random Forest Model Result Output. Figure S31C: Random Forest feature Importance table. Figure S31D: Dot plots for top 20 features by Random Forest Importance. Figure S32: Downloading. Figure S33: Recommended parameters settings. Figure S34: Comparison of PCA Results Generated by LipidAnalyst and MetaboAnalyst Using the Processed HFrEF Dataset. Figure S35: Comparison of OPLS-DA Score Plots Generated by LipidAnalyst and MetaboAnalyst Using the Processed HFrEF Dataset. Figure S36: Comparison of Volcano Plots Generated by LipidAnalyst and LipidSuite Using the Processed HFrEF Dataset. Figure S37: Replication of DMLH of free fatty acids (FFA). Figure S38: Replication of DMLH of acylcarnitines (AC). Figure S39: Replication of DMLH of triacylglycerols (TG). Figure S40: Replication of DMLH of monoacylglycerols (MAG). Figure S41: Replication of DMLH of cholesteryl esters (CE). Figure S42: Replication of DMLH of lysophosphatidylcholines (LPC). Figure S43: Replication of DMLH of lysophosphatidylethanolamines (LPE). Figure S44: Sensitivity analysis of DMLH of phosphatidylethanolamine-O (PE-O) with lipid class sum normalization, logit transformation, and auto-scaling. Panel A represents the chronic kidney disease group. Panel B represents the sham group. Figure S45: Sensitivity analysis of DMLH of phosphatidylethanolamine-O (PE-O) with lipid class mean normalization, log transformation, and auto-scaling. Panel A represents the chronic kidney disease group. Panel B represents the sham group. Figure S46: Sensitivity analysis of DMLH of phosphatidylethanolamine-O (PE-O) with lipid class mean normalization, square root transformation, and auto-scaling. Panel A represents the chronic kidney disease group. Panel B represents the sham group. Figure S47: Sensitivity analysis of DMLH of phosphatidylethanolamine-O (PE-O) with lipid class mean normalization, cubic root transformation, and auto-scaling. Panel A represents the chronic kidney disease group. Panel B represents the sham group. Figure S48: Sensitivity analysis of DMLH of phosphatidylethanolamine-O (PE-O) with lipid class median normalization, loge transformation, and auto-scaling. Panel A represents the chronic kidney disease group. Panel B represents the sham group. Figure S49: Sensitivity analysis of DMLH of phosphatidylethanolamine-O (PE-O) with lipid class median normalization, square root transformation, and auto-scaling. Panel A represents the chronic kidney disease group. Panel B represents the sham group. Figure S50: Sensitivity analysis of DMLH of phosphatidylethanolamine-O (PE-O) with lipid class median normalization, cube root transformation, and auto-scaling. Panel A represents the chronic kidney disease group. Panel B represents the sham group. Table S1: Comparing the *t*-test *p* values of the top 112 identified lipids in LipidAnalyst output with that in MetaboAnalyst output shows reproducuble and identical results. Table S2: Comparing the VIP scores of the identified lipids using PLS-DA analysis in LipidAnalyst output with that in MetaboAnalyst output shows reproducuble and identical results. Table S3: Comparing the VIP scores of the identified lipids using OPLS-DA analysis in LipidAnalyst output with that in MetaboAnalyst output shows reproducuble and identical results. Table S4: Comparing the Mean Decrease Accuracy scores of the identified lipids in Using Random Forest in LipidAnalyst output with that in MetaboAnalyst. Table S5: Concordance of Random Forest Output in LipidAnalyst and MetaboAnalyst. Table S6: Comparing the *t*-test *p* values of the top 39 identified lipids in LipidAnalyst output with that in MetaboAnalyst output shows reproducuble and identical results. Table S7: Comparing the VIP scores of the identified lipids using PLS-DA analysis in LipidAnalyst output with that in MetaboAnalyst output shows reproducuble and identical results. Table S8: Comparing the VIP scores of the identified lipids using OPLS-DA analysis in LipidAnalyst output with that in MetaboAnalyst output shows reproducuble and identical results. Table S9: Comparing the Mean Decrease Accuracy scores of the identified lipids in Using Random Forest in LipidAnalyst output with that in MetaboAnalyst. Table S10: Concordance of Random Forest Output in LipidAnalyst and MetaboAnalyst. Table S11: Sample Lipid Name from Metabolon Output.
