# Supplementary material for: LipidAnalyst: A Comprehensive Tool for Lipidomic Data Visualization and Analysis
Source: Metabolites. 2026 Jul 25;16(8):526. doi: 10.3390/metabo16080526 (PMC13515139; doi:10.3390/metabo16080526)
Supplement: Supplementary file 1 [file metabolites-16-00526-s001.zip › Supplement.pdf]

Supplement Methods:

Correlation Heatmap: Correlation heatmaps are generated based on Pearson correlation coefficients calculated between lipid features across samples. The coefficients (r, ranging from −1 to +1) reflect the strength and direction of linear associations. To assess statistical significance, correlations can be transformed using Fisher's z transformation and tested against zero correlations.

DSPC: Debiased Sparse Partial Correlation (DSPC) [1] is a statistical framework designed to infer sparse and interpretable molecular networks from high-dimensional omics data such as lipidomics.

Prior to network construction, the input matrix is converted into a numeric matrix and subjected to quality filtering. Lipid variables containing non-finite values (NA, NaN, or Inf) or zero variance are removed from the analysis. Variables are then standardized using z-score scaling when necessary to ensure comparable distributions across lipid species and to satisfy the assumptions of sparse precision matrix estimation.

Sparse network estimation is performed using the graphical lasso algorithm implemented in the glasso (version 1.11) package in R, followed the framework described in the original DSPC paper [1]. Regularization is applied to reduce network complexity and minimize spurious associations in high-dimensional datasets. The regularization parameter (λ) is determined using a data-dependent default based on sample size and variable number. A debiased precision matrix correction step is applied following graphical lasso estimation. Partial correlations representing direct lipid-lipid association are derived from the de-biased precision matrix. Statistical significance for each edge is assessed using two-sided hypothesis testing, and p-values are adjusted using the Benjamini–Hochberg false discovery rate (FDR) correction method.

The final DSPC output is generated as an edge list containing lipid pairs, partial correlation coefficients, raw p-values, and FDR-adjusted q-values. The resulting network can be further visualized within LipidAnalyst. The edge list can also be exported for downstream analysis in external network visualization tools such as Cytoscape.

DSPC-derived networks are further divided into subnetworks using Leiden community detection implemented with igraph (version 2.2.3) and leidenAlg (version 1.1.6) in R. Non-zero DSPC edges are retained, and absolute partial correlation values are used as edge weights for clustering. Small clusters below a user-defined node threshold are excluded. Subnetworks are visualized using visNetwork (version 2.1.4), where nodes represent lipid species and edges represent DSPC-derived partial correlations, with edge color indicating association direction and edge width scaled by association strength.

PLS-DA: PLS-DA is performed using the ropls (version 1.38.0). Partial Least Squares Discriminant Analysis (PLS-DA) is a supervised multivariate statistical method that identifies features that best discriminate between predefined groups. The number of predictive components is set to 1. Model quality is evaluated using R2X, R2Y, and Q2 values obtained through non-nested cross-validation. Permutation testing is performed to assess model robustness. The number of permutations and the number of cross validation folds are specified by users. Variable Importance in Projection (VIP) scores are calculated to estimate the contribution of individual lipids to group discrimination.

OPLS-DA: OPLS-DA is performed using the ropls (version 1.38.0). Orthogonal Partial Least Squares Discriminant Analysis (OPLS-DA) extends PLS-DA by separating predictive variation from orthogonal variation unrelated to group classification. The number of predictive components is fixed at 1. The number of orthogonal components was not manually specified but was determined automatically by the algorithm using non-nested cross-validation. Model quality is evaluated using R2X, R2Y, and Q2 values obtained through cross-validation. Permutation testing is performed to assess model robustness. The numbers of permutations and cross-validation folds are specified by users. VIP scores are calculated to estimate the contribution of individual lipids to group discrimination.

Random Forest: Random Forest analysis is implemented using the randomForest (version 4.7.1.2) package, with the caret (version 7.0-1) package used for data partitioning and model evaluation. Random Forest model is an ensemble machine learning technique that builds multiple decision trees using bootstrap sampling and random feature selection to improve predictive performance and reduce overfitting. Each decision tree is trained on a bootstrap sample of the training data, and the final class prediction was determined by majority voting across all trees. The number of trees and training/testing data partition settings are specified by users. The number of predictors randomly evaluated at each split (mtry) is set to the default value of $\lfloor\sqrt{p}\rfloor$ for classification, where p is the number of lipids in the dataset. A fixed random seed was used to ensure reproducibility. Model performance is evaluated using Accuracy, AccuracyLower, AccuracyUpper, AccuracyNull, AccuracyPValue, and McNemar's test p-value. Feature importance is assessed using Mean Decrease Gini and permutation-based Mean Decrease Accuracy calculated from out-of-bag (OOB) samples.

Benchmark: To evaluate implementation consistency, the same preprocessed dataset was uploaded to both LipidAnalyst and MetaboAnalyst using identical statistical settings whenever possible. Numerical outputs including t test p-values, PLS-DA VIP scores, and OPLS-DA VIP scores were compared using Pearson correlation coefficients. Random Forest agreement was assessed by overlap among the top 20 features ranked by Mean Decrease Accuracy rather than exact numerical correspondence.

Reference:

Basu, S.; Duren, W.; Evans, C.R.; Burant, C.F.; Michailidis, G.; Karnovsky, A. Sparse network modeling and metscape-based visualization methods for the analysis of large-scale metabolomics data. *Bioinformatics* **2017**, *33*, 1545-1553, doi:10.1093/bioinformatics/btx012.

Supplement Figures:

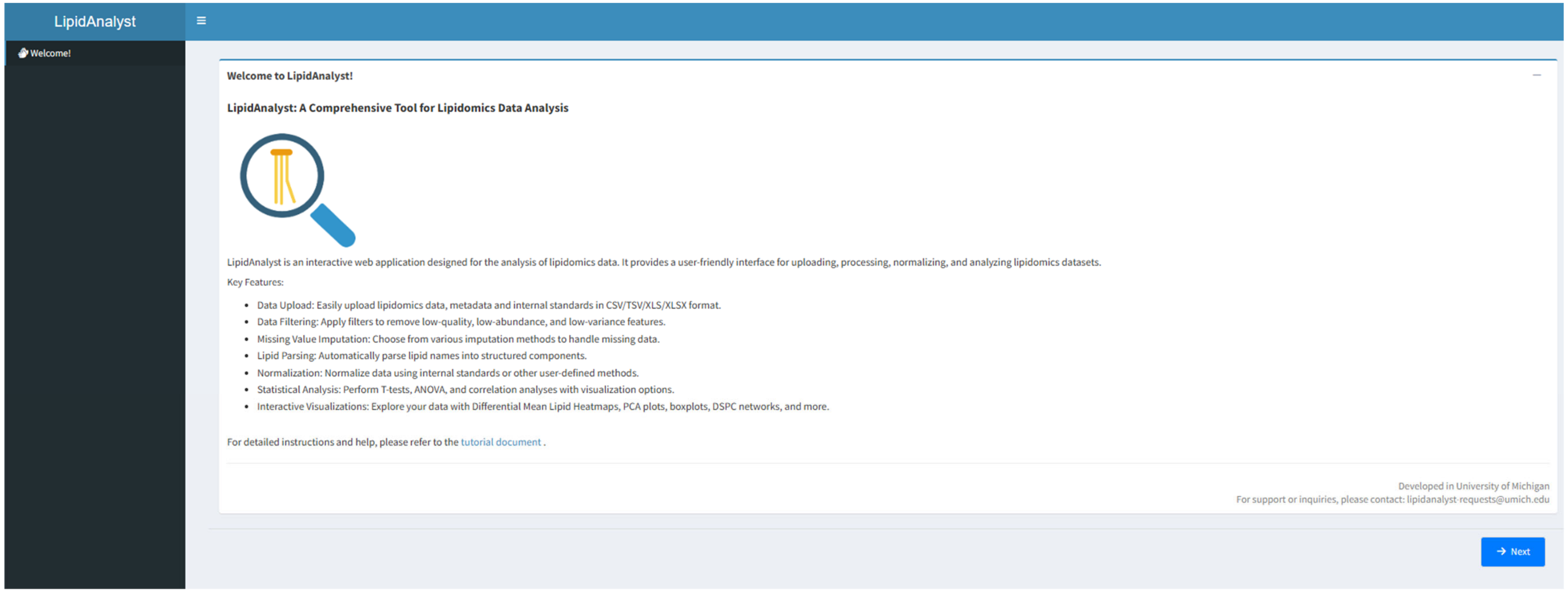

Figure S1: Welcome to LipidAnalyst. After reviewing the introduction, and “tutorial document” click on “Next” to move forward.

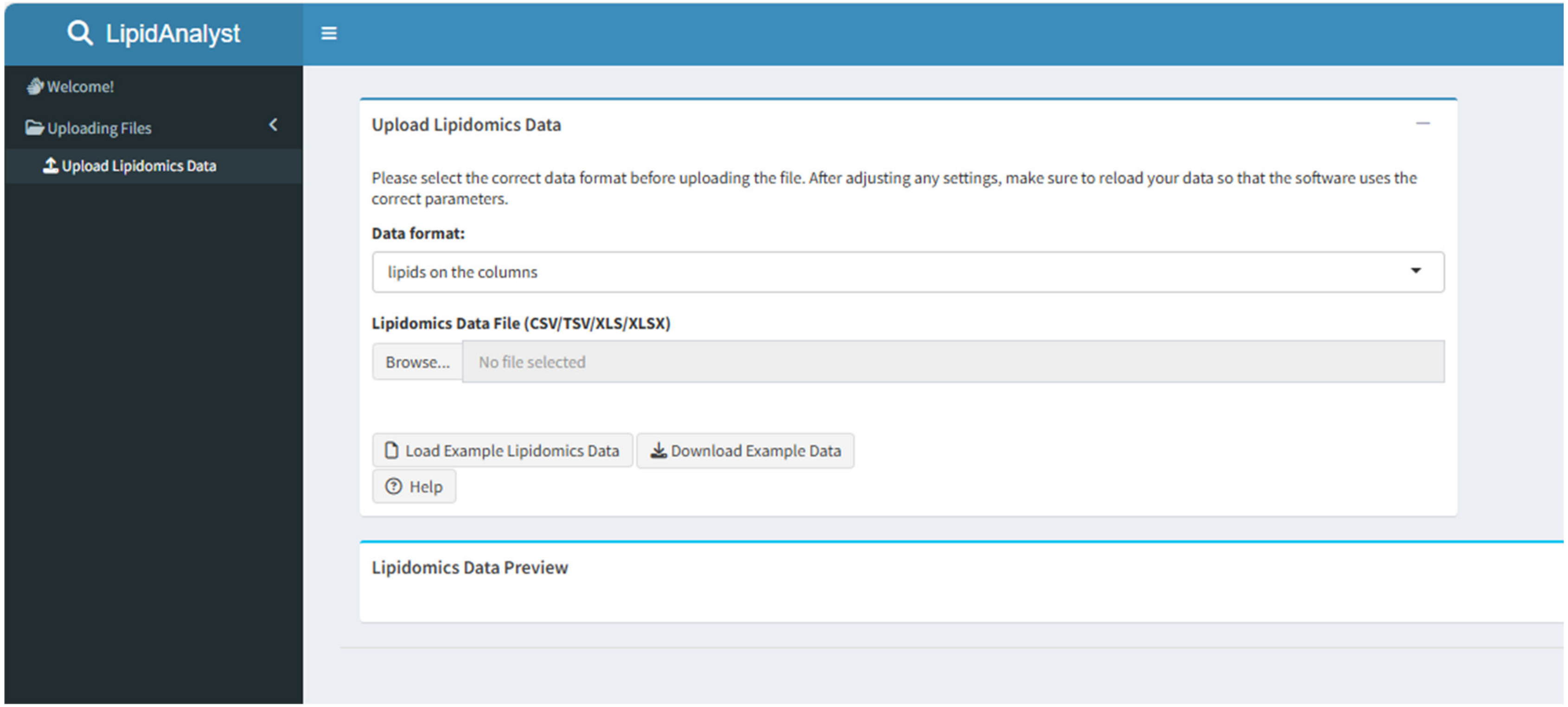

Figure S2: Users may upload an example lipidomic data by clicking on “Load Example Lipidomic Data” or upload their own file. “Data format” can be used to specify if the lipids are on the rows or the columns. When the data is uploaded, it can be viewed under “Lipidomics Data Preview”. “Help” tab provides additional information on lipidomic data and accepted formats.

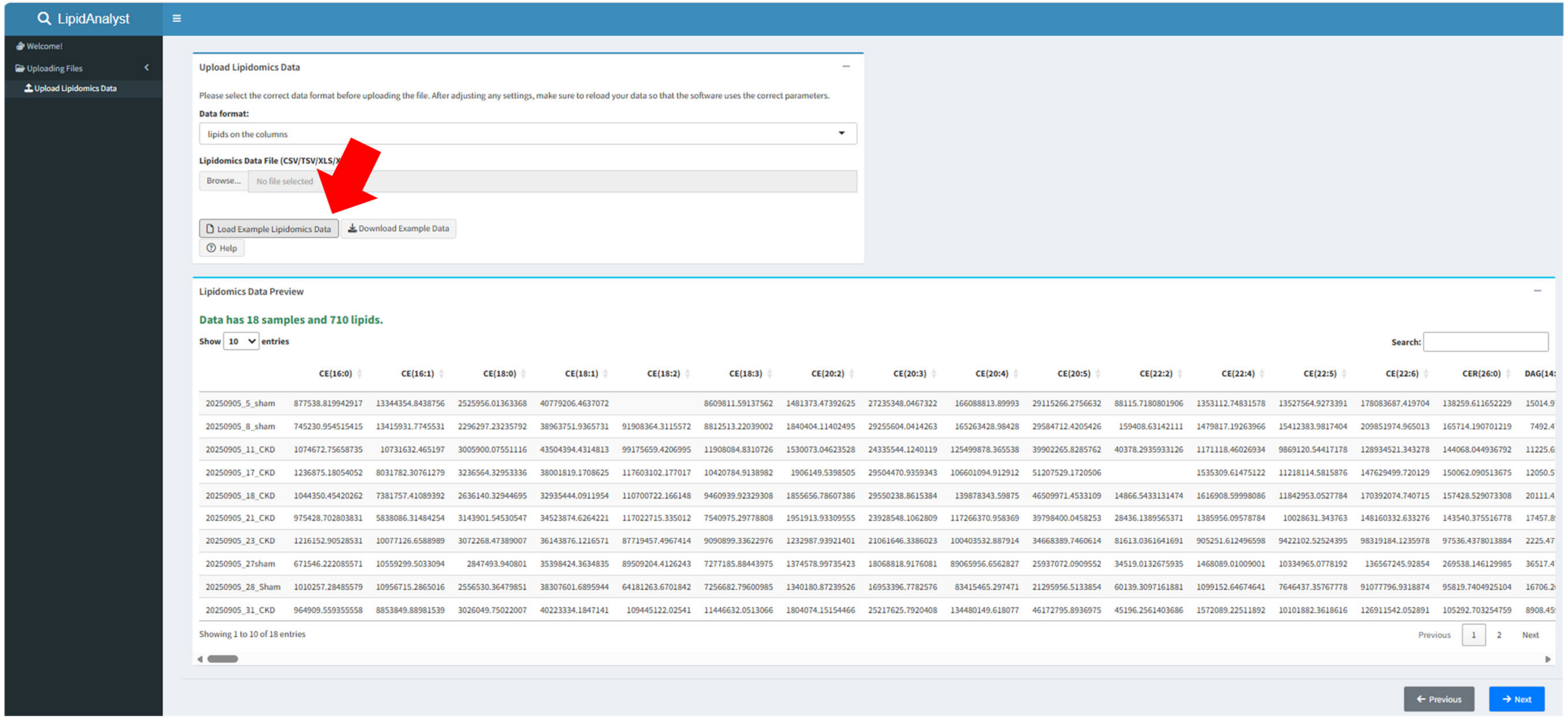

Figure S3: Upon clicking on “Load Example Lipidomics Data” (red arrow), the data file is opened and can be viewed under “Lipidomics Data Preview” as above. “Help” tab provides additional information on lipidomic data and accepted formats. Click Next to move forward.

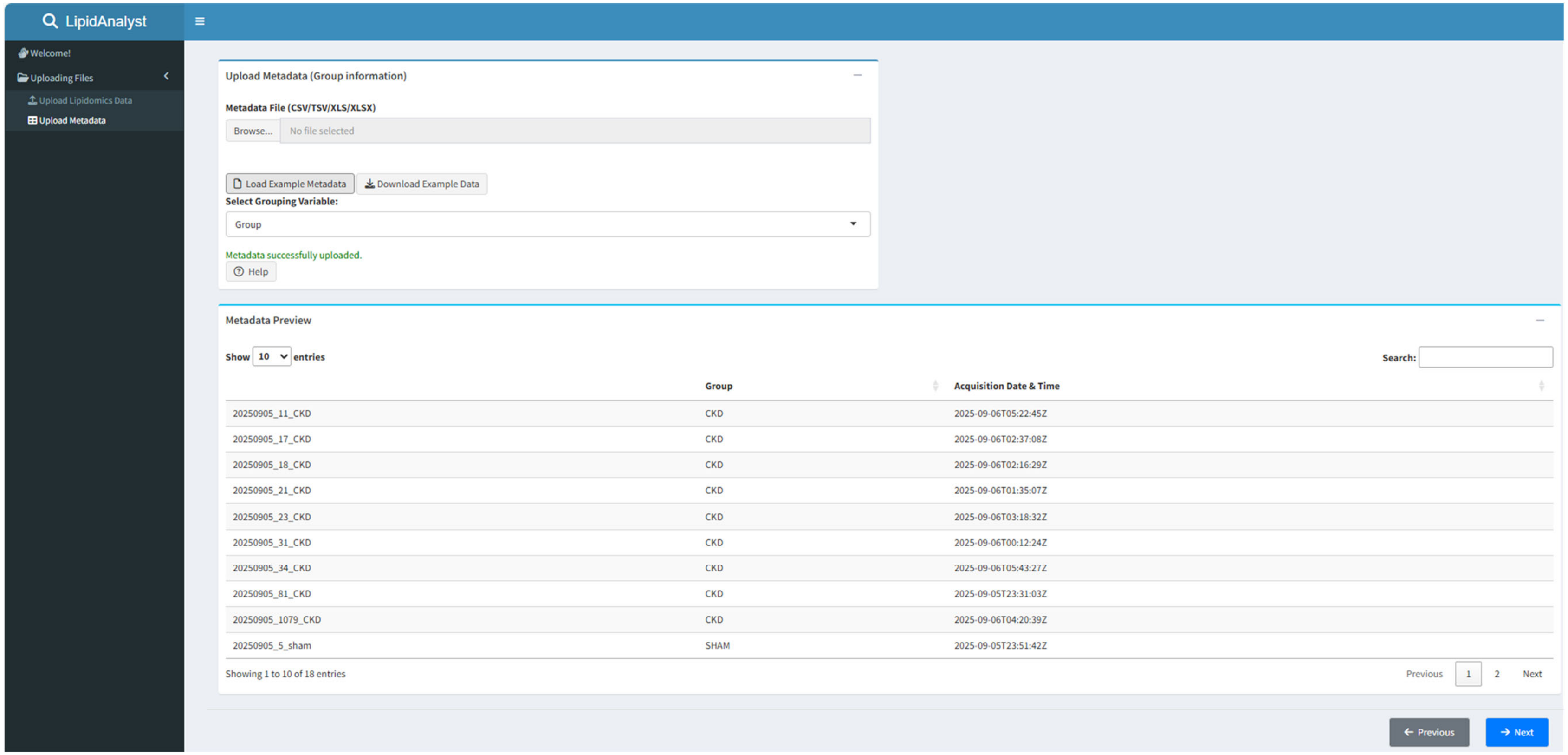

Figure S4: Metadata provides the necessary information about the grouping variables and how they are linked to the lipidomic and internal standard data files through the shared research identification number. Users can load the example metadata by clicking on “Load Example Metadata” or upload their own metadata. For metadata with more than one grouping variable, the users may select the grouping variable from the “Group” box for the downstream analysis. “Help” tab provides additional information on metadata and accepted formats. Click Next to move forward.

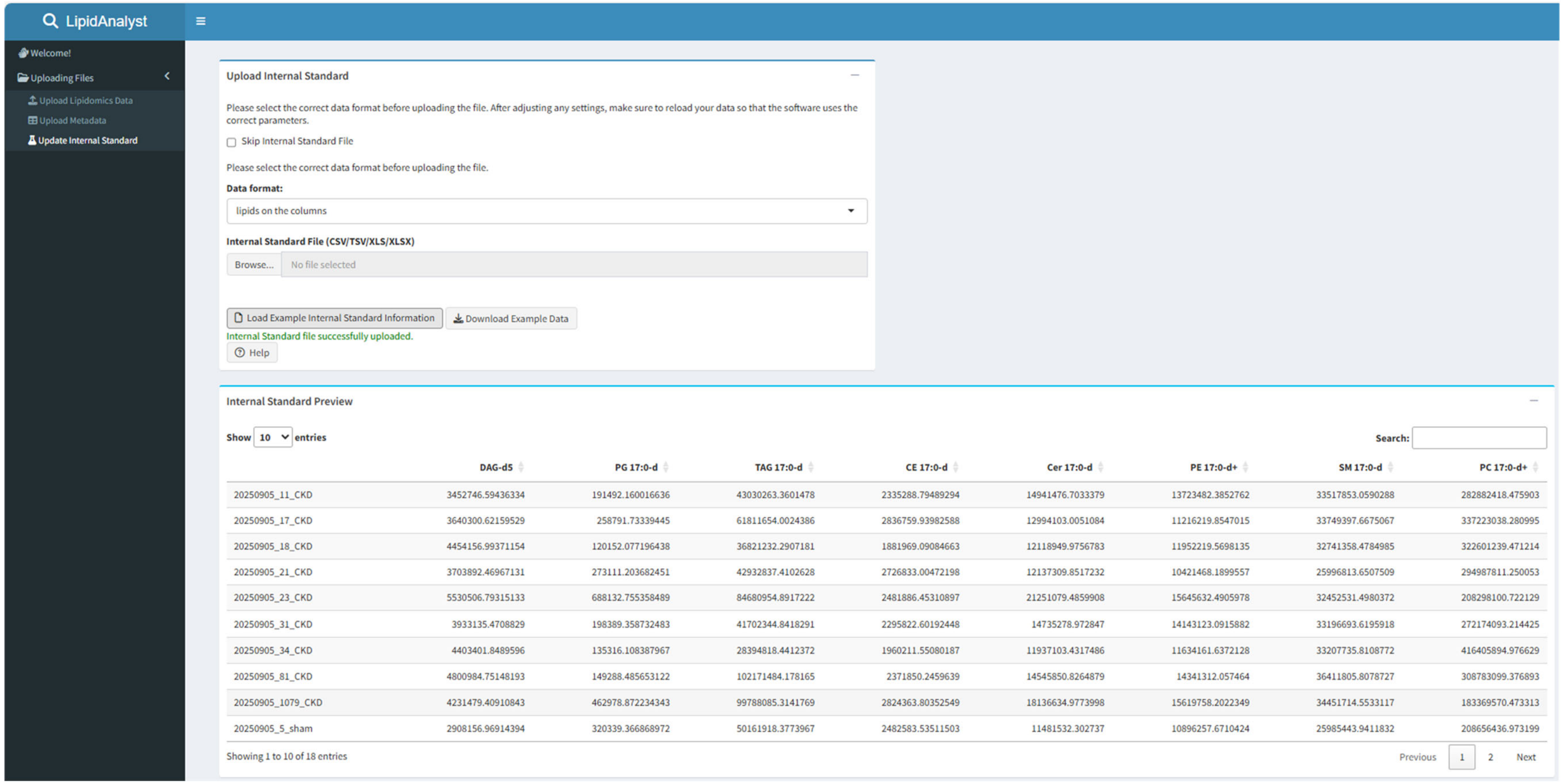

Figure S5: Users may upload the example internal standards data file by clicking on “Load Example Internal Standard Information” or simply upload their own internal standards data file. This step can be skipped if the uploaded lipidomic data is already normalized by internal standards. “Data format” section can be used to specify if the internal standards are on the rows or the columns. “Help” tab provides additional information on Internal Standards data file and accepted formats. Once loaded, the corresponding data can be viewed under “Internal Standard Preview”. Click Next to move forward.

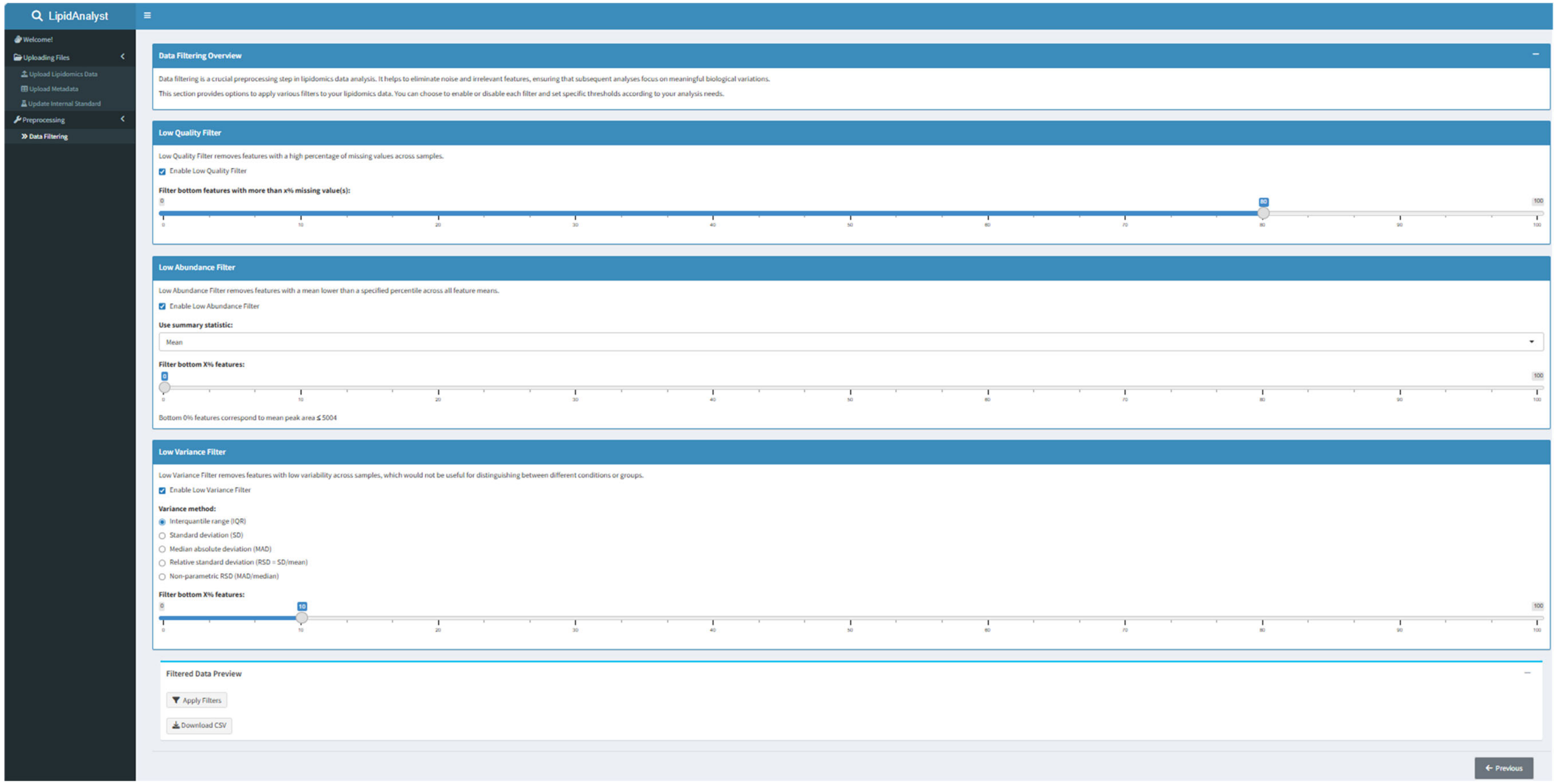

Figure S6: Data filtering allows eliminating noise and irrelevant features. “Low Quality Filter” removes features with missing data above a certain threshold which can be specified by the user. This section can be skipped by demarking “Enable Low Quality Filter”. “Low Abundance Filter” removes features with a mean or median below a level prespecified by the user. This section can be skipped by demarking “Enable Low Abundance Filter”. “Low Variance Filter” removes features with low variability across samples using a user specified threshold. The variance methods include “Interquartile Range (IQR)”, Standard deviation (SD)”, “Median Absolute Deviation (MAD)”, “Relative Standard Deviation (RSD = SD/mean)”, and “Non-parametric RSD = MAD/median). This section can be skipped by demarking “Enable Low Variance Filter”. Once the proper filtering and thresholds are selected, click on “Apply Filter” tab to implement the filtering, and then click on “Next” to move forward.

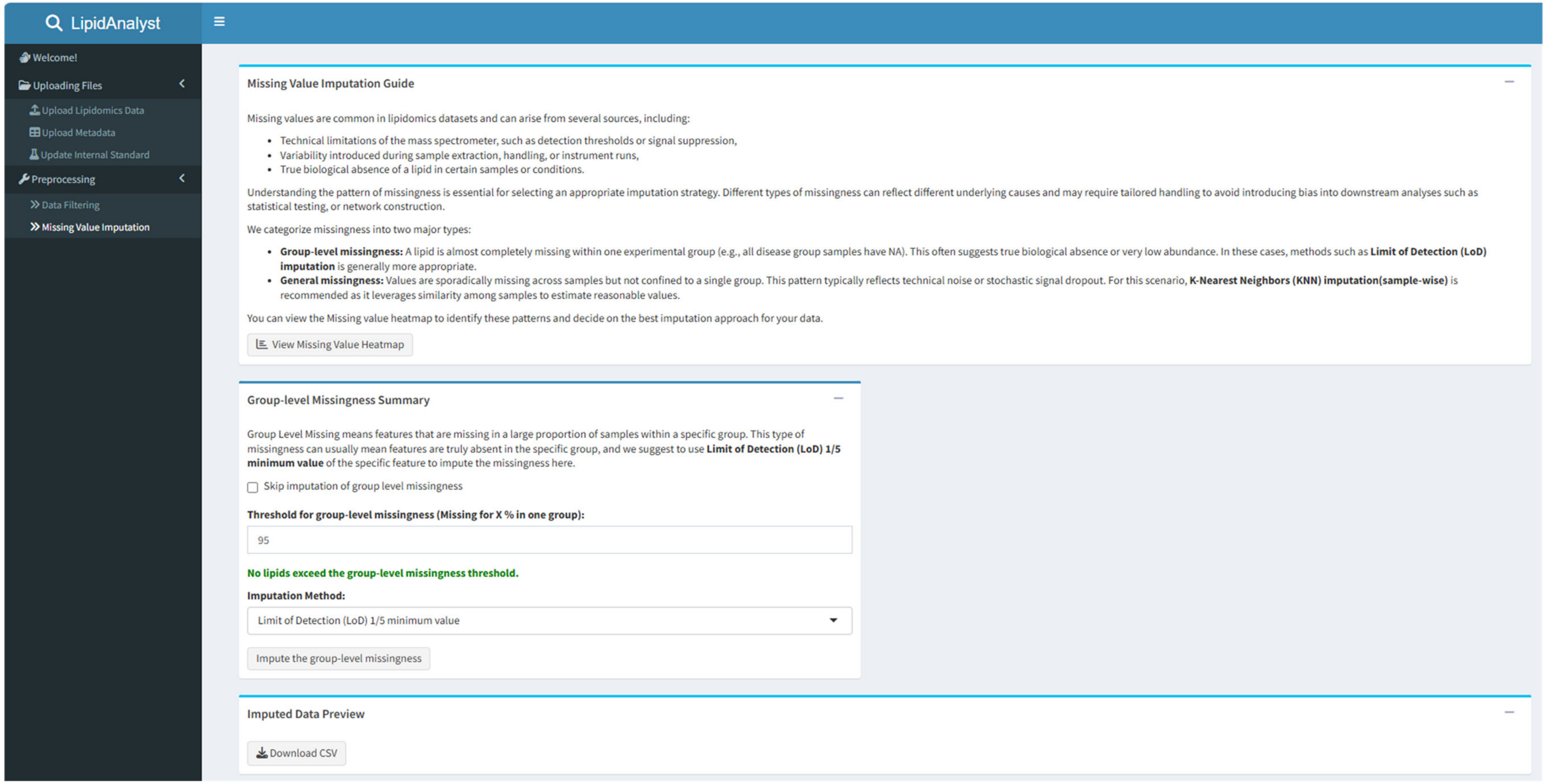

Figure S7: Missing Value Imputation: To illustrate a heatmap of missing values click on “View Missing Value Heatmap”. To impute the group-level missing values click on “Impute the group-level missingness” tab. Users may modify the threshold for group-level missingness by entering the desired value. Various imputation techniques including “Limit of Detection (LoD) 1/5 minimum value”, or “Limit of Detection (LoD) ½ minimum value”. Users may decide to skip this section by selecting the “Skip imputation of group level missingness” box.

Note:
- LoD 1/5 minimum value: replaces missing with 1/5 of featurewise minimum value.
- LoD 1/2 minimum value: replaces missing with 1/2 of featurewise minimum value.

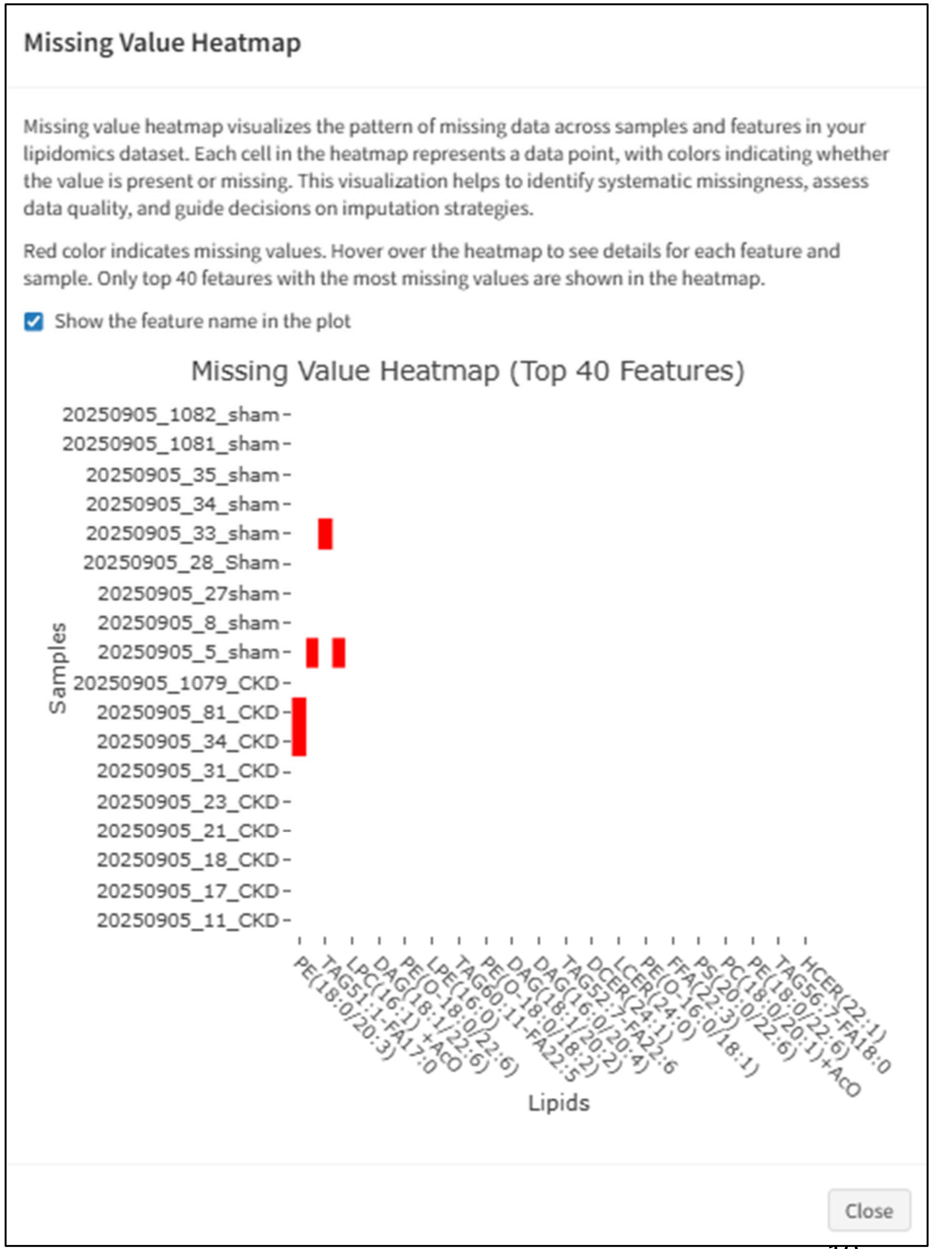

Figure S8: Illustration of missing value heatmap can be achieved by clicking on “View Missing Value Heatmap” tab above (Figure S7).

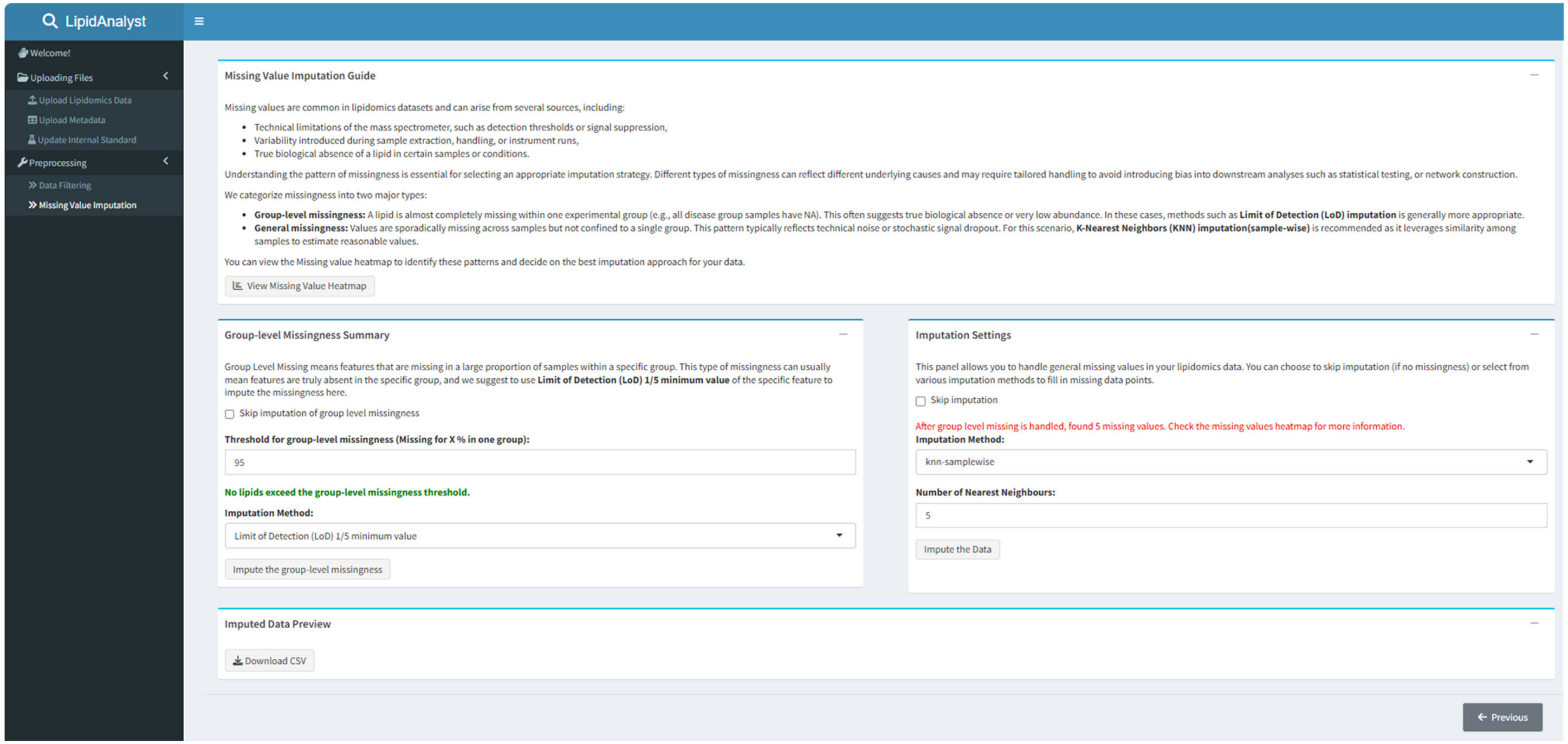

Figure S9: To impute the general missing values, users may choose various methods including “KNN-featurewise”, “KNN-samplewise”, “median”, “mean”, and “Limit of Detection (LoD) 1/5 minimum value” selecting number of nearest neighbors for the imputation methods. Users may skip this step by selecting the “Skip imputations” box for general imputation if it does not apply. After clicking on “Impute the Data”, the imputed data can be viewed under the “Imputed Data Preview”.

Note:

- KNN is suggested for large dataset.
- Use 1/5 minimum value for targeted lipidomics where missing = below detection.
- Choose the skip check box if there are no missing values.

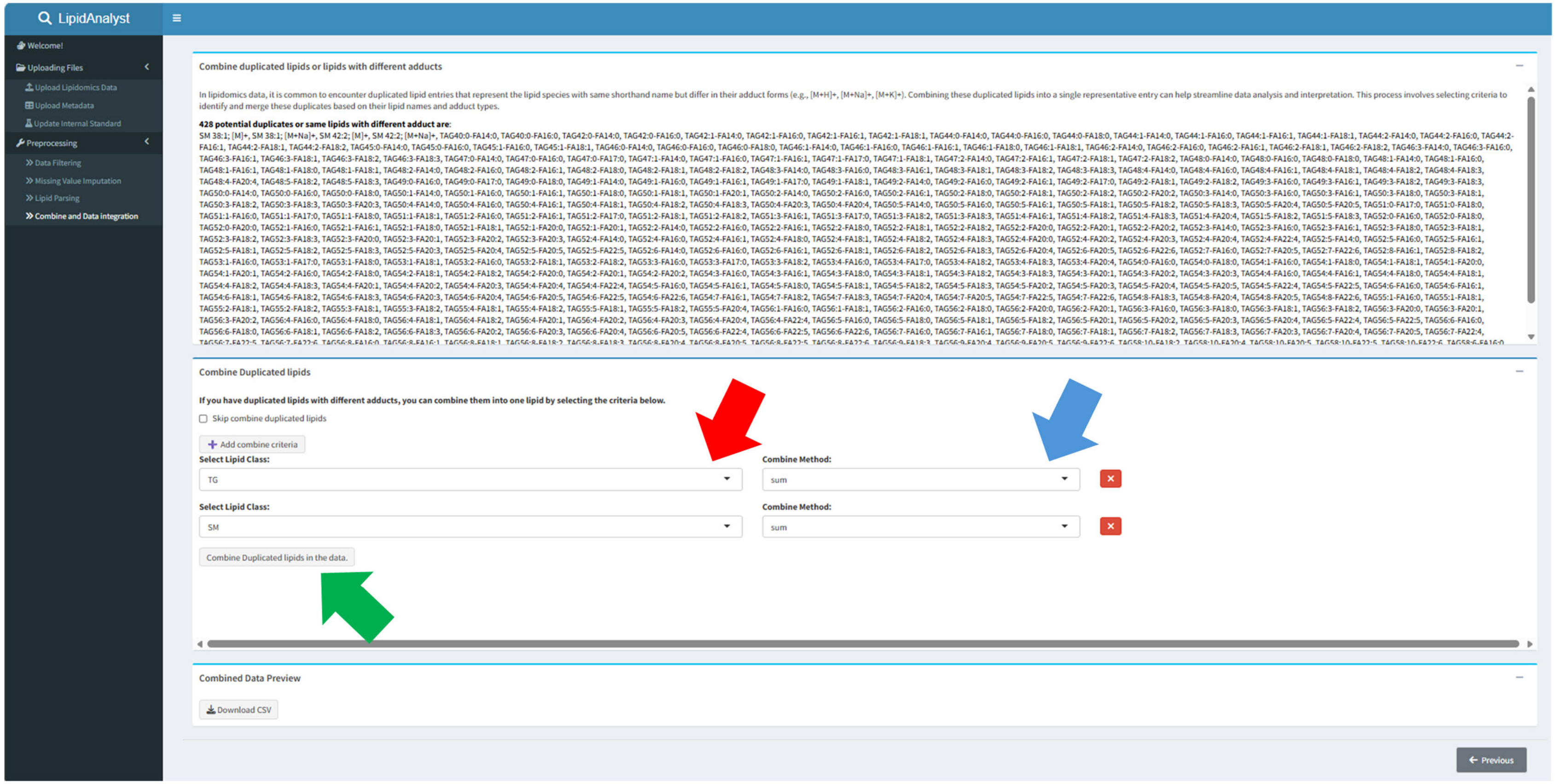

Figure S10: Combine and Data Integration: Some lipidomic platforms measure the same lipid with a different adduct. For example, SM38:1; [M]+ and SM38:1; M+Na]+ as above. Similarly, some targeted platforms are capable of measuring different isoforms of triacylglycerols. For example, TAG48:0 represented three isoforms with different acyl chains at Sn1 carbon including TAG48:0-FA14:0, TAG48:0-FA16:0, and TAG48:0-FA18:0 as shown above. In this section users have the option to combine different adducts of the same lipid or combine different isoforms of the same molecule into one feature. First, the corresponding lipid class should be selected (red arrow). Then proper combining method should be selected through the “Combine Methods” (blue arrow), as appropriate. These methods include mean, median, sum, maximum, and minimum. Then click on “Combine Duplicated lipids in the data”. Then the new dataset can be viewed under “Combined Data Preview”. Then click on blue “Next” to move forward.

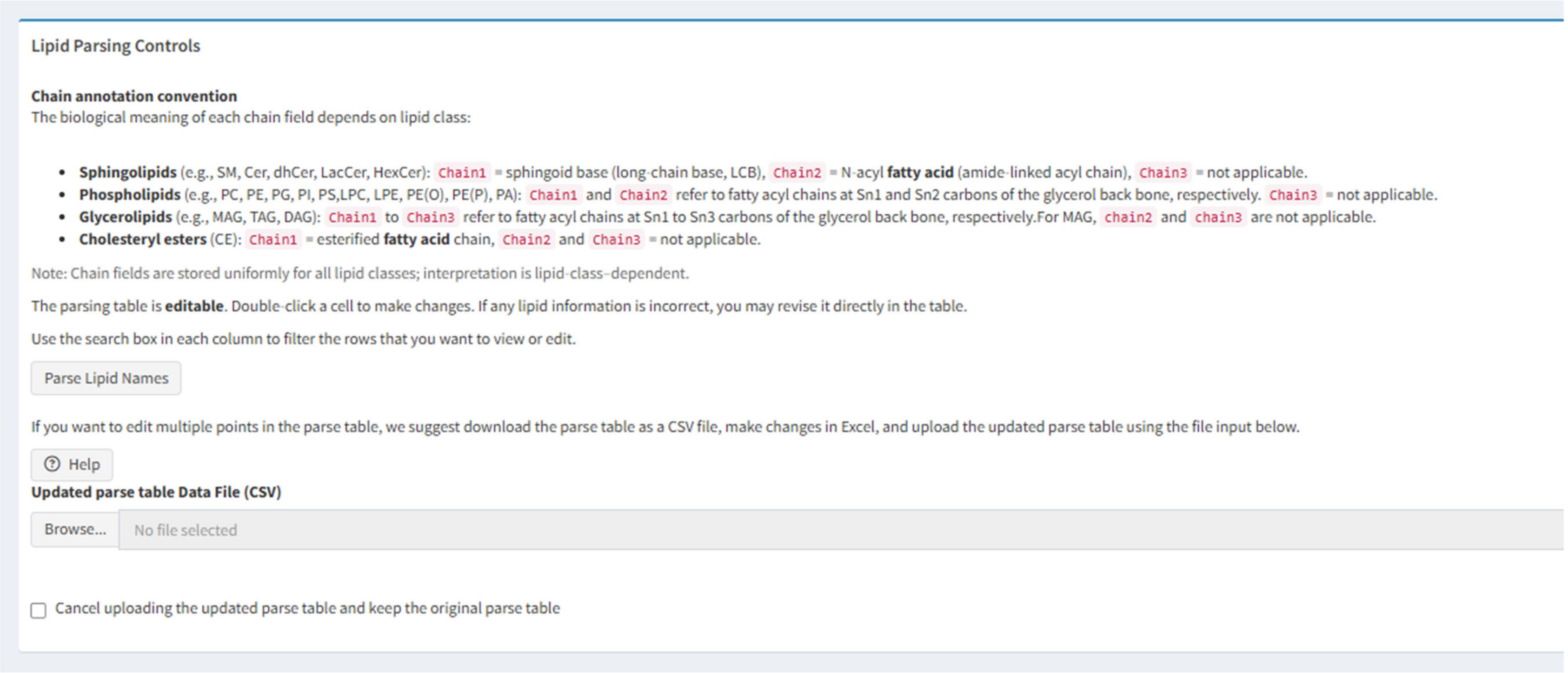

Figure S11: Lipid Parsing provides the opportunity to verify the number of carbons and double bonds by acyl chain, and to confirm the formula as a quality control measure and a base for downstream visualization techniques. Click on “Parse Lipid Names” to move forward.

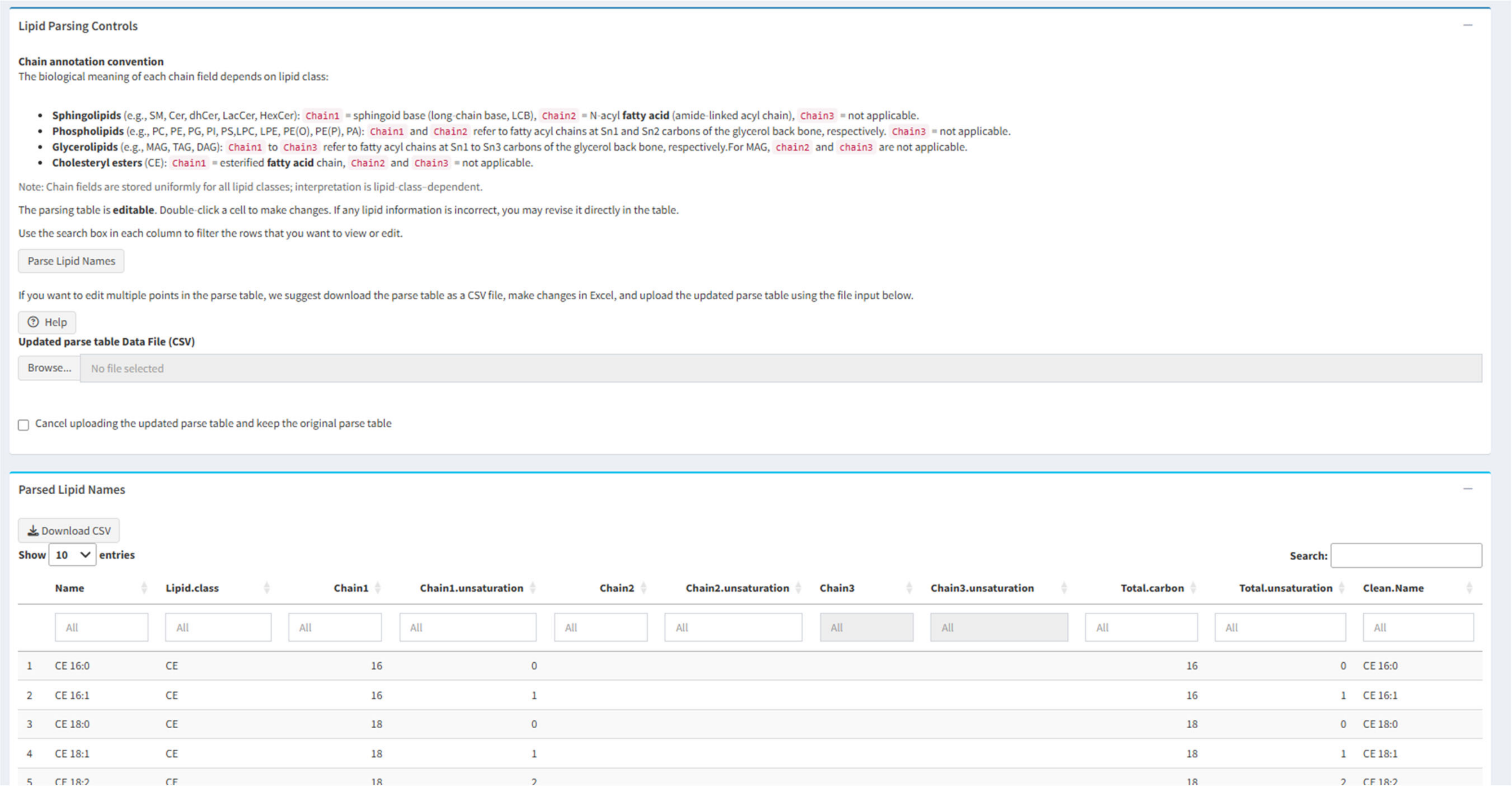

Figure S12: After clicking on “Parse Lipid Names”, further lipid characteristics for each lipid appear in adjacent columns which include lipid class, number of carbons in corresponding acyl chains (Chain1, Chain2, Chain3), number of double bonds in corresponding acyl chains (Chain1.unsatiration, Chain2.unsaturation, Chain3.unsaturation), total carbons in all acyl chains (Total.carbon), and total number of double bonds in all acyl chains (Total.unsaturation). If the allocation carbon numbers and double bonds is incorrect, users may click on the erroneous values and enter the correct numbers. Users can also download the table, perform bulk edits locally in Excel, and re-upload the updated lipid characteristics when extensive modifications are required. Users may search for the desired lipid in the database by typing it in the Search

area. After reviewing the allocated numbers of carbon atoms to acyl chains and the number of double bonds for each acyl chain and total lipid, click on blue “Next” to move forward.

Note: For Sphingolipids and ceramides, the “Chain.1” refers to sphingoid base, whereas “Chain.2” refers to N-Acyl fatty acids linked to carbon 2 of sphingoid base. For Phospholipids, “Chain.1” and “Chain.2” refers to fatty acyl chains at Sn1 and Sn2 carbons of the glycerol backbone, respectively. For Glycolipids, “Chain.1” to “Chain.3” refer to fatty acyl chains at Sn1 to Sn3 carbons of the glycerol backbone, respectively. For DAG “Chain.3”, and for MAG “Chain.2” and “Chain.3” are not applicable.

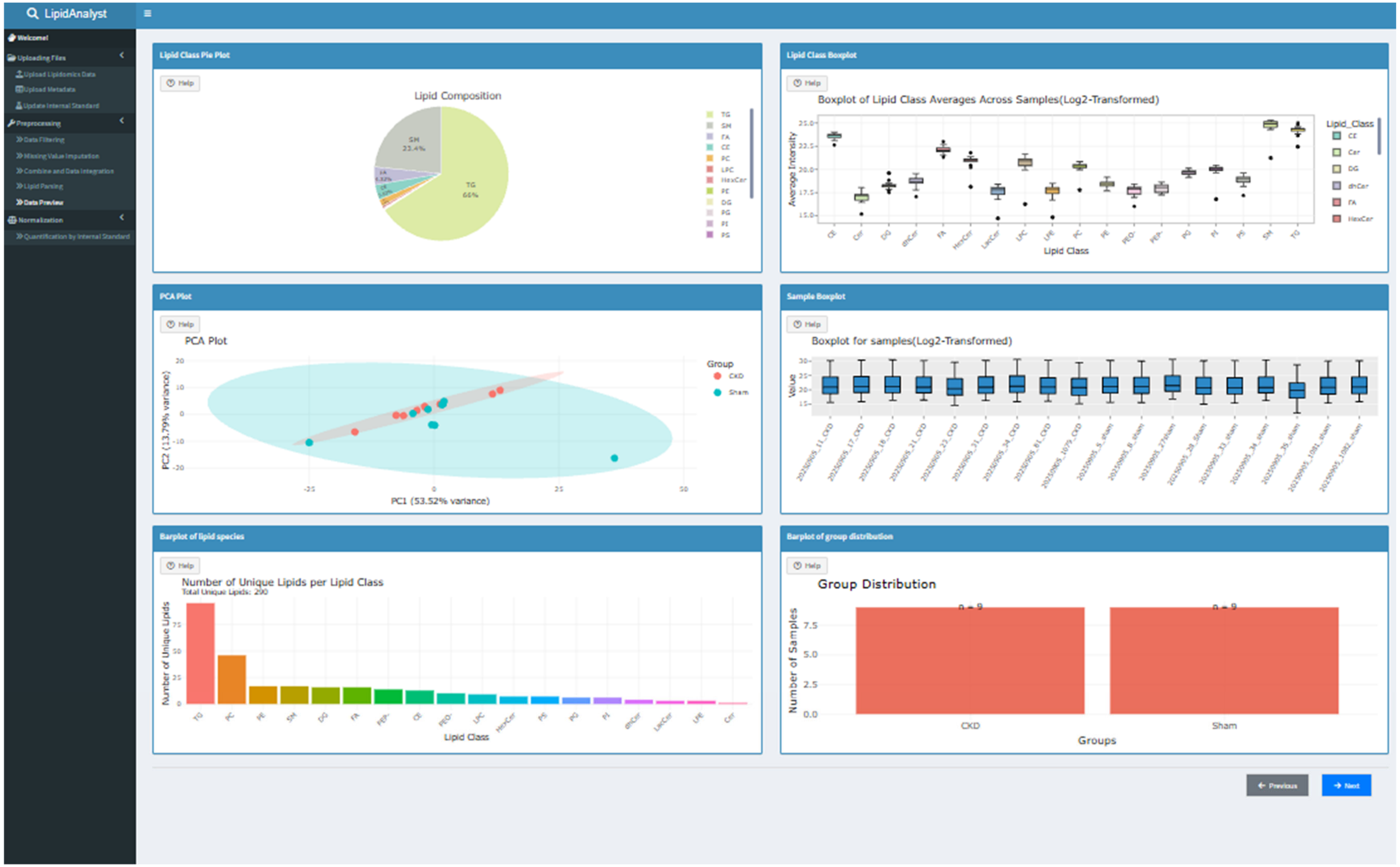

Figure S13: Data Preview: This section provides a global preview of data distribution through different graphs. Lipid class Pie Plot provides relative distribution of various lipid classes. PCA Plot shows separation of study groups using lipidomic data summarized in the top two principal component (PC) vectors using principal component analysis. Barplot of lipid species compares the absolute number of lipids within each class across study lipids. Lipid Class boxplot illustrates the

boxplot of lipids averaged intensities across samples (Log2 transformed). Sample Boxplot shows the boxplot of average lipid intensities for each sample. Barplot of group distribution shows the sample size per study group. Clicking on “Help” icon in each panel provides additional information. Click on “Next” to move forward.

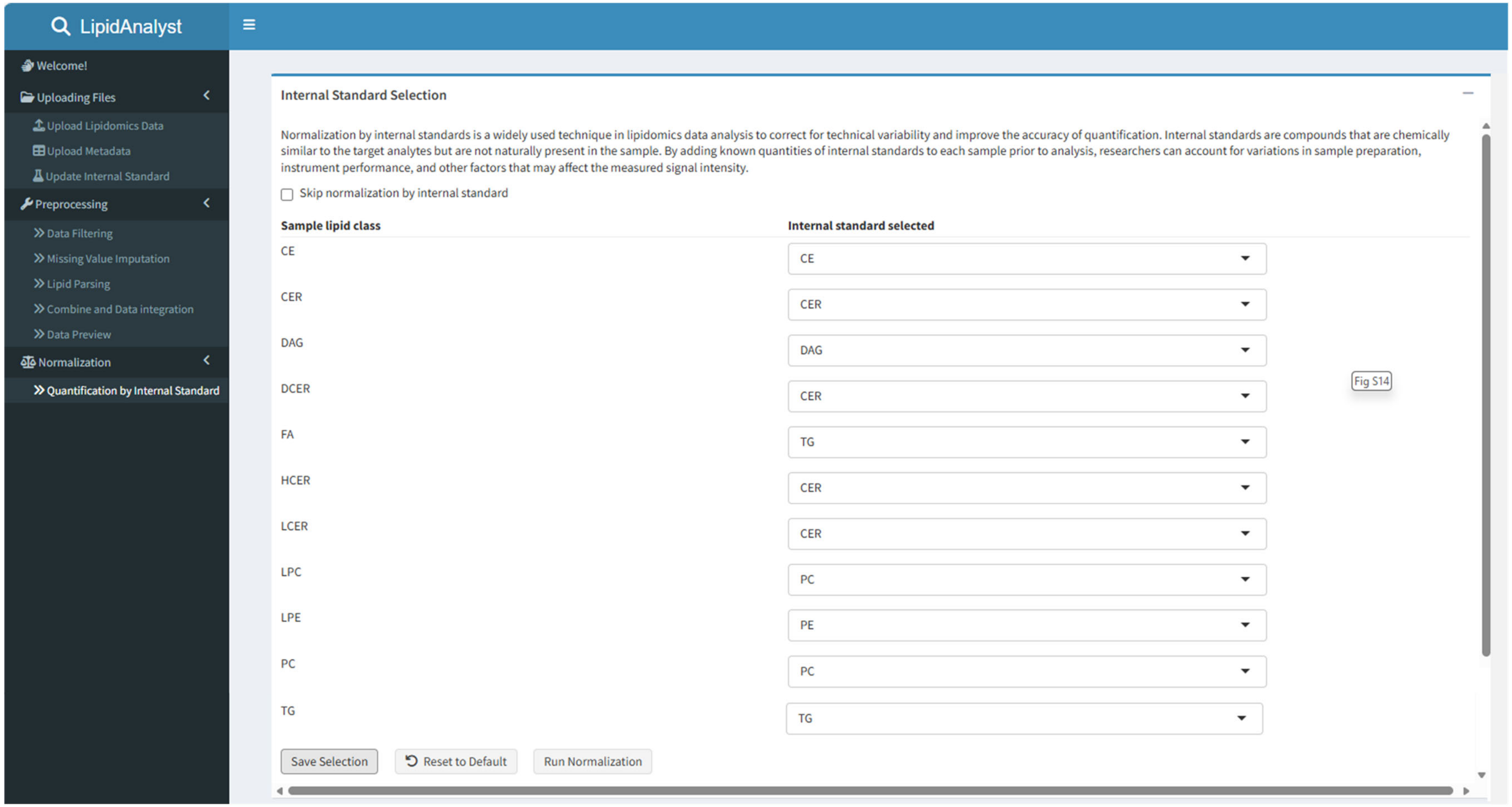

Figure S14: Quantification by Internal Standard: Users have the option to select the internal standard for normalization of each lipid class. This can be done by choosing the intended internal standard for each lipid class from the “Internal standard selected” box. If the dataset is already normalized by internal standard or internal standard normalization does not apply, this section can be skipped by clicking on the “Skip normalization by internal standard” box. Once proper internal standards are selected click on “Save Selection”, and then on “Run Normalization”. Upon normalization, the normalized data can be viewed under “Normalized Data Preview” (Figure S15).

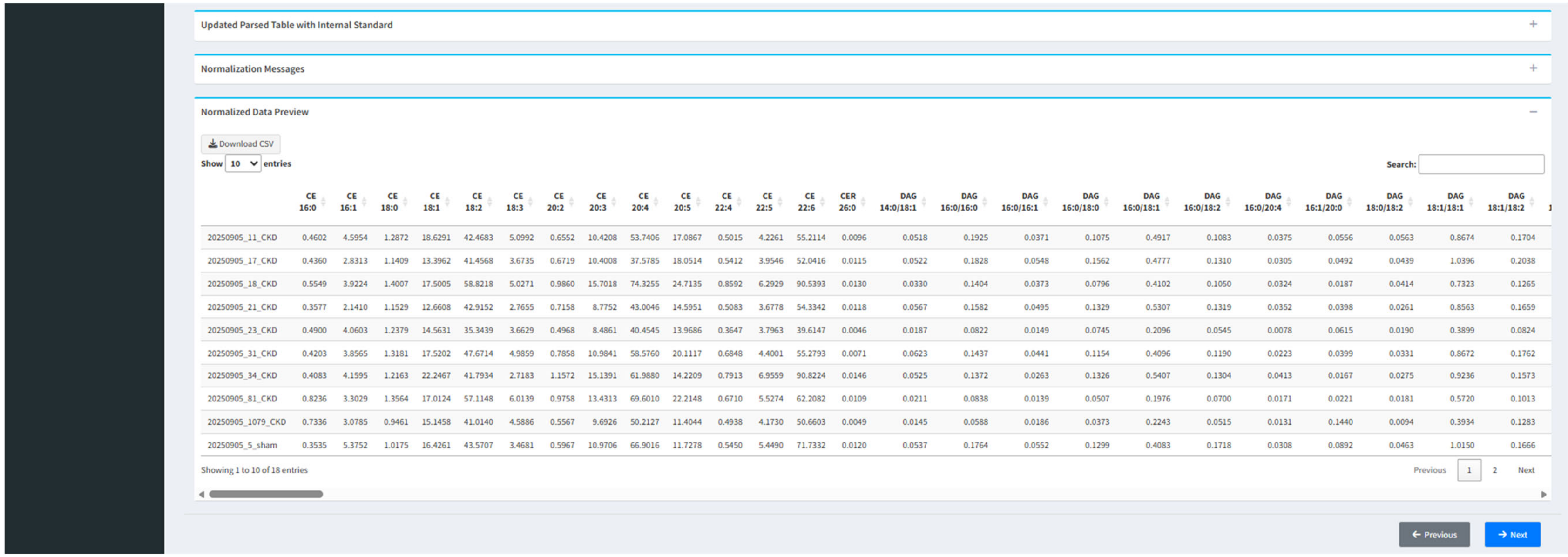

	CE 16:0	CE 16:1	CE 18:0	CE 18:1	CE 18:2	CE 18:3	CE 20:2	CE 20:3	CE 20:4	CE 20:5	CE 22:4	CE 22:5	CE 22:6
20250905_11_CKD	0.4602	4.5954	1.2872	18.6291	42.4683	5.0992	0.6552	10.4208	53.7406	17.0867	0.5015	4.2261	55.2114
20250905_17_CKD	0.4360	2.8313	1.1409	13.3962	41.4568	3.6735	0.6719	10.4008	37.5785	18.0514	0.5412	3.9546	52.0416
20250905_18_CKD	0.5549	3.9224	1.4007	17.5005	58.8218	5.0271	0.9860	15.7018	74.3255	24.7135	0.8592	6.2929	90.5393
20250905_21_CKD	0.3577	2.1410	1.1529	12.6608	42.9152	2.7655	0.7158	8.7752	43.0046	14.5951	0.5083	3.6778	54.3342
20250905_23_CKD	0.4900	4.0603	1.2379	14.5631	35.3439	3.6629	0.4968	8.4861	40.4545	13.9686	0.3647	3.7963	39.6147
20250905_31_CKD	0.4203	3.8565	1.3181	17.5202	47.6714	4.9859	0.7858	10.9841	58.5760	20.1117	0.6848	4.4001	55.2793
20250905_34_CKD	0.4083	4.1595	1.2163	22.2467	41.7934	2.7183	1.1572	15.1391	61.9880	14.2209	0.7913	6.9559	90.8224
20250905_81_CKD	0.8236	3.3029	1.3564	17.0124	57.1148	6.0139	0.9758	13.4313	69.6010	22.2148	0.6710	5.5274	62.2082
20250905_1079_CKD	0.7336	3.0785	0.9461	15.1458	41.0140	4.5886	0.5567	9.6926	50.2127	11.4044	0.4938	4.1730	50.6603
20250905_5_sham	0.3535	5.3752	1.0175	16.4261	43.5707	3.4681	0.5967	10.9706	66.9016	11.7278	0.5450	5.4490	71.7332

	CER 26:0	DAG 14:0/18:1	DAG 16:0/16:0	DAG 16:0/16:1	DAG 16:0/18:0	DAG 16:0/18:1	DAG 16:0/18:2	DAG 16:0/20:4	DAG 16:1/20:0	DAG 18:0/18:2	DAG 18:1/18:1	DAG 18:1/18:2
20250905_11_CKD	0.0096	0.0518	0.1925	0.0371	0.1075	0.4917	0.1083	0.0375	0.0556	0.0563	0.8674	0.1704
20250905_17_CKD	0.0115	0.0522	0.1828	0.0548	0.1562	0.4777	0.1310	0.0305	0.0492	0.0439	1.0396	0.2038
20250905_18_CKD	0.0130	0.0330	0.1404	0.0373	0.0796	0.4102	0.1050	0.0324	0.0187	0.0414	0.7323	0.1265
20250905_21_CKD	0.0118	0.0567	0.1582	0.0495	0.1329	0.5307	0.1319	0.0352	0.0398	0.0261	0.8563	0.1659
20250905_23_CKD	0.0046	0.0187	0.0822	0.0149	0.0745	0.2096	0.0545	0.0078	0.0615	0.0190	0.3899	0.0824
20250905_31_CKD	0.0071	0.0623	0.1437	0.0441	0.1154	0.4096	0.1190	0.0223	0.0399	0.0331	0.8672	0.1762
20250905_34_CKD	0.0146	0.0525	0.1372	0.0263	0.1326	0.5407	0.1304	0.0413	0.0167	0.0275	0.9236	0.1573
20250905_81_CKD	0.0109	0.0211	0.0838	0.0139	0.0507	0.1976	0.0700	0.0171	0.0221	0.0181	0.5720	0.1013
20250905_1079_CKD	0.0049	0.0145	0.0588	0.0186	0.0373	0.2243	0.0515	0.0131	0.1440	0.0094	0.3934	0.1283
20250905_5_sham	0.0120	0.0537	0.1764	0.0552	0.1299	0.4083	0.1718	0.0308	0.0892	0.0463	1.0150	0.1666

Figure S15: Upon normalization by internal standards, the normalized data can be viewed under “Normalized Data Preview”. To verify which lipid class is normalized by which internal standard, expand the section of “Updated Parsed Table with Internal Standard”. To review the normalization codes, expand the section of “Normalization Messages”. Click on blue “Next” to move forward.

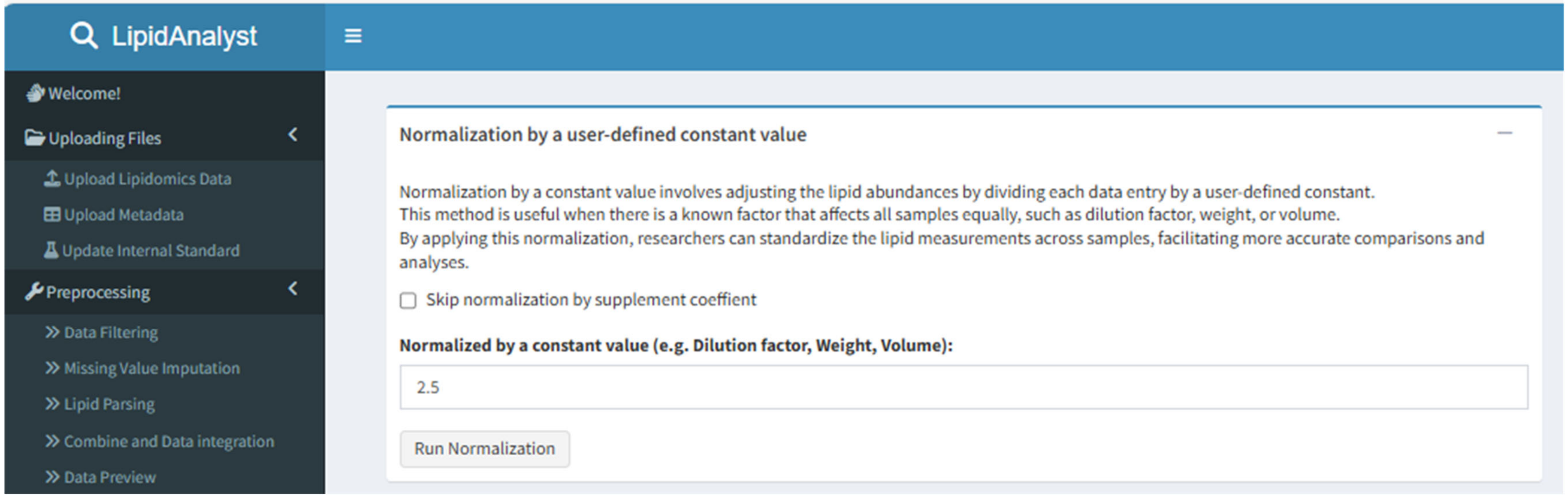

Figure S16A: Quantification by User Defined Factor: Users have the option to further normalize the data by a constant value when there is a known factor that affects all samples equally, such as dilution factor, weight, or volume. The appropriate value can be entered in the “Normalized by a constant value” tab. Users may skip this section by clicking on the “Skip normalization by supplement coefficient” box.

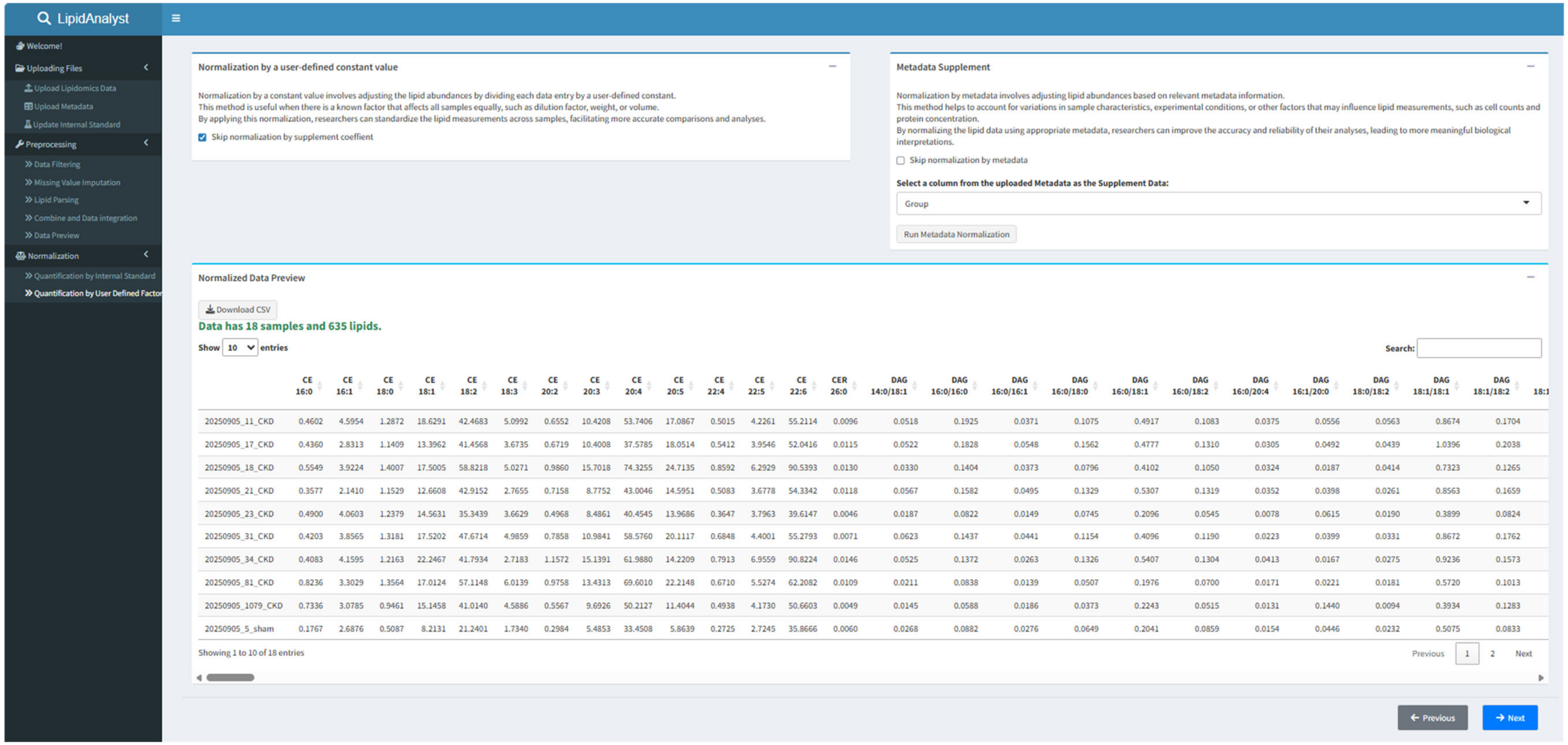

Figure S16B: Quantification by User Defined Factor: Similarly, users may further normalize the data by metadata. The metadata can be selected from the “Select a column from the uploaded Metadata as the Supplement Data” tab. Users may skip this section by clicking on the “Skip normalization by metadata” box. Click on blue “Next” to move forward.

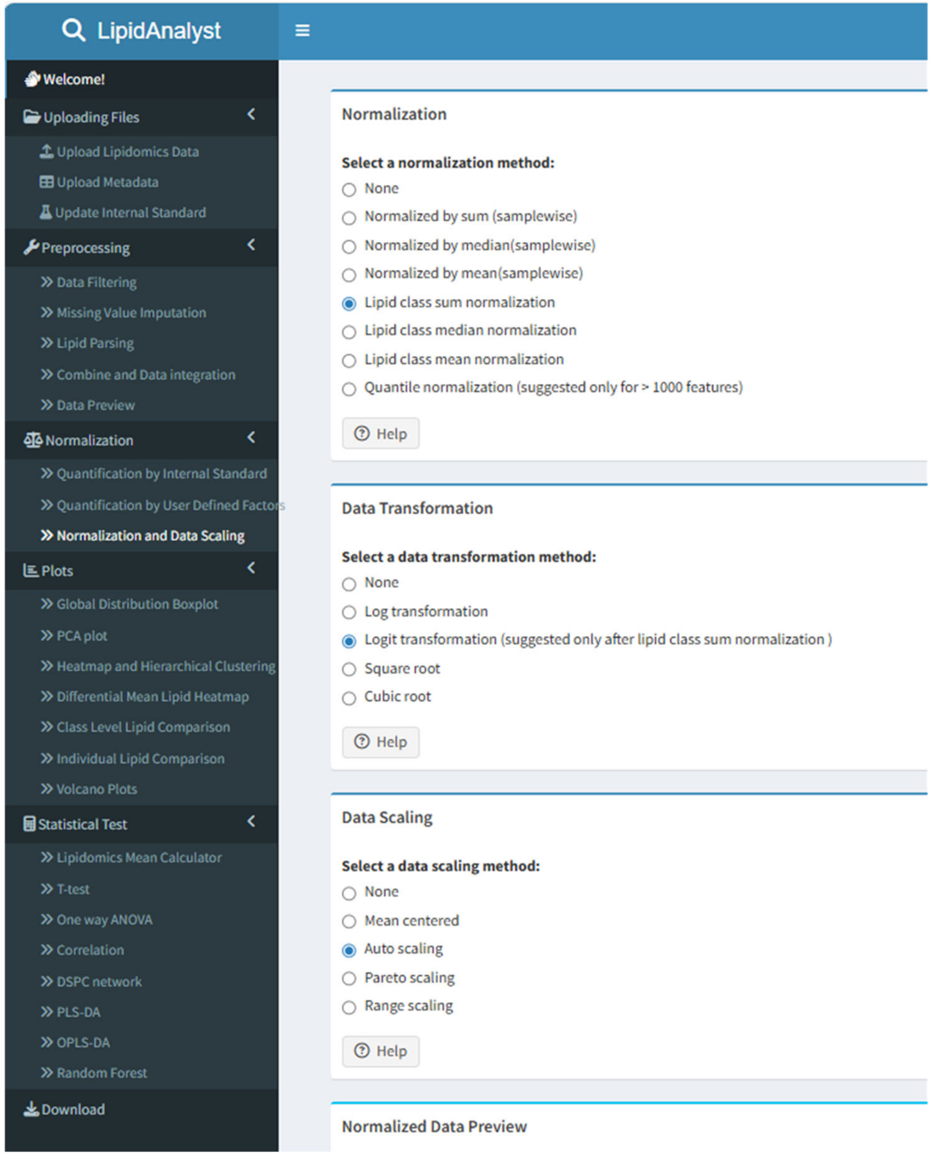

Figure S17: Further normalization can be pursued. The normalization methods include:

- Normalized by sum (samplewise) – Divides each feature by the total abundance in the sample.
- Normalized by median (samplewise) – Divides each feature by the sample median.
- Normalized by mean (samplewise) – Divides each feature by the sample mean.
- Lipid class sum normalization – Normalizes within each lipid class based on the total abundance of that class. If there is only one lipid within the lipid class, the original value of the lipid would be kept.
- Lipid class median normalization – Uses the median of each lipid class for normalization.
- Lipid class mean normalization – Uses the mean of each lipid class for normalization.
- Quantile normalization – Aligns the distribution of all samples to make them comparable.

Data Transformation can be pursued. The transformation methods include:

- Log transformation: Reduces skewness and stabilizes variance. Log base is selectable.
- Logit transformation: Suggested only after lipid class sum normalization. Ideal for data bounded between 0 and 1.
- Square root: An alternative to log transformation.
- Cubic root: Reduces the impact of extreme values.

At the end, data scaling can be pursed. The data scaling methods include:

Mean centering – Centers variables around zero.
Auto scaling (Z-score) – Mean-centers and scales by standard deviation.
Pareto scaling – Scales by the square root of the standard deviation.
Range scaling – Scales data to a fixed range (e.g., 0–1).

Users may select “None” on data normalization, transformation, or scaling methods if none of the methods are applicable or the method not indicated. After selecting the proper method, click on “Run Normalization Plan” tab to implement it. Once normalization plan is implemented, the normalized data can be viewed under “Normalized Data Preview”. Click on blue “Next” to move forward.

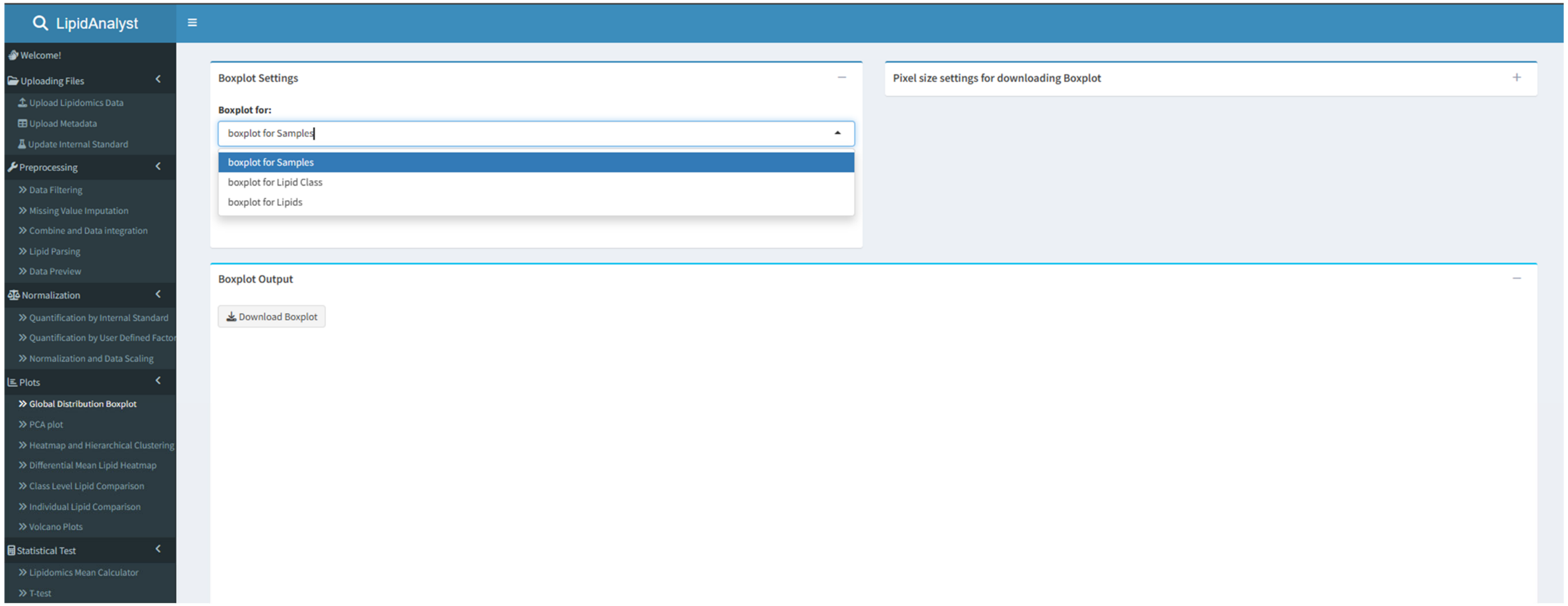

Figure S18: Global distribution boxplot: Boxplots can be generated for sample, lipid class, and individual lipids. The generated plots can be downloaded by clicking on “Download Boxplot”. Click the plus sign in the pixel size setting for downloading Boxplot for additional edits towards width, height and DPI.

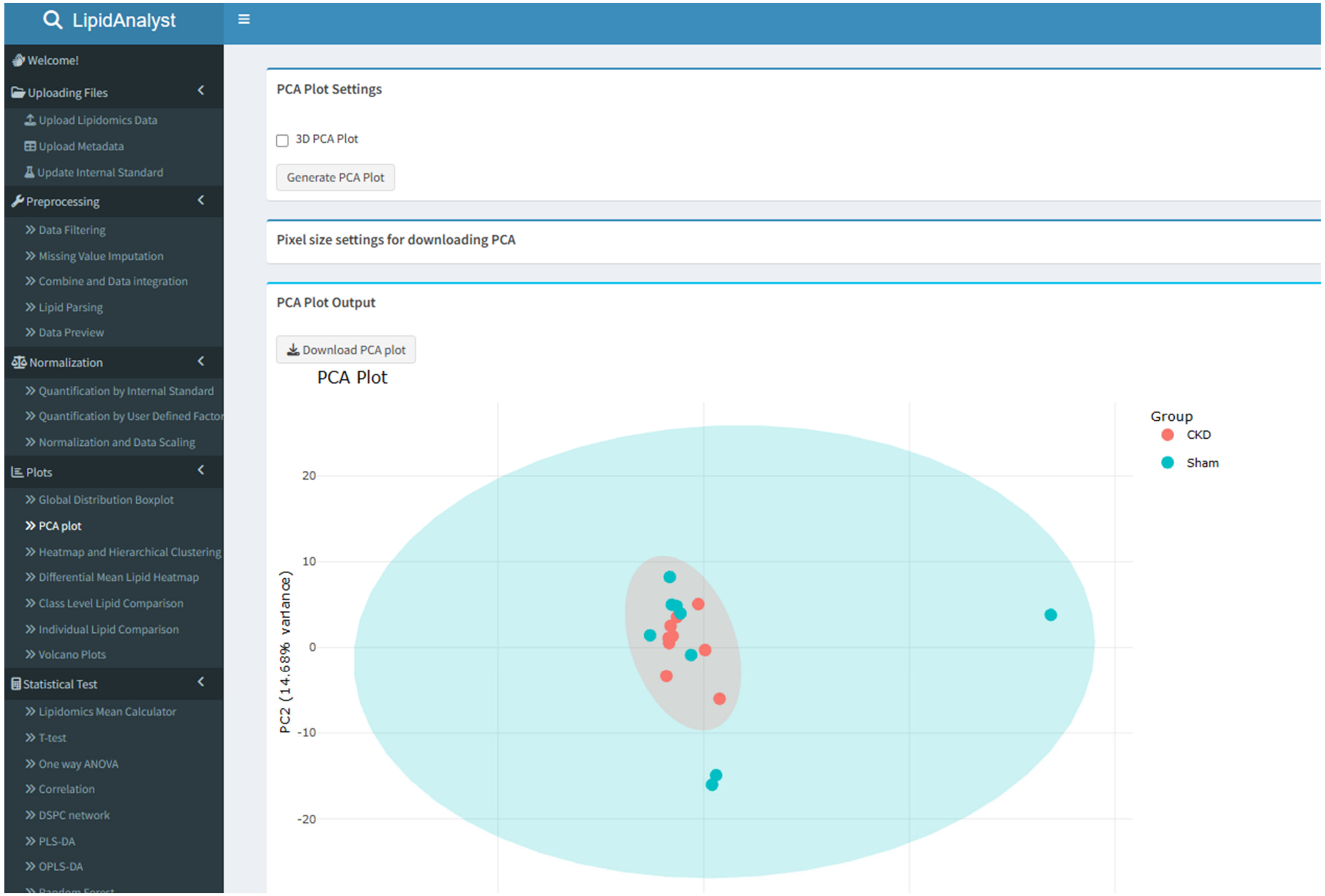

Figure S19A: PCA plot: Allows exploring global variation and sample clustering using a 2D or 3D PCA plots. Pixel size settings are adjustable. Here a 2D PCA plot is illustrated.

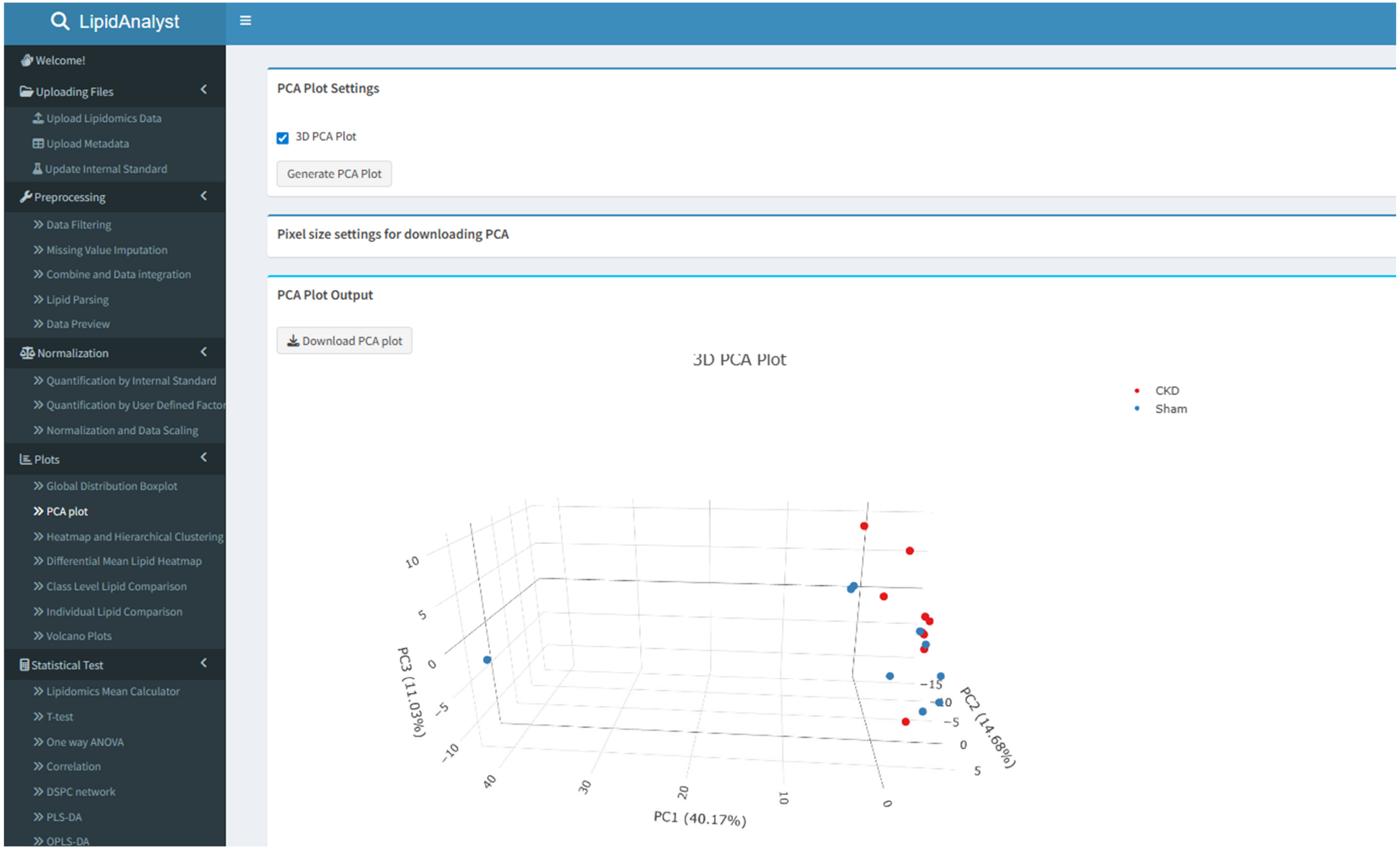

Figure S19B: 3D PCA plot can be generated by clicking on “3D PCA Plot” and then “Generate PCA Plot” tabs.

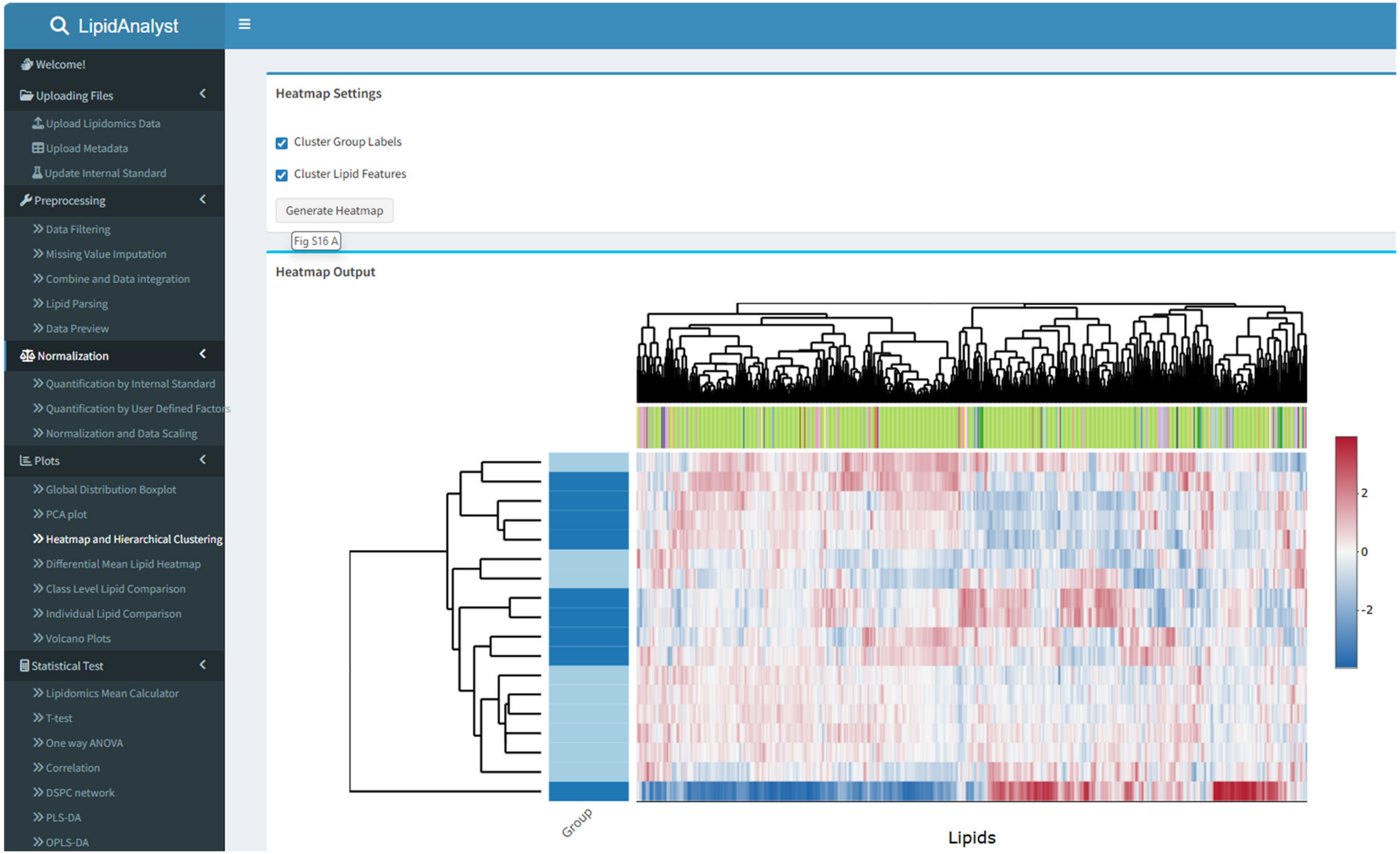

Figure S20: Heatmap and Hierarchical Clustering is used to reveal patterns and relationships in complex datasets that may not be immediately apparent through other forms of analysis. This method groups similar rows and columns of the matrix together based on a chosen similarity measure, such as Euclidean distance or Pearson correlation. To avoid clustering in rows or columns deselect “Cluster Rows” or “Cluster Columns” tabs as appropriate.

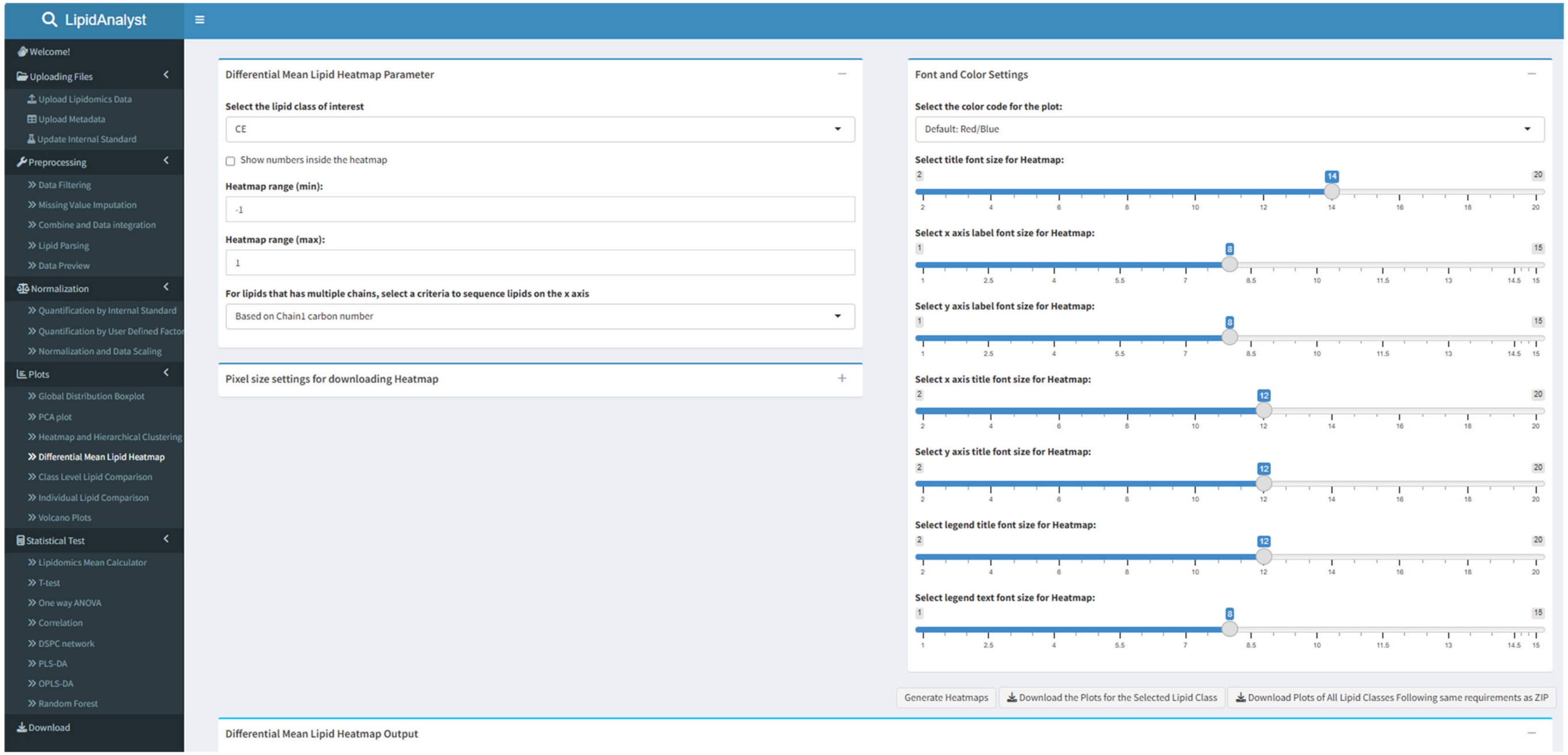

Figure S21: Differential Mean Lipid Heatmap (DMLH) provides a graphical view of the mean value of various lipids of a lipid class by their acyl chain carbon numbers shown in X-axis and the number of acyl chain double bonds shown in Y-axis in study subgroups. First select the lipid class of interest. Users have the option to modify the range of heatmap color code by adjusting the minimum and maximum heatmap range. For lipids with multiple acyl chains, the users can sort the X-axis by the number of carbons on chain1, chain2, or total number of all chains. Colors can be changed from a number of options available under "Select the color code for the plot" tab. Font size of title, x-axis label, y-axis label, x-axis title, and y-axis title can be adjusted according to size of the heatmap for optimal visualization. After selecting the intended lipid class and proper settings click on the "Generate Heatmaps" tab. Each set of generated heatmaps or all heatmaps for all lipid classes can be downloaded. Once the heatmaps are generated, they can be viewed under the "Differential Mean Lipid Heatmap Output".

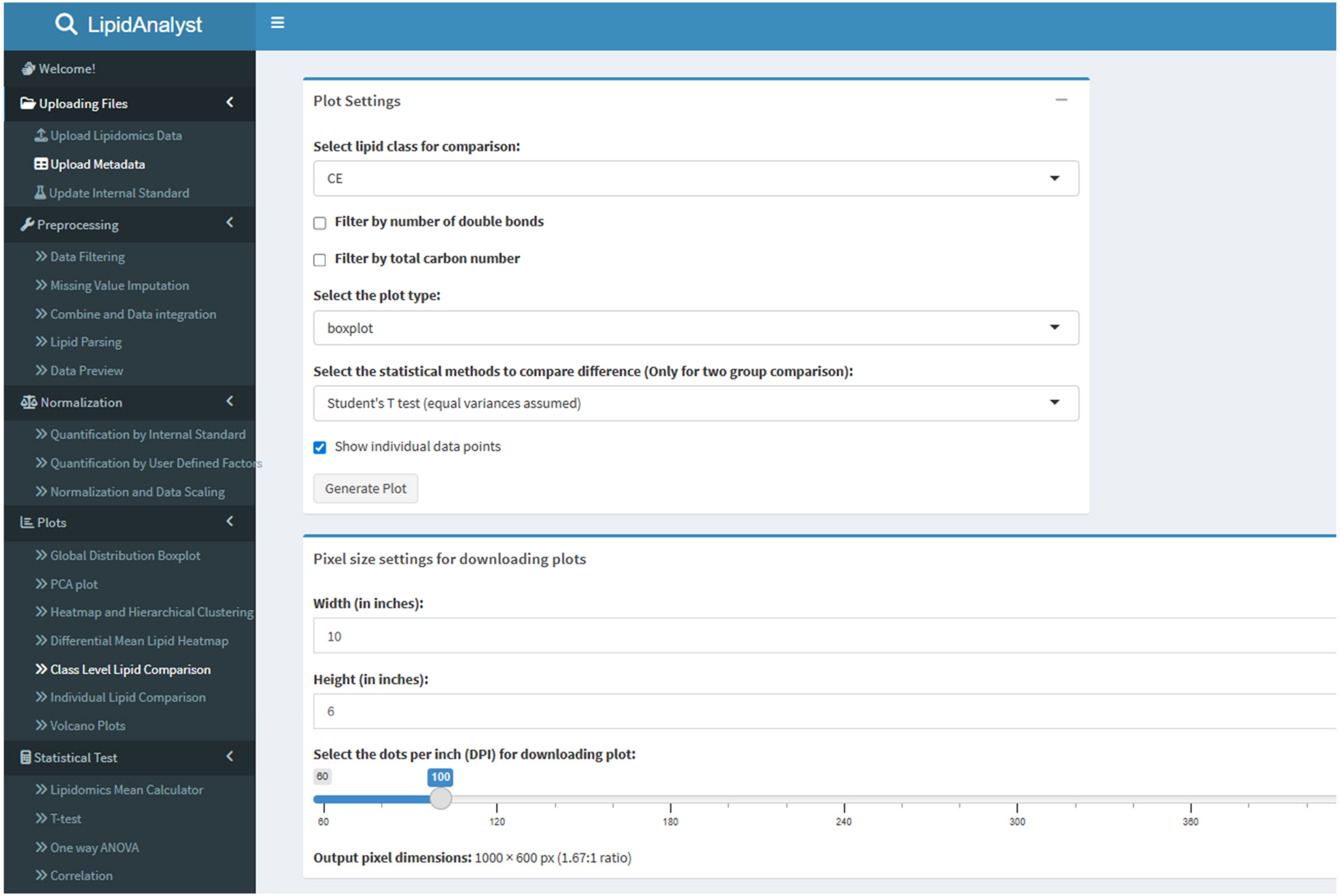
LipidAnalyst
Welcome!
Uploading Files
Upload Lipidomics Data
Upload Metadata
Update Internal Standard
Preprocessing
Data Filtering
Missing Value Imputation
Combine and Data integration
Lipid Parsing
Data Preview
Normalization
Quantification by Internal Standard
Quantification by User Defined Factors
Normalization and Data Scaling
Plots
Global Distribution Boxplot
PCA plot
Heatmap and Hierarchical Clustering
Differential Mean Lipid Heatmap
Class Level Lipid Comparison
Individual Lipid Comparison
Volcano Plots
Statistical Test
Lipidomics Mean Calculator
T-test
One way ANOVA
Correlation
Plot Settings
Select lipid class for comparison:
CE
Filter by number of double bonds
Filter by total carbon number
Select the plot type:
boxplot
Select the statistical methods to compare difference (Only for two group comparison):
Student's T test (equal variances assumed)
Show individual data points
Generate Plot
Pixel size settings for downloading plots
Width (in inches):
10
Height (in inches):
6
Select the dots per inch (DPI) for downloading plot:
60
100
60
120
180
240
300
360
Output pixel dimensions: 1000 × 600 px (1.67:1 ratio)

Figure S22: Class Level Lipid Comparison provides visualization of lipids by study groups using boxplot or violin plots. Select the lipid class for comparison. If filters are not checked a pulled distribution of all lipids within the lipid class will be compared by study groups. If comparing the distribution of a range of lipids with certain number of double bonds or carbon numbers is desired, the filters can be applied to generate the corresponding plots. Two types of available plots are boxplots and violin plots. Statistical methods to compare mean differences including Student's T test (equal variances assumed), Welch's test (unequal variances allowed), and Wilcoxon test are available options. Once appropriate settings are selected click on "Generate the Plot". Pixel size settings are adjustable. The plots can be viewed under the "Plot Output".

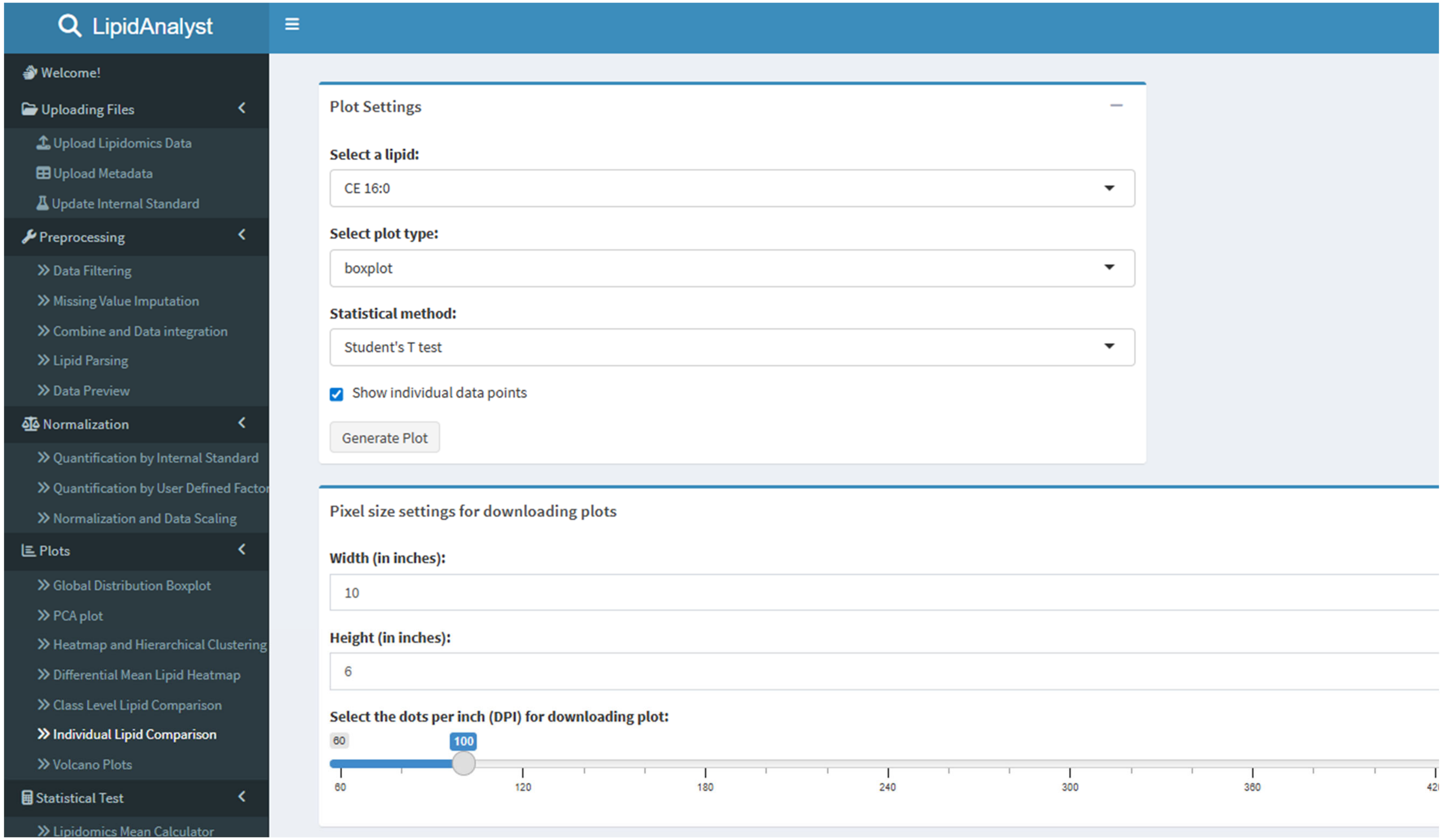

Figure S23: Individual Lipid Comparison provides the graphical view of each individual lipid by study groups using boxplot or violin plot. Plot type can be selected from the “Select plot type” box. Statistical methods to compare mean differences including Student’s T test (equal variances assumed), Welch’s test (unequal variances allowed), and Wilcoxon test are available options. Once appropriate settings are selected click on “Generate Plot”. Pixel size settings are adjustable. The plots can be viewed under the “Plot Output”.

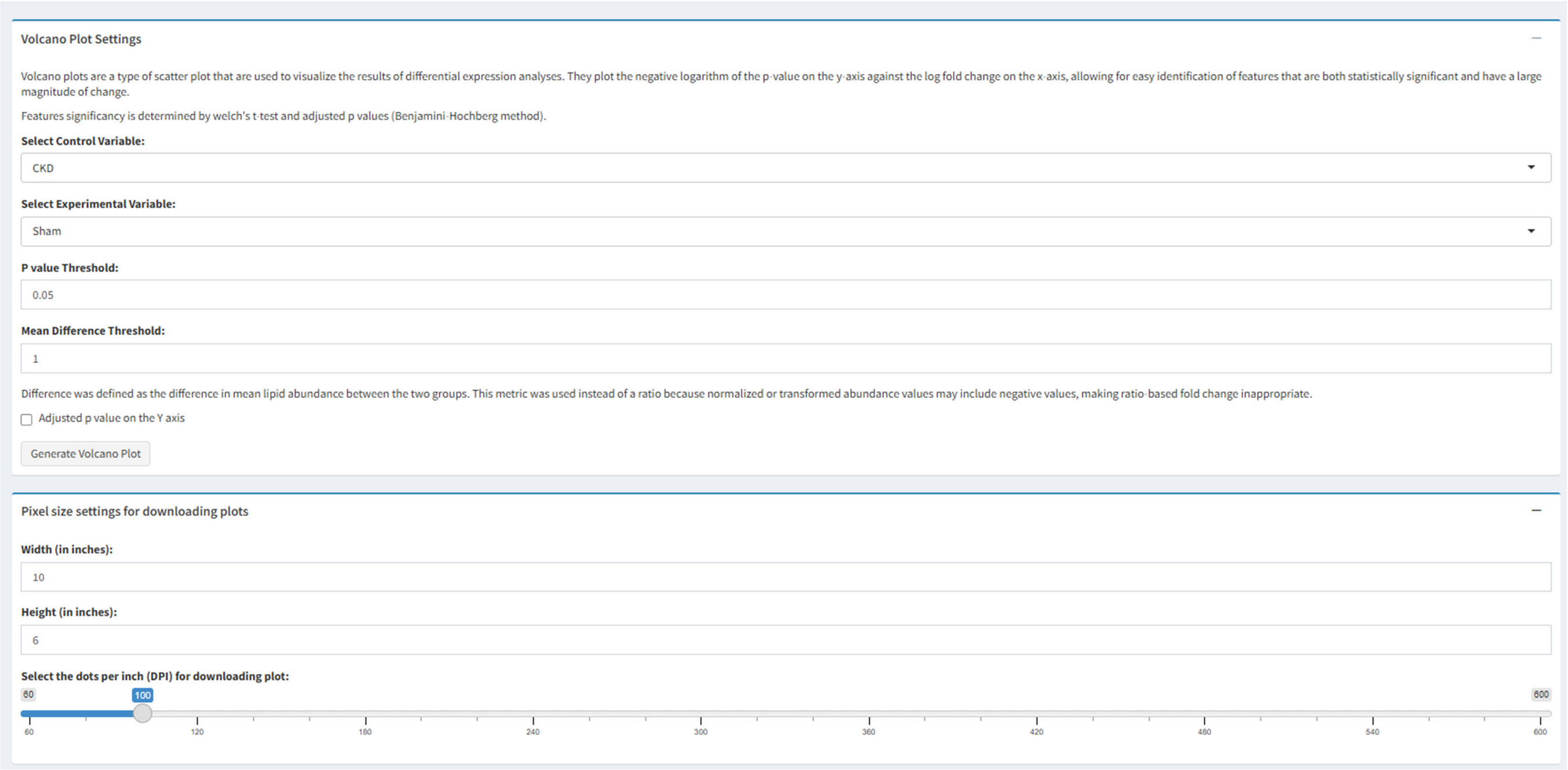

Figure S24: Volcano plots can be generated by selecting the Grouping variable, the Comparison Variable, and choosing the desired p-value and fold change thresholds. To adjust the p values for multiplicity, click on “Adjust p value on the Y axis” is an option. After selecting the proper settings, click on “Generate Volcano Plot”. Pixel size settings are adjustable. The plot can be viewed under “Volcano Plot Output”.

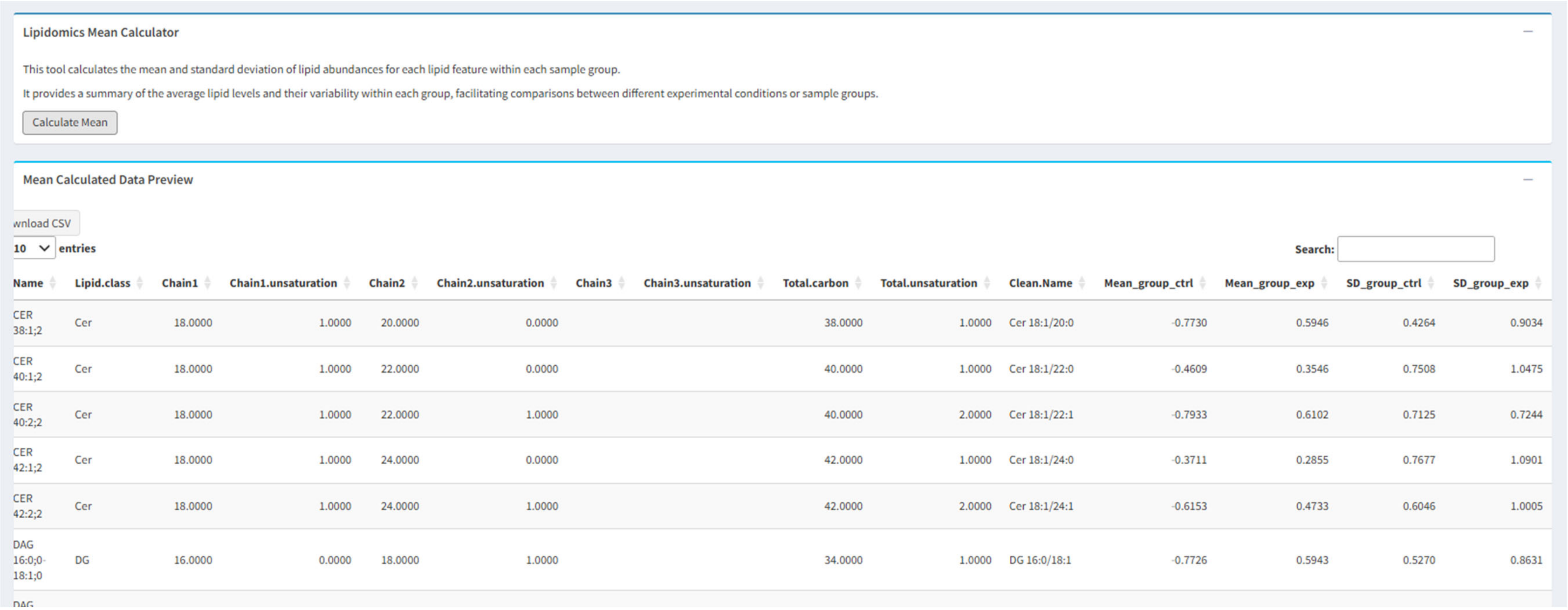

Figure S25: Lipidomics Mean Calculator reports normalized mean value of each lipid by study groups. To obtain the means, click on “Calculate Mean” tab. The output can be viewed under “Mean Calculated Data Preview”. Number of reported lipids (entries) per page can be adjusted as appropriate. The report can be downloaded in a csv file by clicking on the “Download csv” tab.

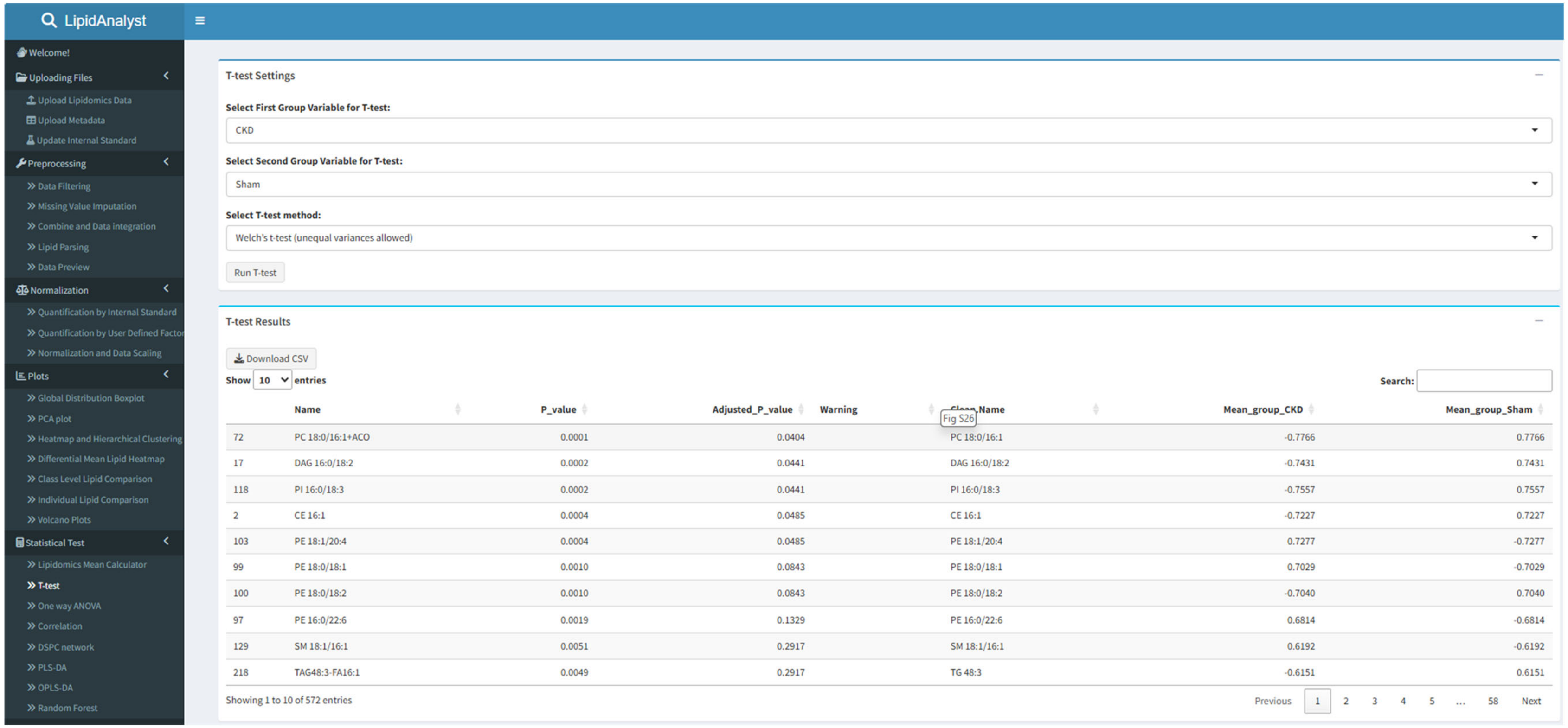

Figure S26A: T-test provides the p values and adjusted p values for the mean differences by study groups. Once the grouping variables are selected, click on “Run T test” tab. The output can be viewed under “T-test Results”. Number of reported lipids (entries) per page can be adjusted as appropriate. The report can be downloaded in a csv file by clicking on the “Download csv” tab.

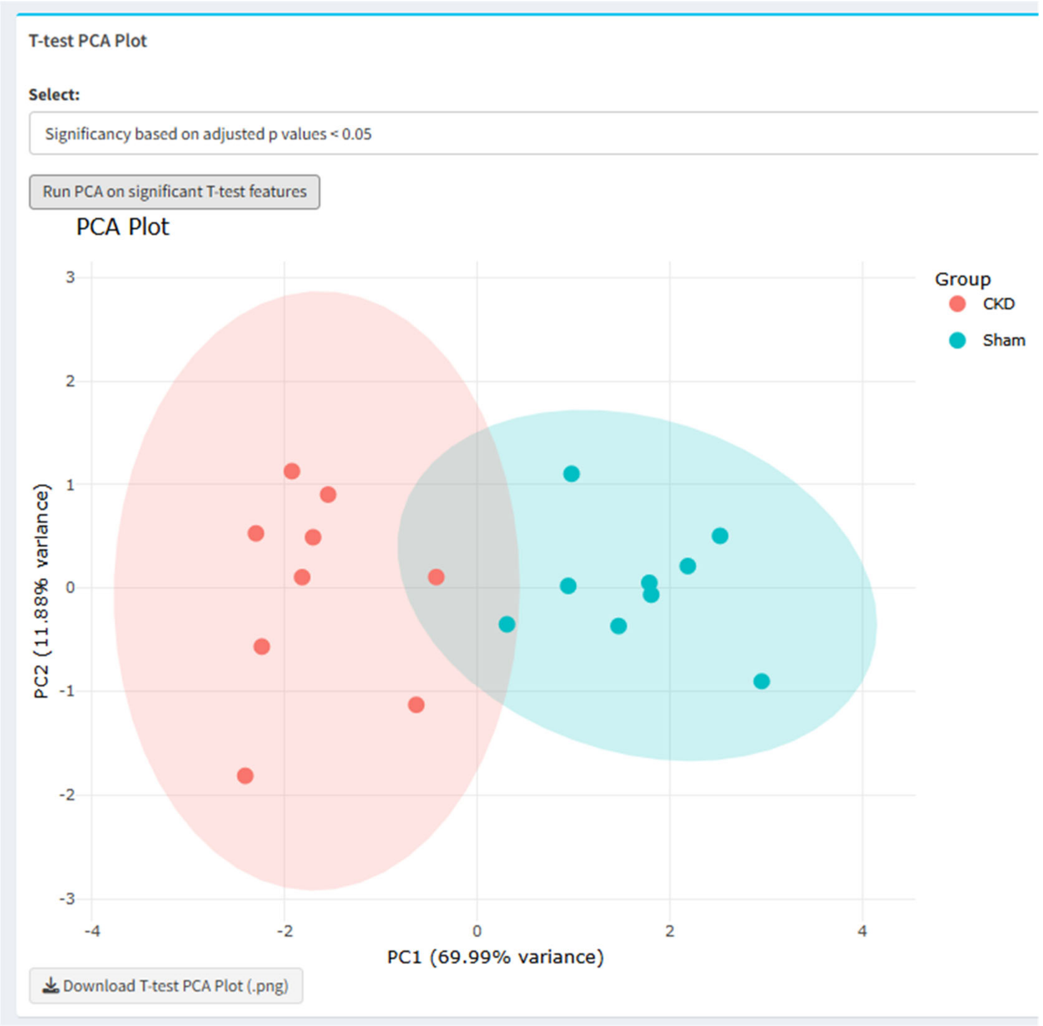

Figure S26B: T-test PCA plot is constructed using significant lipids from the T-test. Users may define significance based on adjusted p values or nominal p values. Upon clicking “Run PCA on Significant T-test Features”, the PCA plot is generated. The resulting plot can be exported as a PNG file via “Download T-test PCA Plot (.png)”.

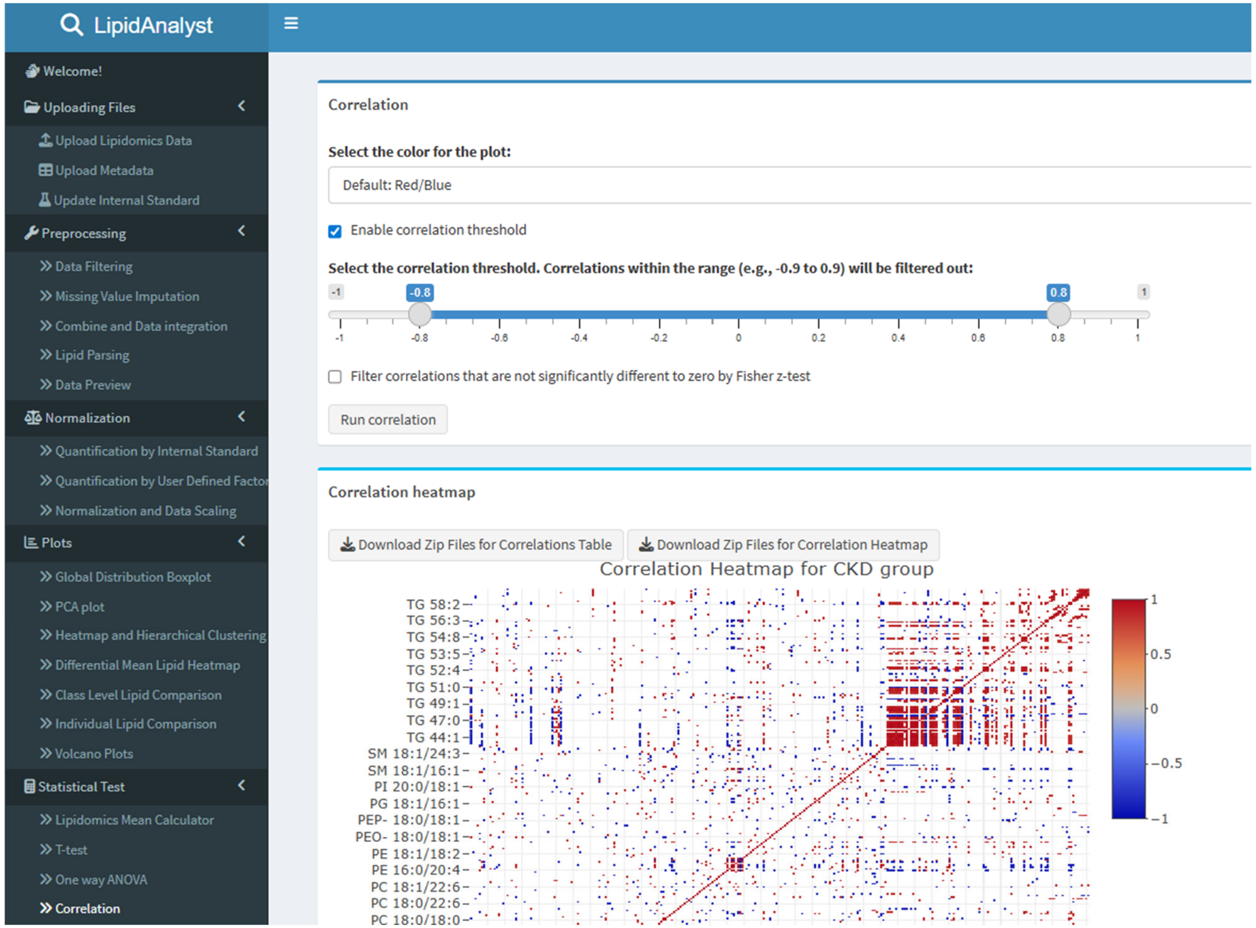

Figure S27A: Correlation plot provides a bivariate correlation heatmap across lipids by study groups. The color for the plots can be changed from the list of available options. If a correlation threshold is not selected, all the lipids will be implemented in the generation of the graphs. If the threshold is selected, the users may define a range of correlations that will be filtered out in the graph generation. Similarly, the correlations that are not significantly different from zero can be filtered out by selecting the correspond box above. Once proper settings are selected, click on "Run Correlation" tab. The heatmaps can be viewed under Correlation heatmap. Correlations tables and correlation heatmaps are ready to download once users hit download buttons.

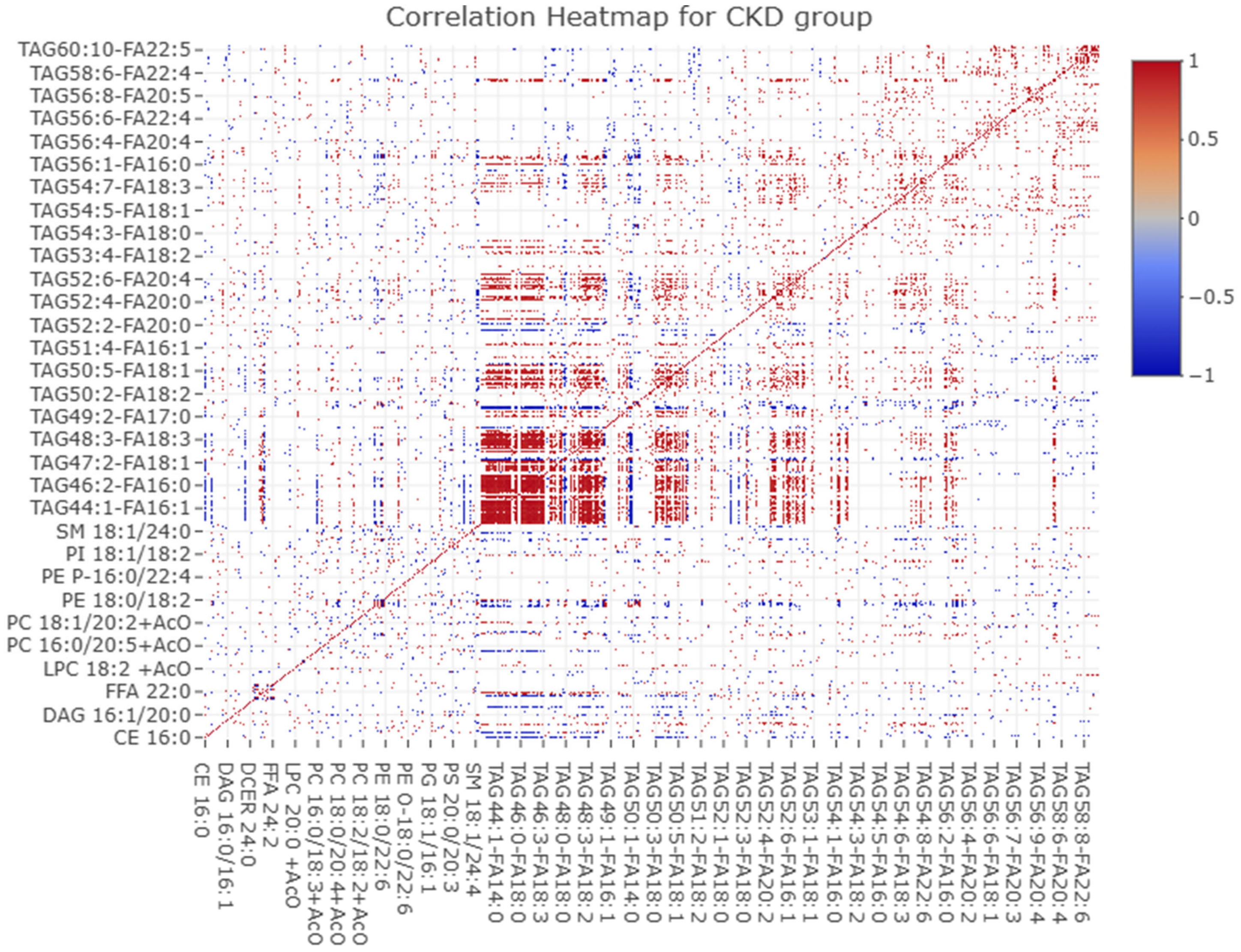

Figure S27B: Bivariate correlation heatmap for CKD group

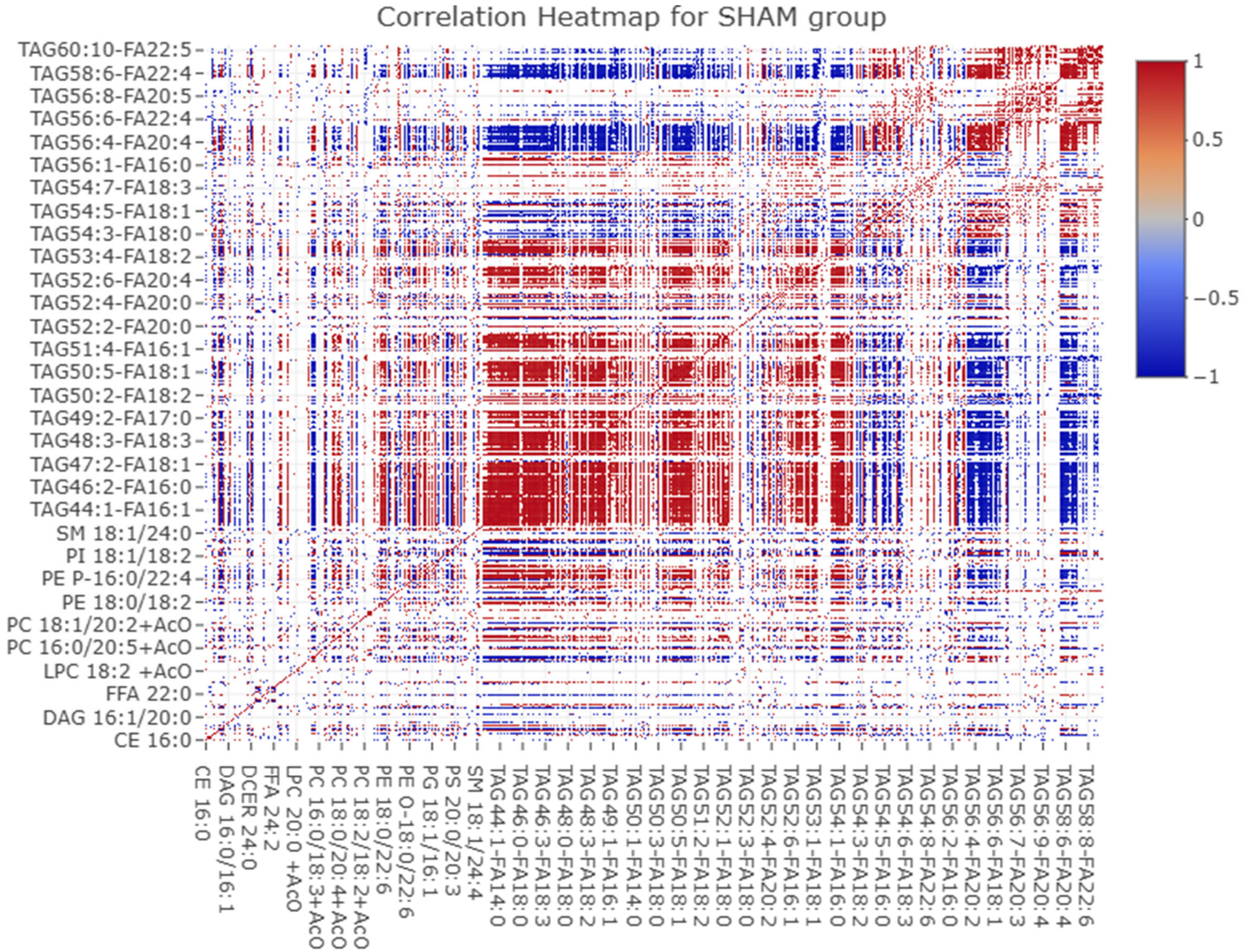

Figure S27C: Bivariate correlation heatmap for Sham group

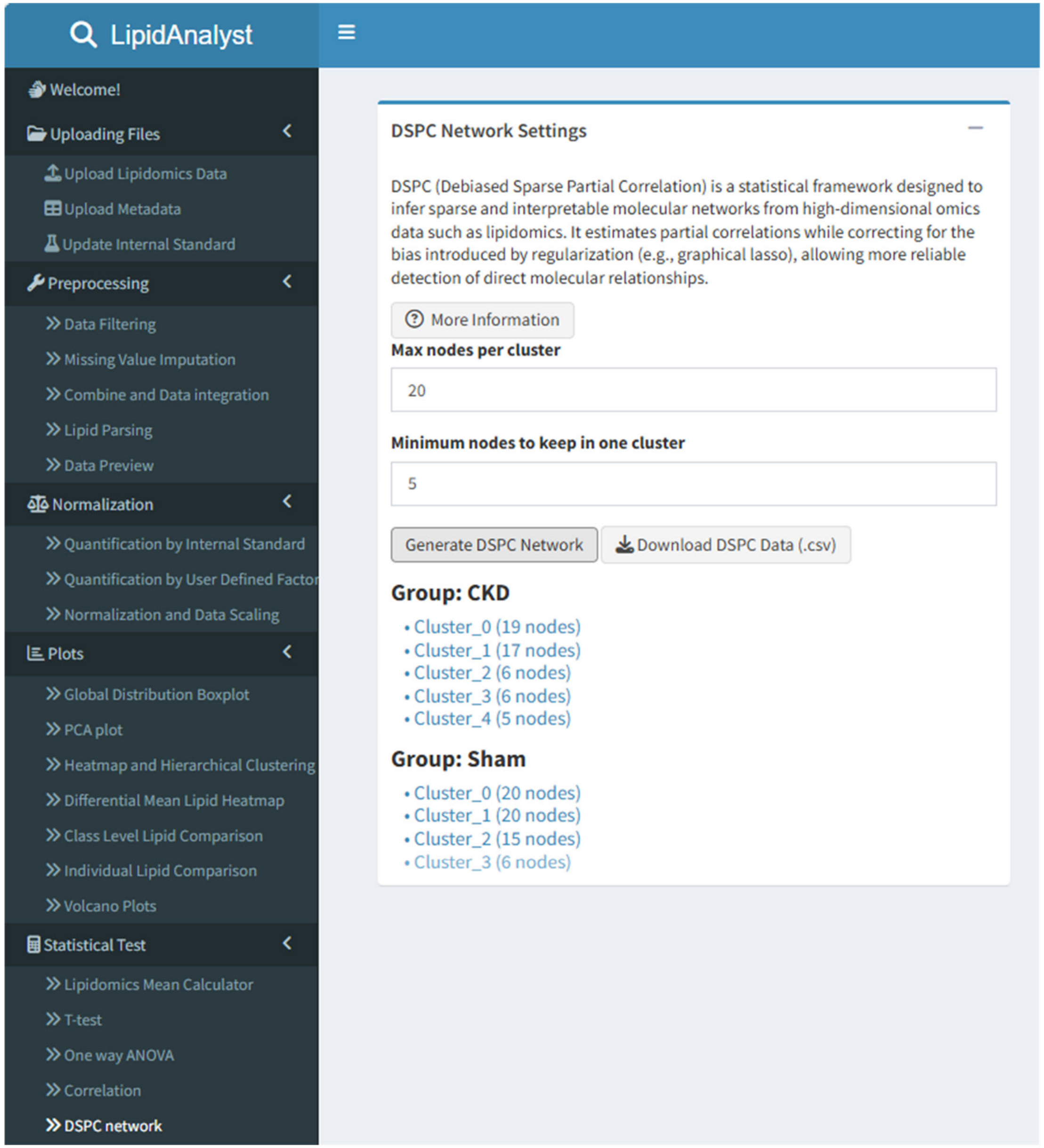

Figure S28: DSPC network: DSPC (Debiased Sparse Partial Correlation) provides the graphical view of sparse molecular networks by estimating partial correlations and correcting by graphical lasso. After defining the thresholds for maximum number of nodes per cluster and minimum number of nodes required generate one cluster, users may generate the network by clicking on “Generate DSPC Network”. Once the clusters are generated, they can be visualized under “DSPC Network Output” by clicking on each cluster.

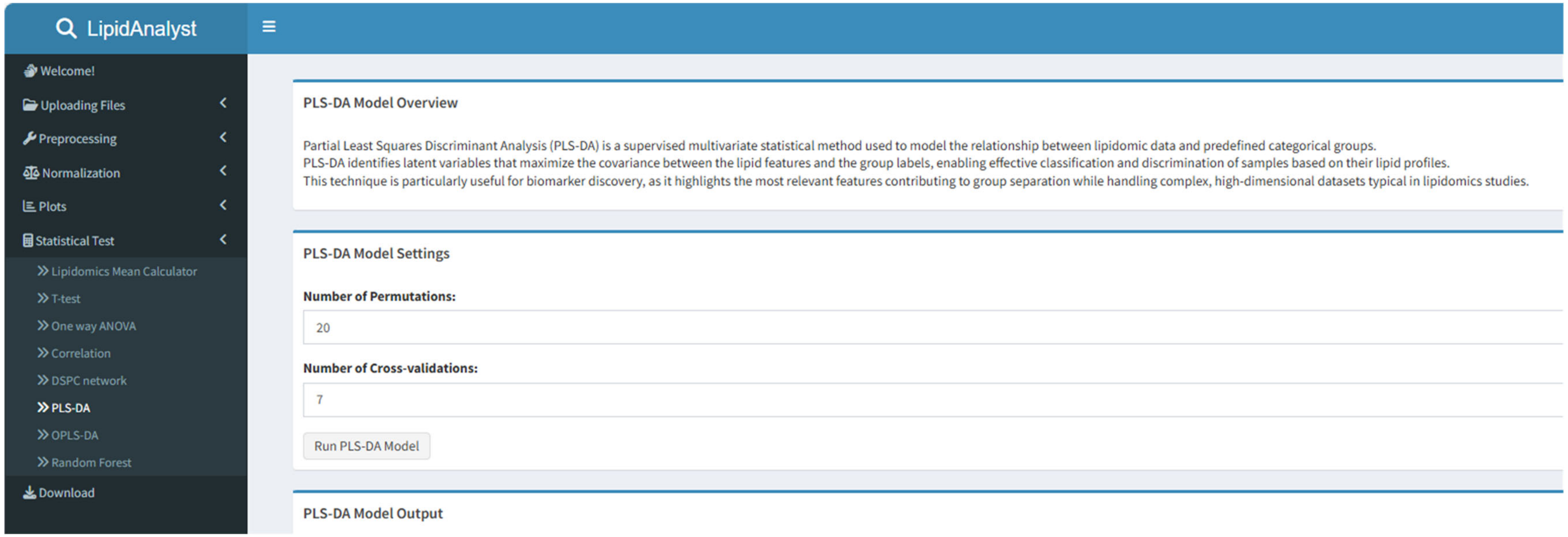

Figure S29A: PLS-DA (Partial Least Square Discriminant Analysis) is a supervised multivariate method that identifies features that best discriminate between predefined groups. Permutation testing evaluates the statistical significance of the model by randomly shuffling the sample group label and rebuilding the model to determine whether the observed performance of classification is better than random chance. The number of cross validation folds (k) determines how the dataset is split into k subsets, where one subset is used as the testing set and the remaining k−1 subsets are used as the training set, which help prevent overfitting. After defining the number of permutations and number of cross validations, users may visualize the model result by clicking on “Run PLS-DA Model”.

PLS-DA Model Output

Metric	Value
R2X(cum)	0.264
R2Y(cum)	0.593
Q2(cum)	0.048
RMSEE	0.338
pre	1
ort	0
pR2Y	0.05
pQ2	0.2

Figure S29B: PLS-DA Model Performance and Validation Metrics. R2X represents the cumulative proportion of variance in the predictor matrix (X) explained by the model. R2Y represents the cumulative proportion of variance in response variable (Y) and reflects the goodness of fit to class labels. Q2 evaluates model prediction ability based on cross validation. RMSEE (Root Mean Square Error of Estimation) indicates the model error in the training set. Pre and Ort denote the numbers of predictive and orthogonal components, respectively. pR^2Y and pQ^2 represent permutation test p-values for R^2Y and Q^2.

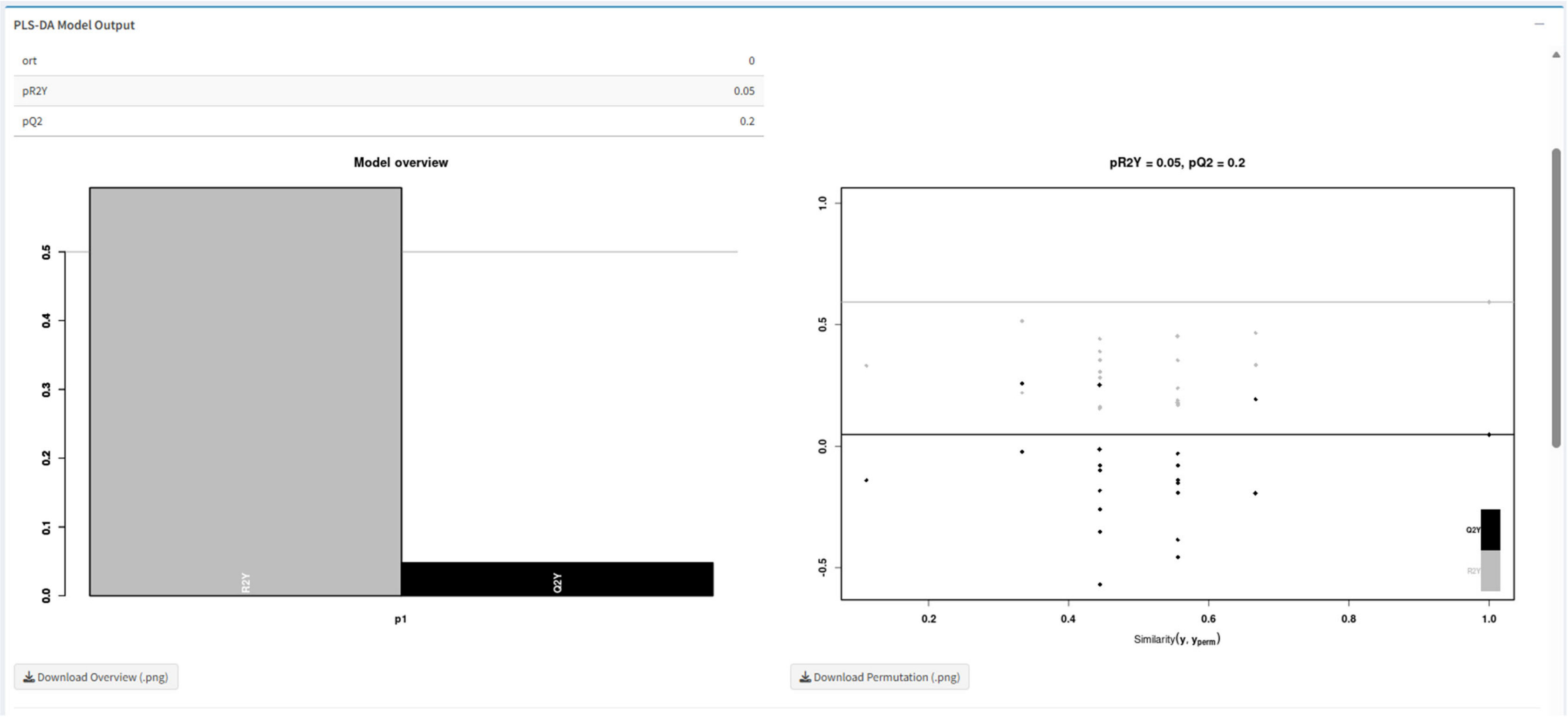

Figure S29C: PLS-DA overview plot and permutation plot. The overview plot provides a summary of the PLS-DA model. The permutation plot evaluates the statistical validity of the model by comparing the R2 and Q2 values from the permutation testing.

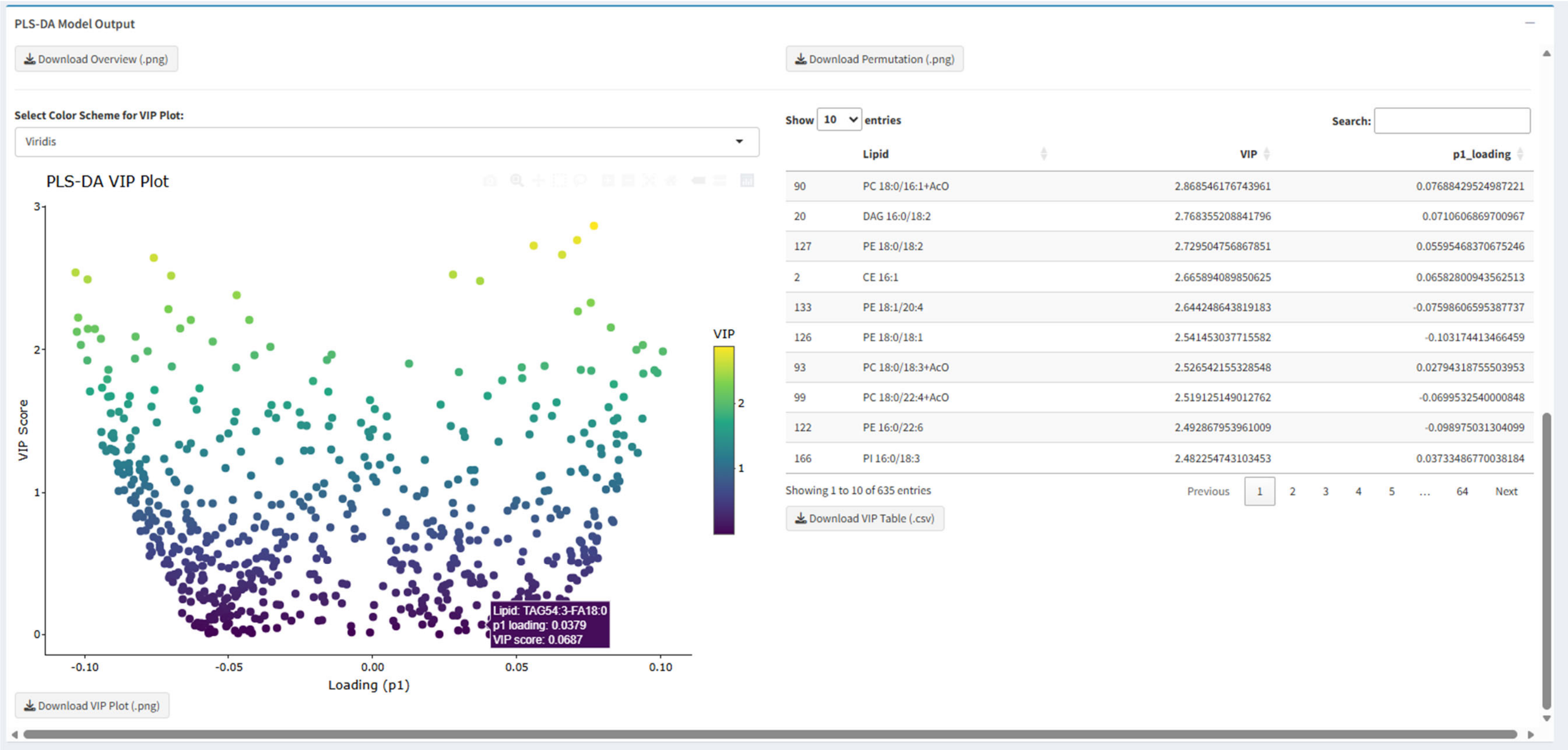

Figure S29D: The PLS-DA Variable Importance in Projection (VIP) plot highlights features that contribute substantially to class separation. The corresponding VIP table lists each feature's VIP score and its p1 loading value, representing the feature's contribution and projection onto the first predictive latent variable.

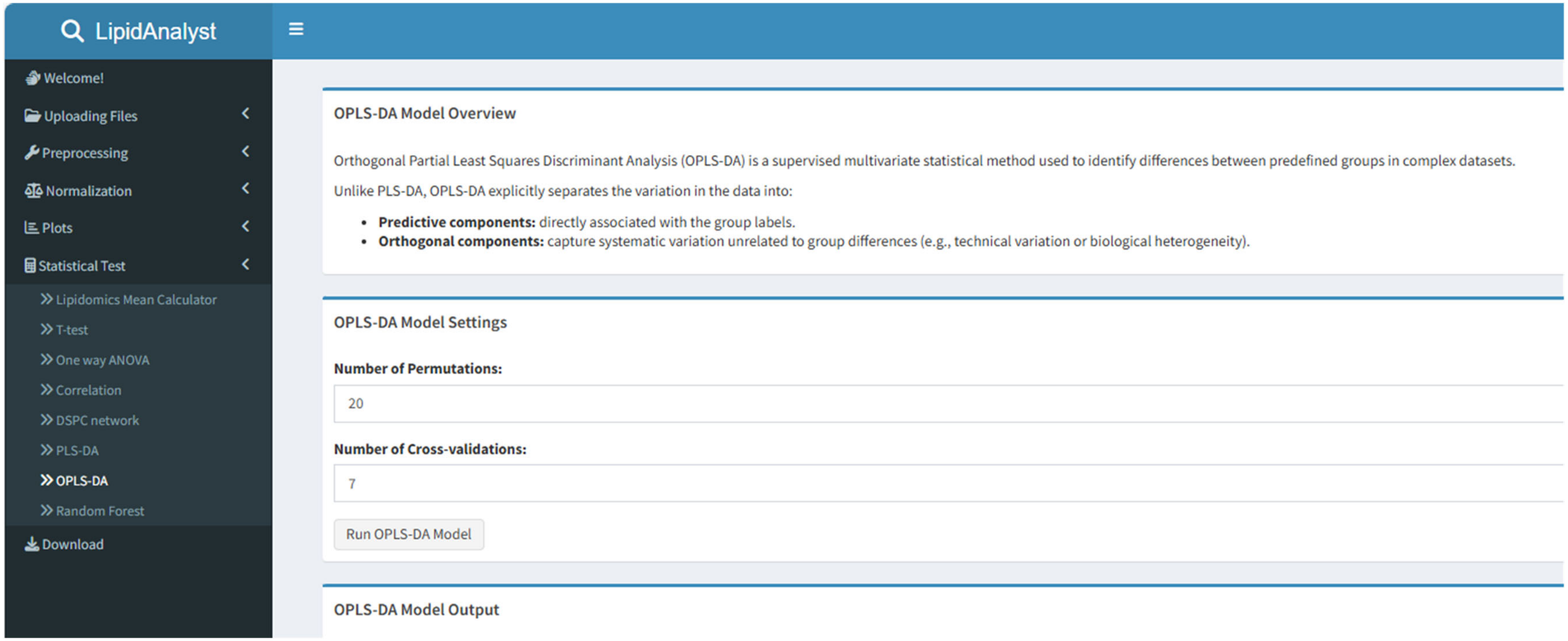

Figure S30A: OPLS-DA (Orthogonal Partial Least Square Discriminant Analysis) is a supervised multivariate method that identifies features that best discriminate between predefined groups with predictive components and orthogonal components. After defining the number of permutations and number of cross validation folds, users may visualize the model result by clicking on “Run OPLS-DA Model”.

OPLS-DA Model Output

Metric	Value
R2X(cum)	0.625
R2Y(cum)	0.956
Q2(cum)	0.641
RMSEE	0.119
pre	1
ort	2
pR2Y	0.05
pQ2	0.1

Figure S30B: OPLS-DA Model Performance and Validation Metrics. Model parameters are interpreted as described for the PLS-DA model.

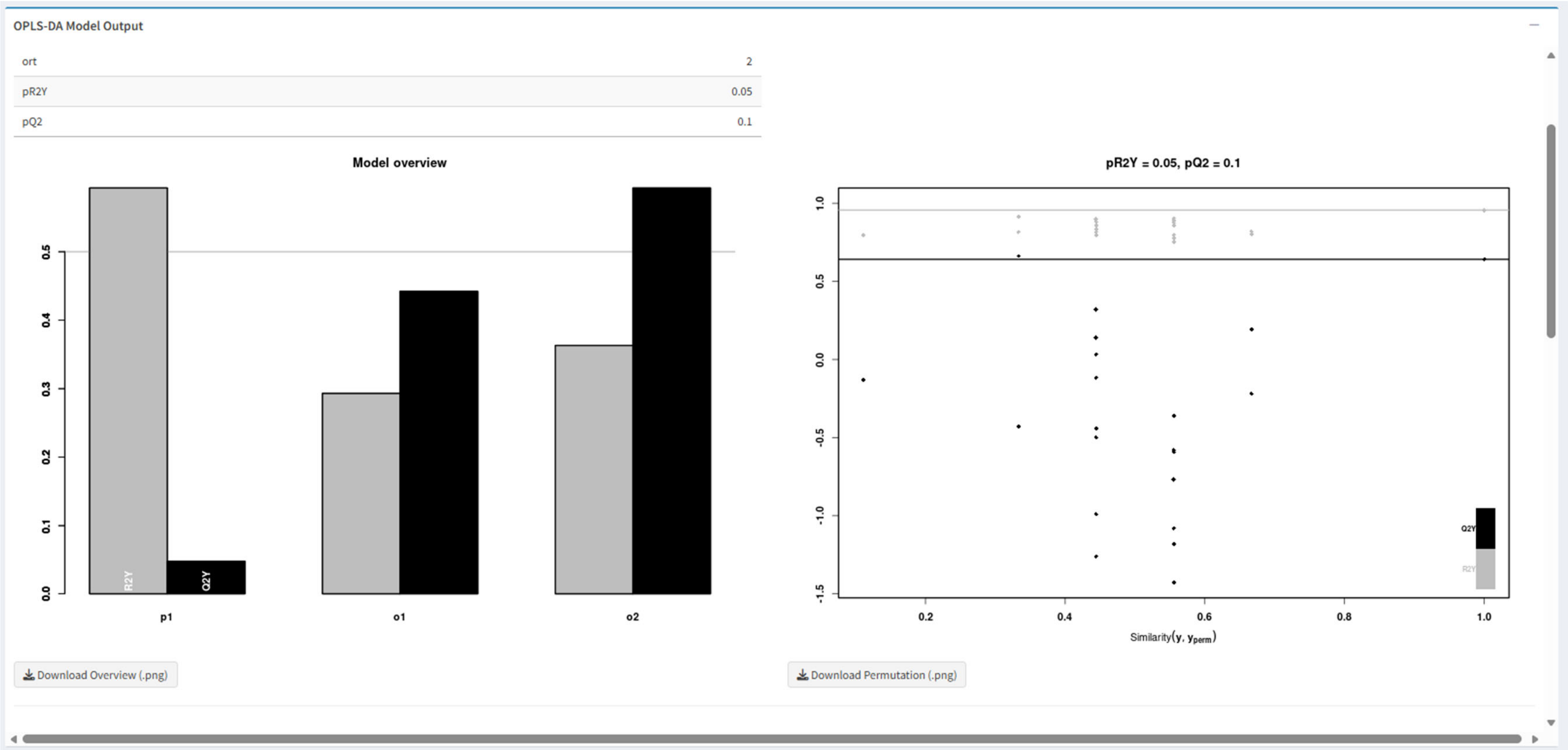

Figure S30C: OPLS-DA overview plot and permutation plot.

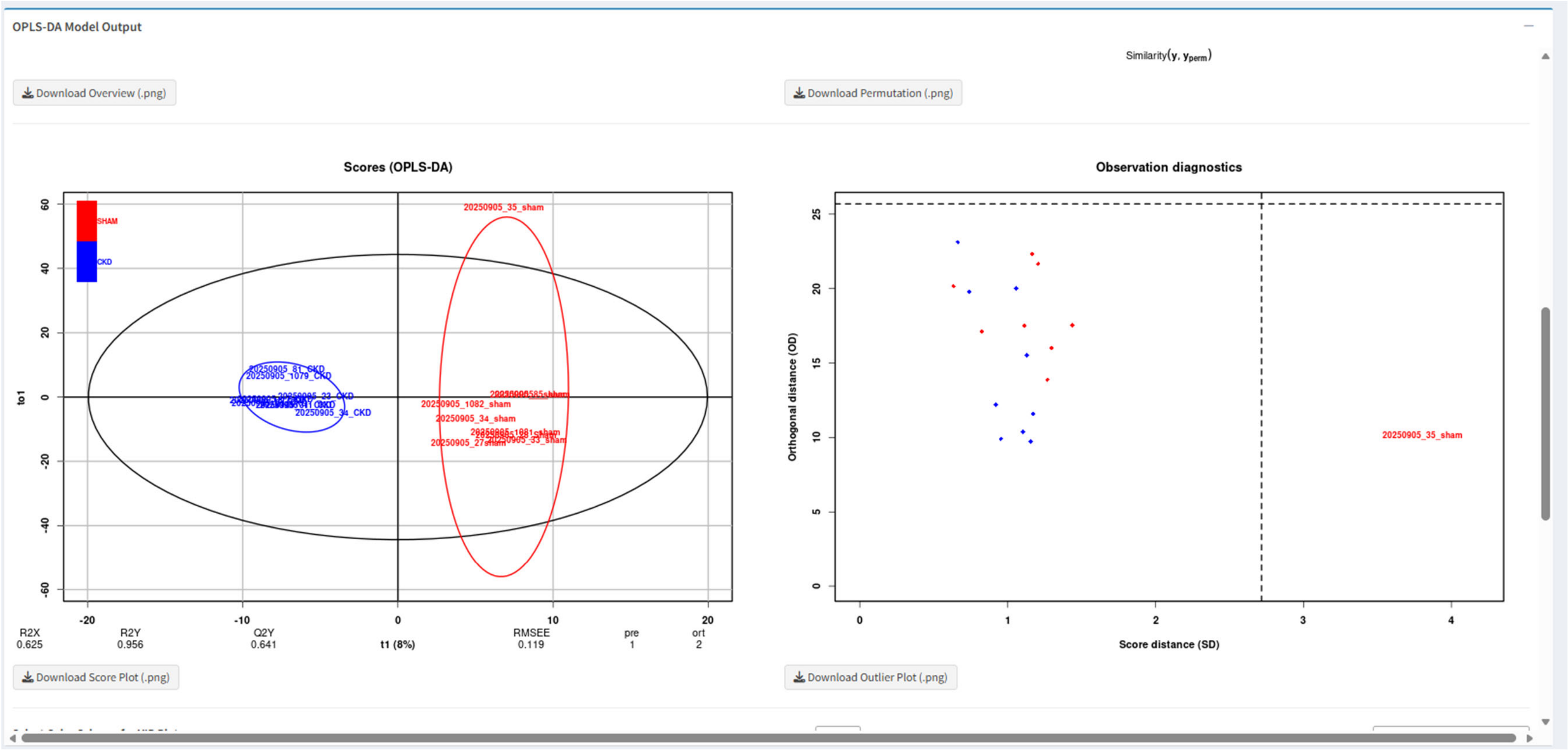

Figure S30D: OPLS-DA score plot and outlier plot. The OPLS-DA score plot shows the separation of samples between predefined groups. The outlier plot presents observation diagnostics for detecting potential outliers that may affect the model.

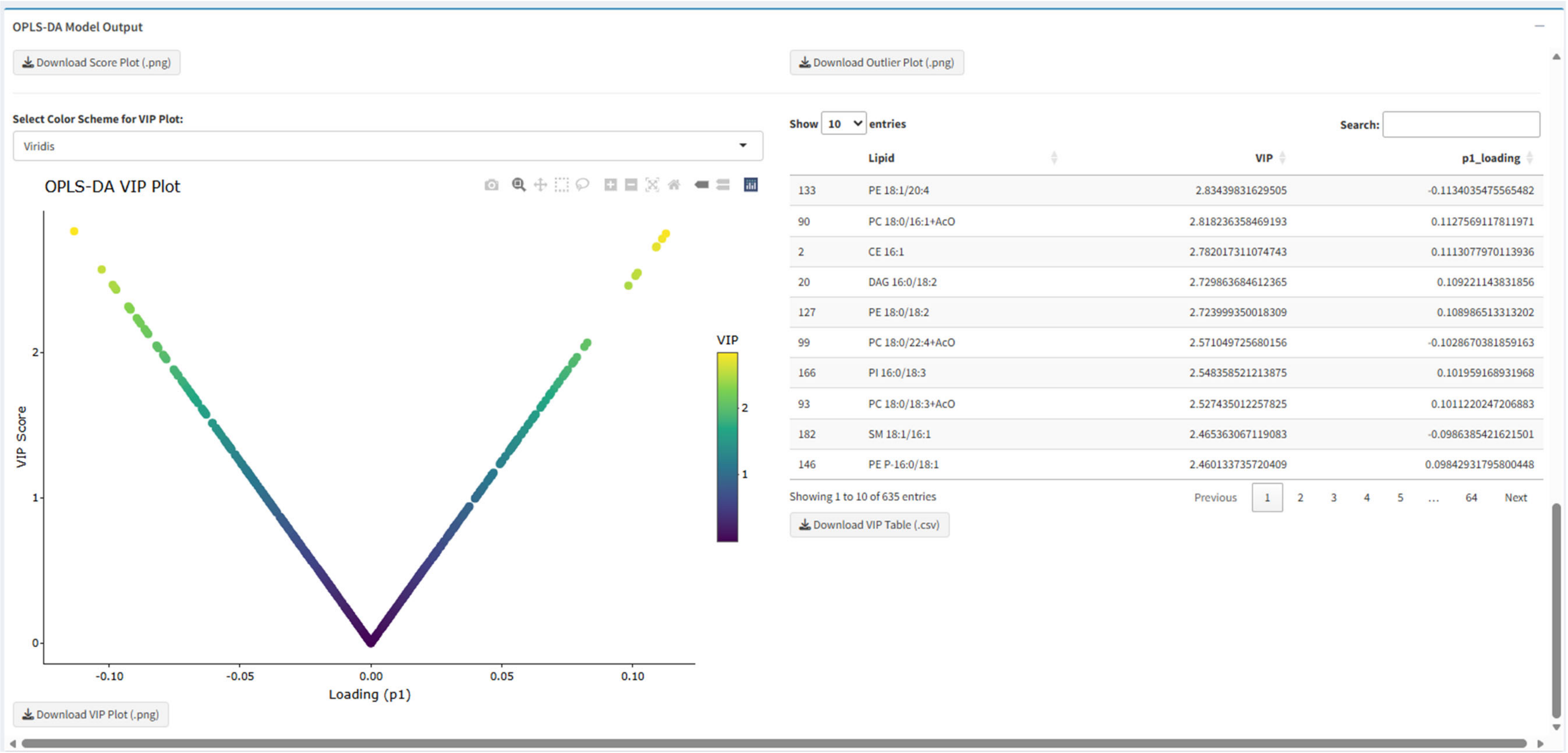

	Lipid	VIP	p1_loading
133	PE 18:1/20:4	2.83439831629505	-0.1134035475565482
90	PC 18:0/16:1+AcO	2.818236358469193	0.1127569117811971
2	CE 16:1	2.782017311074743	0.1113077970113936
20	DAG 16:0/18:2	2.729863684612365	0.109221143831856
127	PE 18:0/18:2	2.723999350018309	0.108986513313202
99	PC 18:0/22:4+AcO	2.571049725680156	-0.1028670381859163
166	PI 16:0/18:3	2.548358521213875	0.101959168931968
93	PC 18:0/18:3+AcO	2.527435012257825	0.1011220247206883
182	SM 18:1/16:1	2.465363067119083	-0.0986385421621501
146	PE P-16:0/18:1	2.460133735720409	0.09842931795800448

Figure S30E: OPLS VIP plots and VIP table.

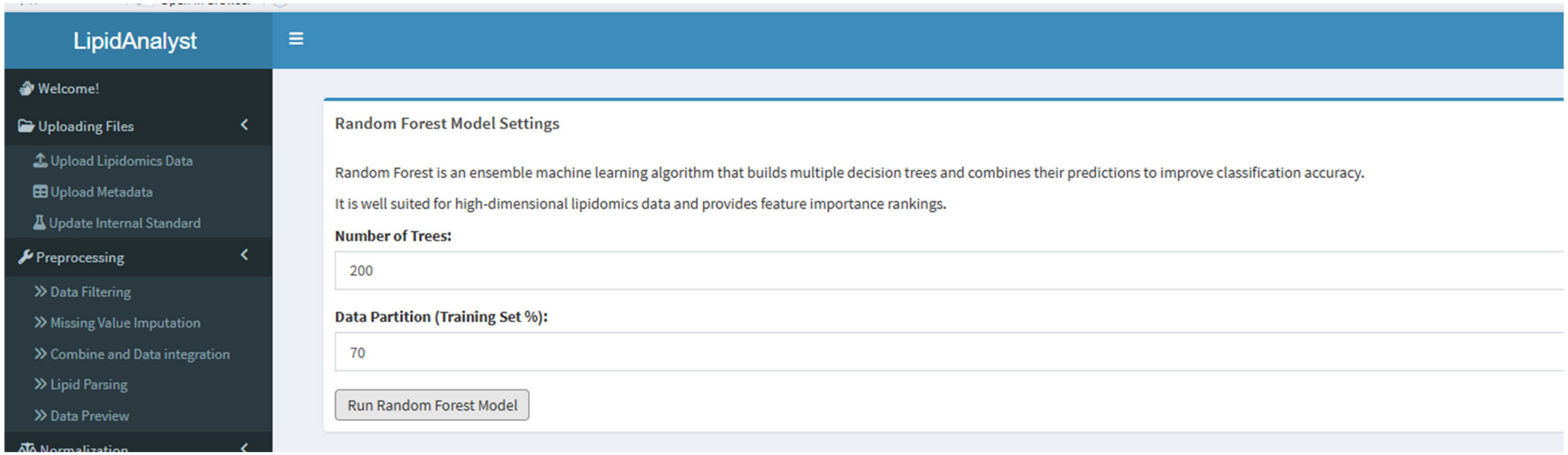

Figure S31A: Random Forest model is an ensemble machine learning technique that builds multiple decision trees using bootstrap sampling and random feature selection. Users can select how many trees the Random Forest will build and how the dataset is split between training and testing before running the model.

Random Forest Model Output

Metric	Value
Accuracy	1
Kappa	1
AccuracyLower	0.3976
AccuracyUpper	1
AccuracyNull	0.5
AccuracyPValue	0.0625
McnemarPValue	

Figure S31B: Random Forest Model Result Output. Accuracy represents the proportion of correctly classified samples. Cohen’s Kappa evaluates classification agreement beyond chance. AccuracyLower and AccuracyUpper denote the confidence interval of the accuracy estimate. AccuracyNull represents the accuracy expected by random chance. AccuracyPValue tests whether the observed accuracy is significantly higher than the null accuracy. McNemarPValue evaluates whether classification errors between classes are significantly different.

Show 10 entries Search:

	Lipid	MeanDecreaseGini	MeanDecreaseAccuracy
1	TG 54:0	0.4435551948051948	3.558846382795493
2	PE 18:0/18:0	0.2045833333333333	0.9756505287721401
3	LPE 18:0	0.202031746031746	2.413642704956343
4	DG 18:0/18:2	0.1788253968253968	1.002509414234171
5	TG 51:3	0.1733333333333334	1.857295032915348
6	PE 16:0/22:6	0.158313492063492	1.570910312552027
7	TG 52:1	0.1497301587301587	1.53038302628449
8	DG 16:0/18:2	0.1496825396825397	1.714946004468303
9	PEP- 18:0/20:4	0.1481071428571429	1.947361285876332
10	PC 14:0/18:1	0.1333531746031746	1.002509414234171

Showing 1 to 10 of 290 entries Previous 1 2 3 4 5 … 29 Next

Download Importance Table (.csv)

Figure S31C: Random Forest feature Importance table. Features importance in Random Forest model is assessed by Mean Decrease Gini and Mean Decrease Accuracy. Mean Decrease Gini measures the total decrease of Gini impurity of one feature across all trees, reflecting its role in class separation. Mean Decrease Accuracy measures the reduction of accuracy after one variable is permuted, reflecting the impact on predictive accuracy. Higher values indicate greater importance.

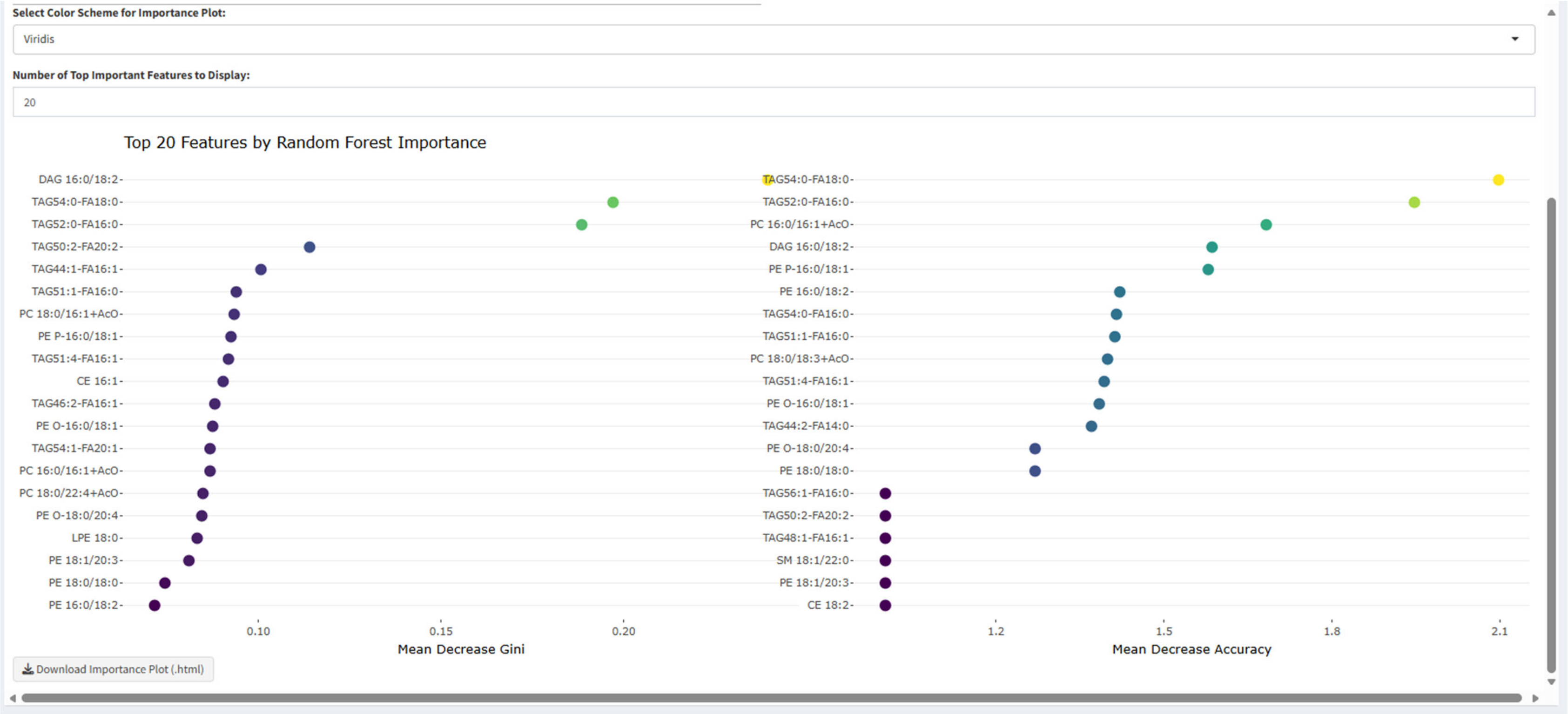

Figure S31D: Dot plots for top 20 features by Random Forest Importance.

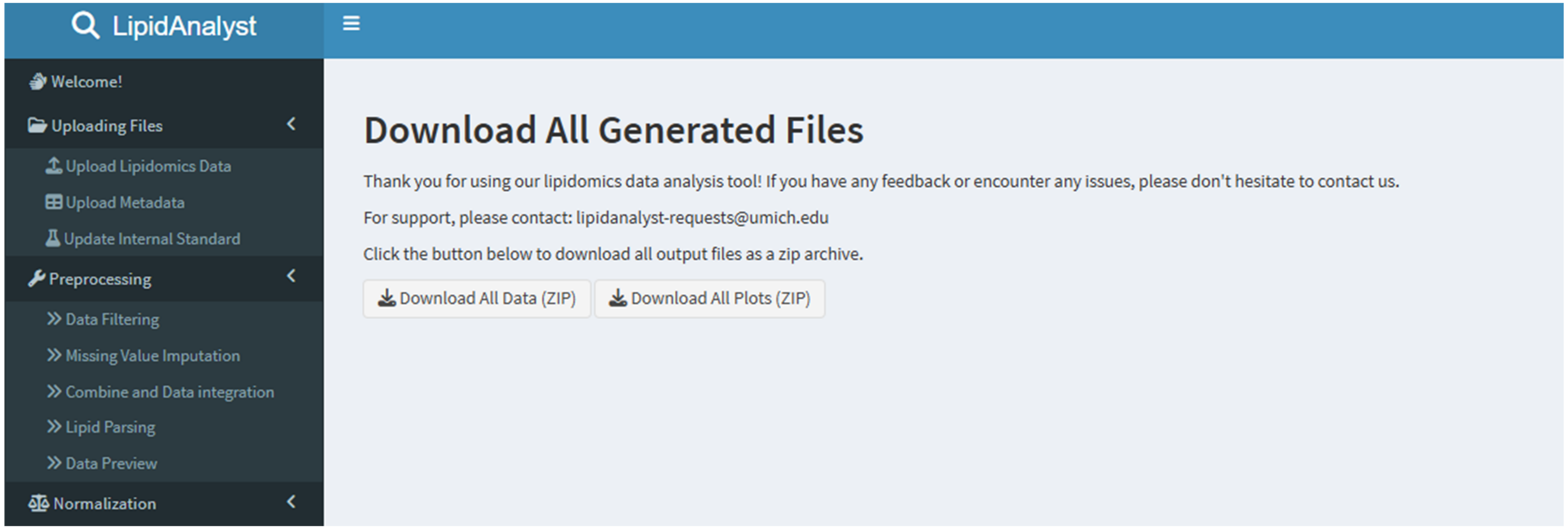

Figure S32: Downloading. At the end users have the option to download all the data files (ZIP) as well as any plots that they might have generated by clicking on appropriate tabs as above.

Figure S33: Recommended parameters settings

Module	Parameter	Default setting	Recommended use	When to modify
Feature Filtering	Missing value filter	Remove features missing in >50% of all samples	Recommended for most lipidomics datasets to avoid unreliable imputation.	Increasing the threshold retains more features but increases reliance on imputation, whereas decreasing the threshold improves robustness but may remove informative lipids.
Feature Filtering	Variance filtering	Disabled (Skip)	Recommended for most lipidomics datasets to avoid discarding potentially informative features.	Enabling variance filtering may improve multivariate model stability by reducing noise but may also remove biologically relevant low-variance lipids.
Feature Filtering	Abundance filtering	Disabled (Skip)	Recommended when all quantified lipids are considered biologically relevant.	Enabling abundance filtering reduces analytical noise but may exclude biologically relevant low-abundance lipids.
Missing value imputation	Missing value method	Sample-wise KNN	Suitable for most lipidomics datasets.	Group-wise minimum imputation for features absent in one group
Combine and Data Integration	Combine method	Sum	Sum abundances of duplicated lipid features representing the same lipid species.	Use Skip when duplicated entries represent distinct features rather than true duplicates. Use Mean when repeated measurements of the same lipid feature should be averaged (e.g., technical replicates).
PLS-DA;OPLS-DA	Permutation	500 permutations	Suitable for most exploratory analyses	Increase permutations for more rigorous significance assessment

PLS-DA / OPLS-DA	Cross-validation	7-fold cross-validation	Suitable for most exploratory lipidomics analyses and model optimization.	Adjust the number of folds according to sample size.
Random Forest	Number of trees	500 trees	Balances computational efficiency and stable feature ranking	Increase the number of trees (e.g., 1000) if greater stability of feature importance is desired
Random Forest	Data partition (Training/Test split)	Training set = 70%; Test set = 30%	Recommended for independent model evaluation when sufficient samples are available.	Increase the training proportion (e.g., 80–90%) for larger datasets

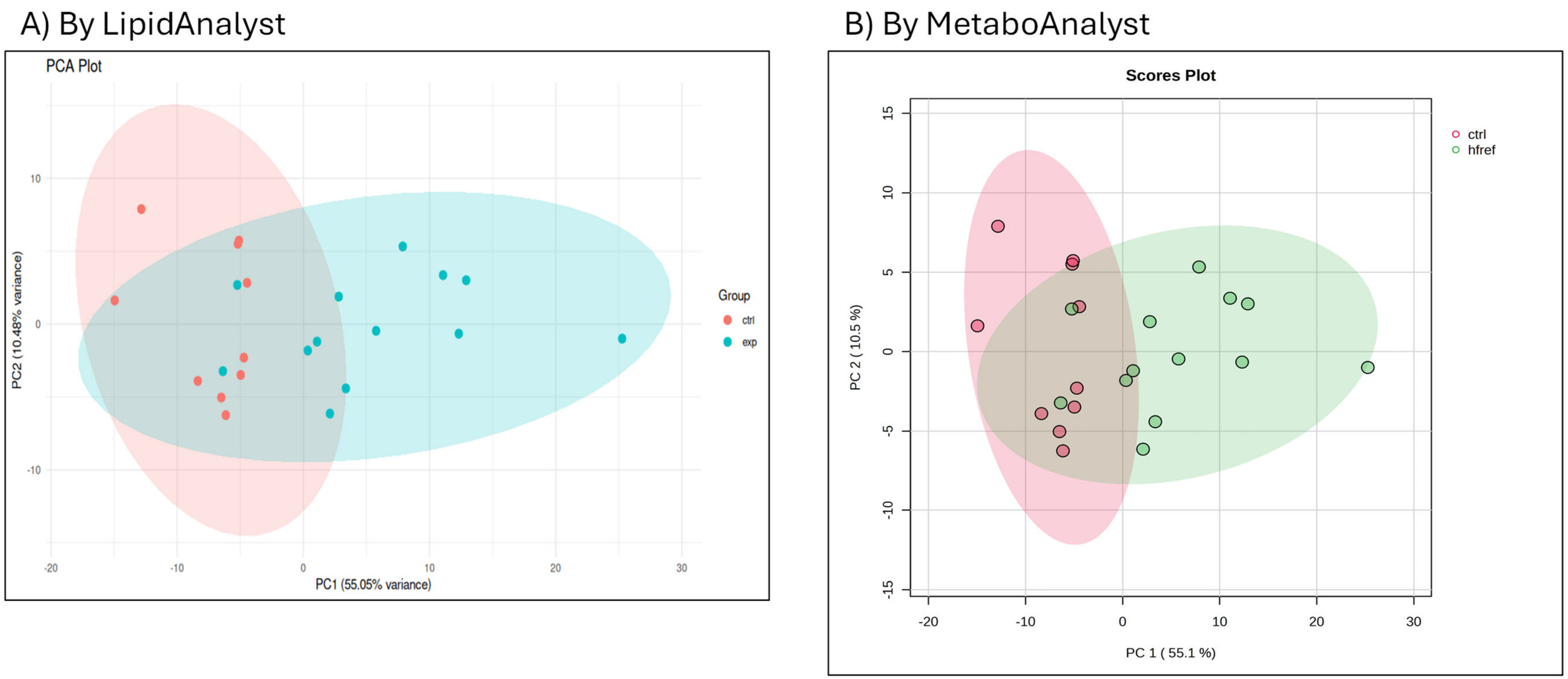

Figure S34: Comparison of PCA Results Generated by LipidAnalyst and MetaboAnalyst Using the Processed HFrEF Dataset

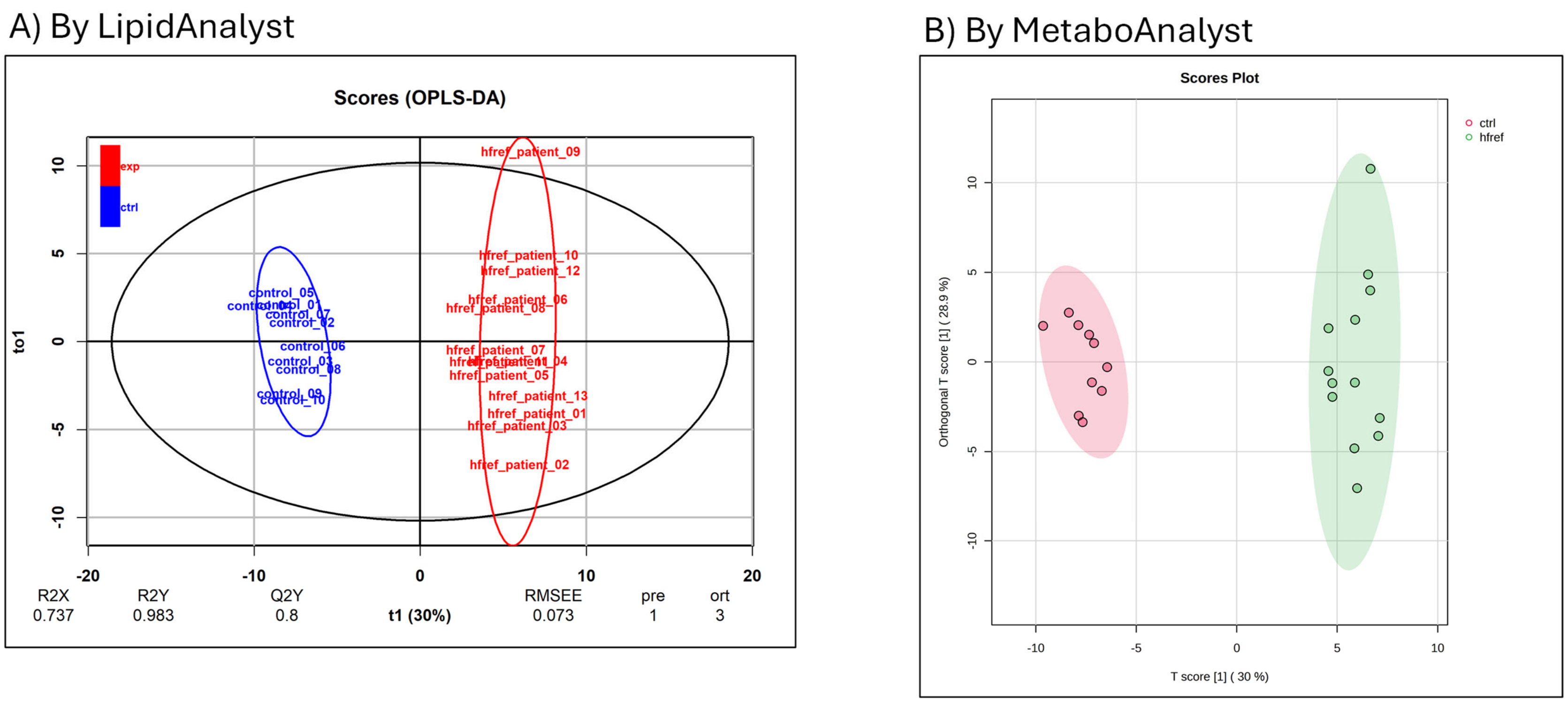

Figure S35: Comparison of OPLS-DA Score Plots Generated by LipidAnalyst and MetaboAnalyst Using the Processed HFrEF Dataset

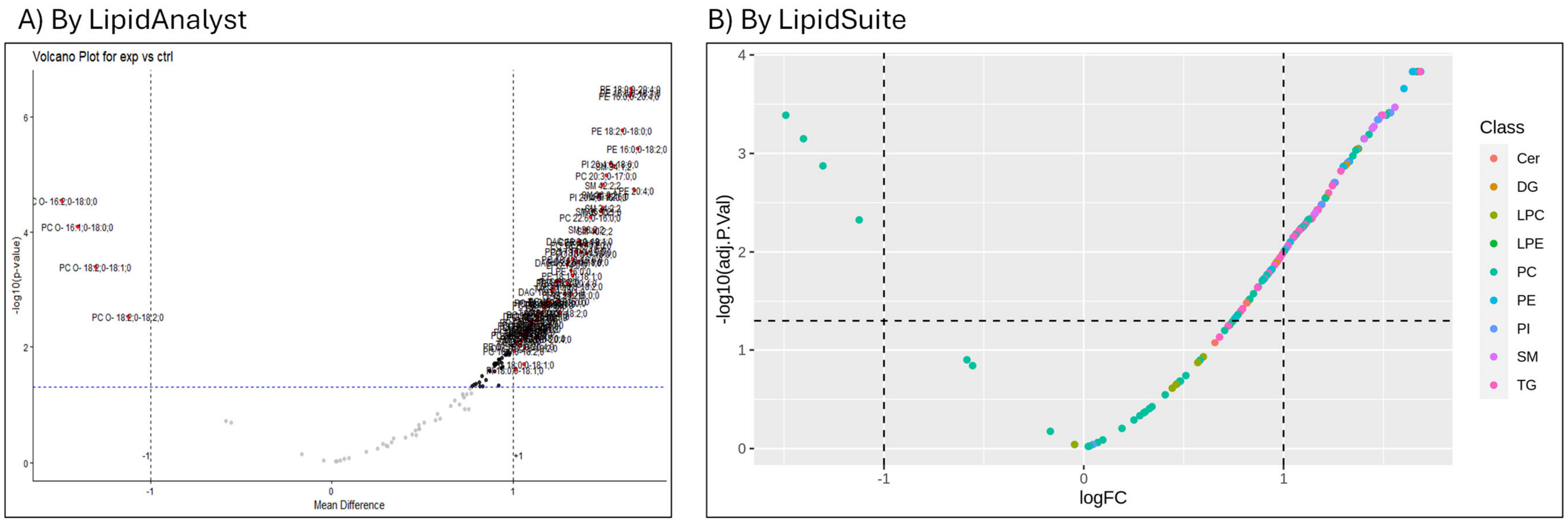

Figure S36: Comparison of Volcano Plots Generated by LipidAnalyst and LipidSuite Using the Processed HFrEF Dataset

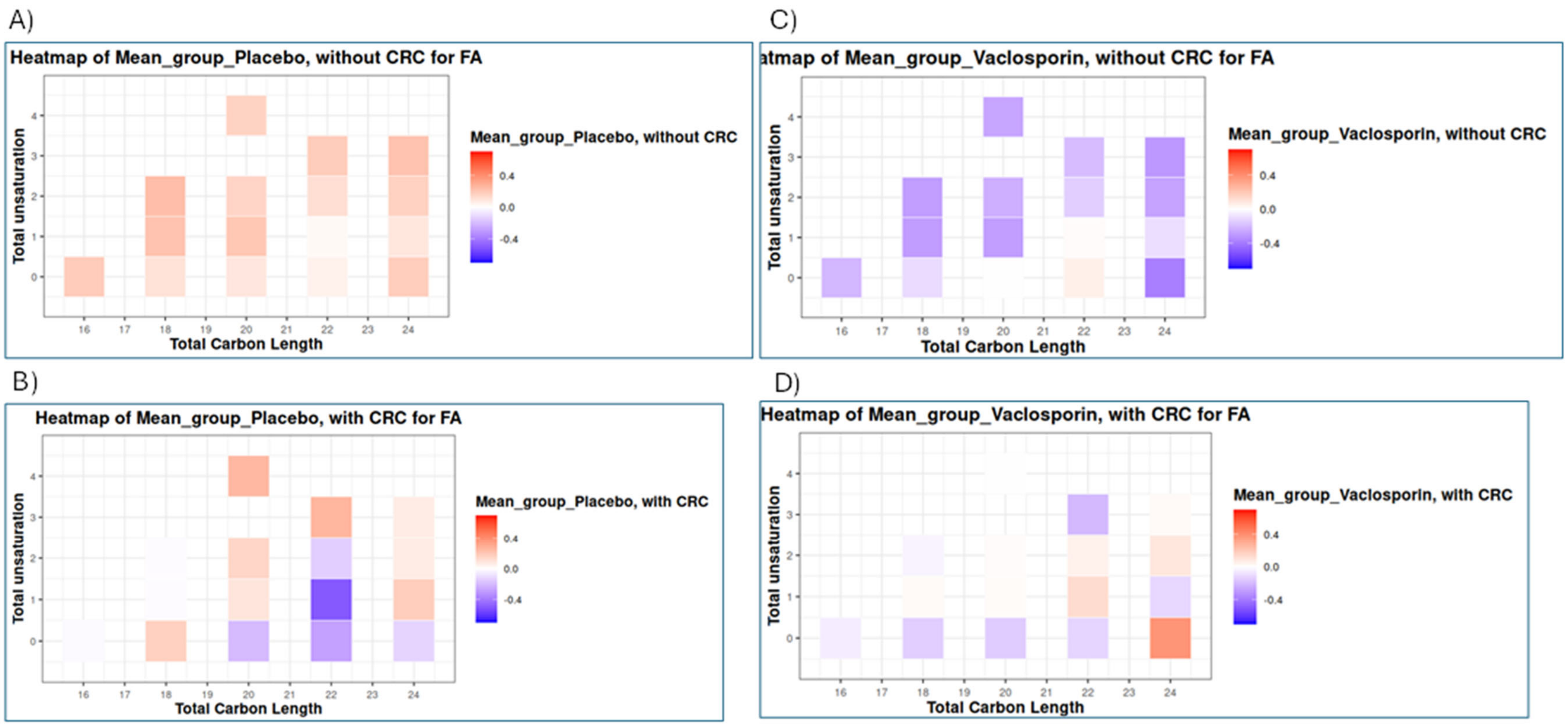

Figure S37: Replication of DMLH of free fatty acids (FFA) in participants of lipidomic study of voclosporin in systemic lupus erythematosus. Panel A represents no clinical response in placebo group. Panel B represents clinical response in placebo group. Panel C represents no clinical response in voclosporin group. Panel D represents clinical response in voclosporin group.

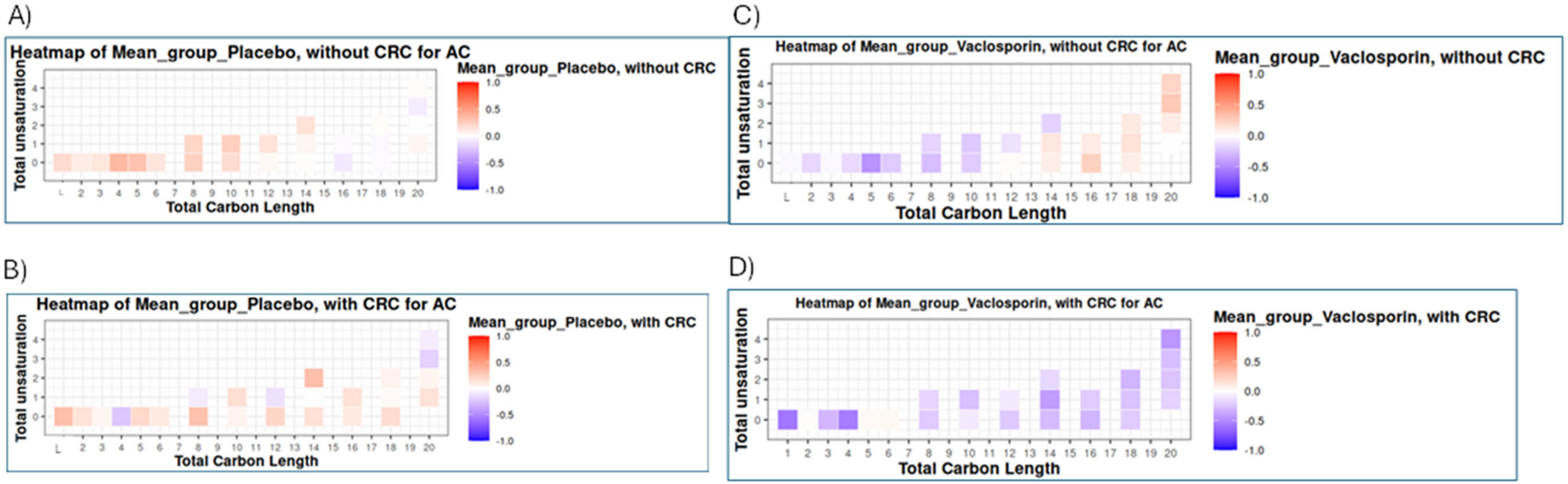

Figure S38: Replication of DMLH of acylcarnitines (AC) in participants of lipidomic study of voclosporin in systemic lupus erythematosus. Panel A represents no clinical response in placebo group. Panel B represents clinical response in placebo group. Panel C represents no clinical response in voclosporin group. Panel D represents clinical response in voclosporin group

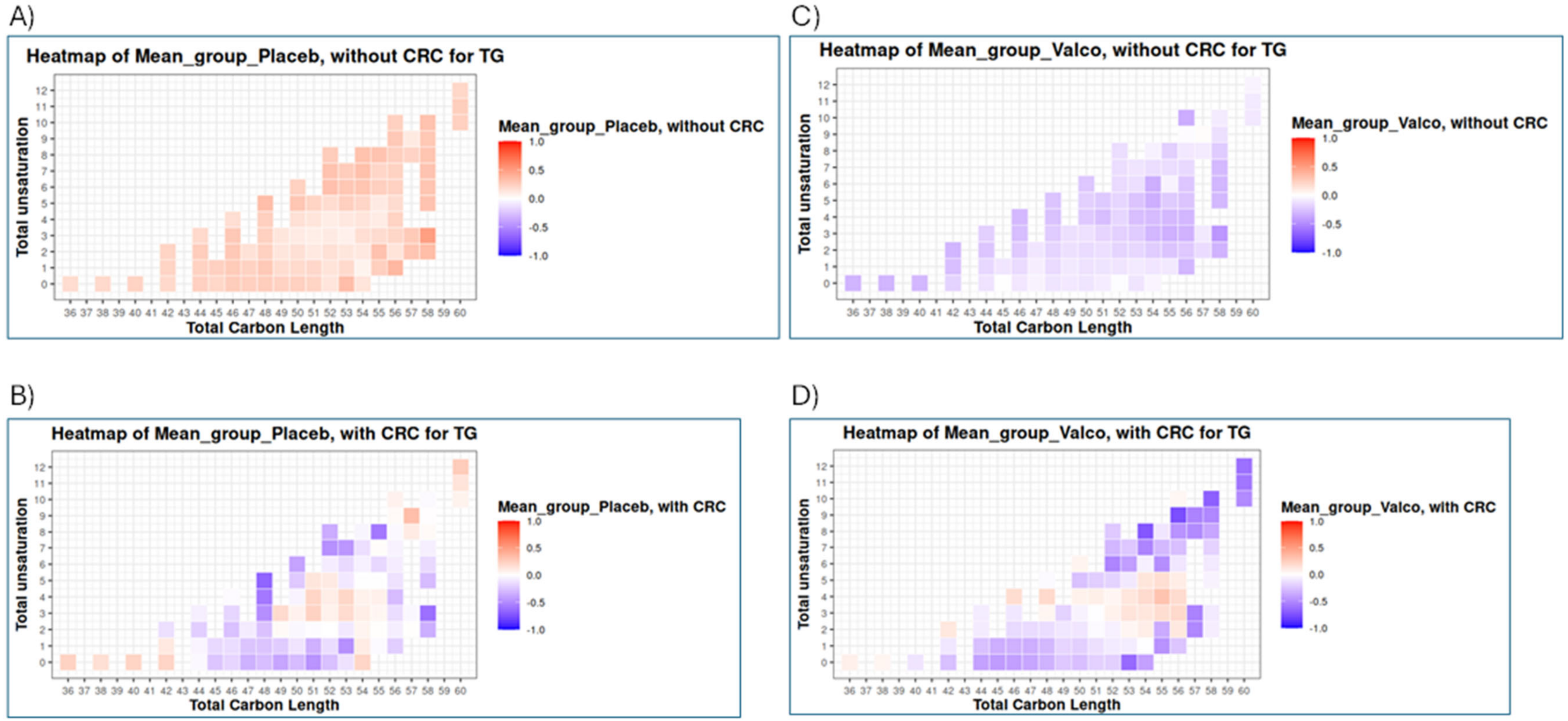

Figure S39: Replication of DMLH of triacylglycerols (TG) in participants of lipidomic study of voclosporin in systemic lupus erythematosus. Panel A represents no clinical response in placebo group. Panel B represents clinical response in placebo group. Panel C represents no clinical response in voclosporin group. Panel D represents clinical response in voclosporin group.

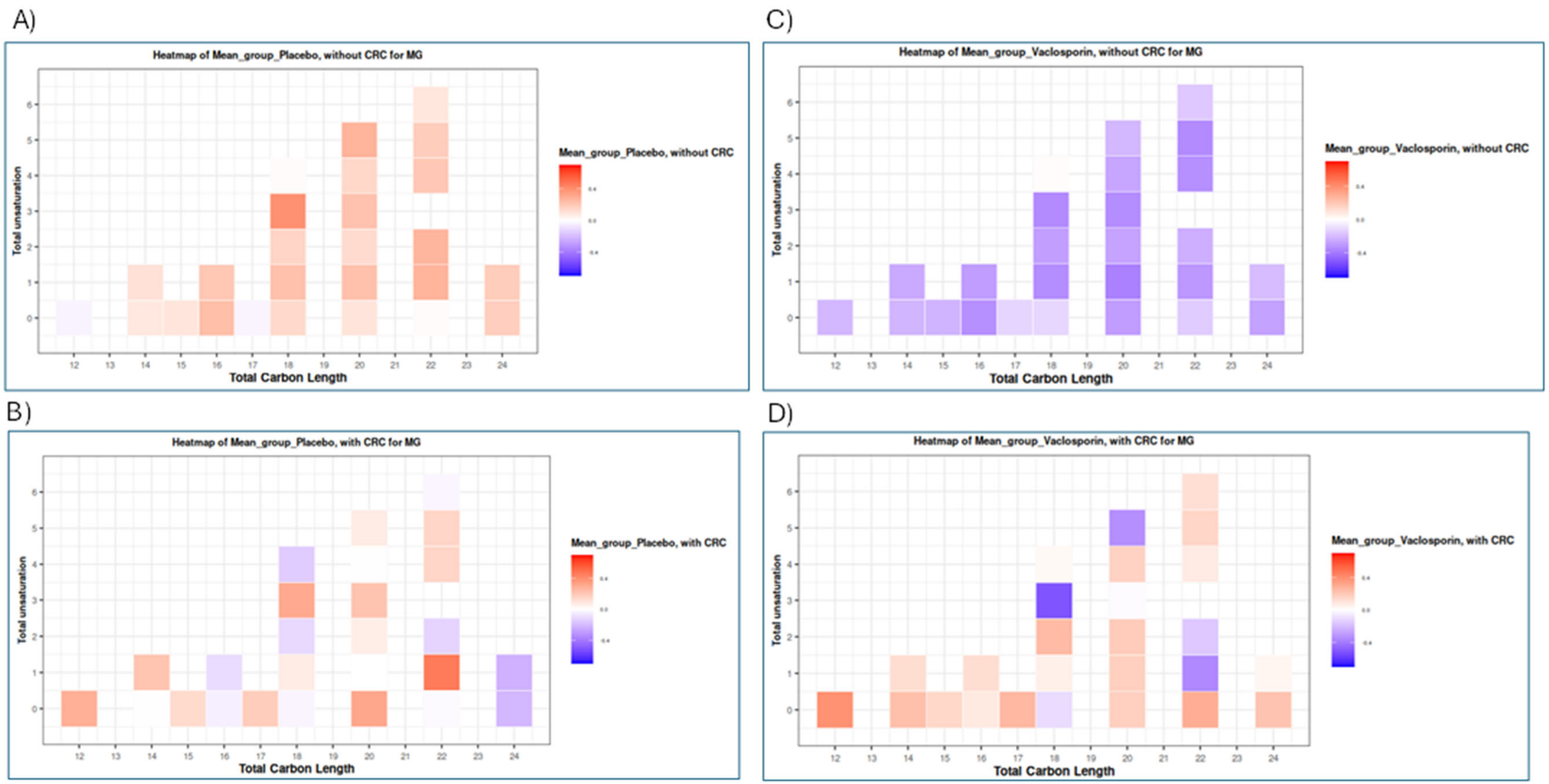

Figure S40: Replication of DMLH of monoacylglycerols (MAG) in participants of lipidomic study of voclosporin in systemic lupus erythematosus. Panel A represents no clinical response in placebo group. Panel B represents clinical response in placebo group. Panel C represents no clinical response in voclosporin group. Panel D represents clinical response in voclosporin group.

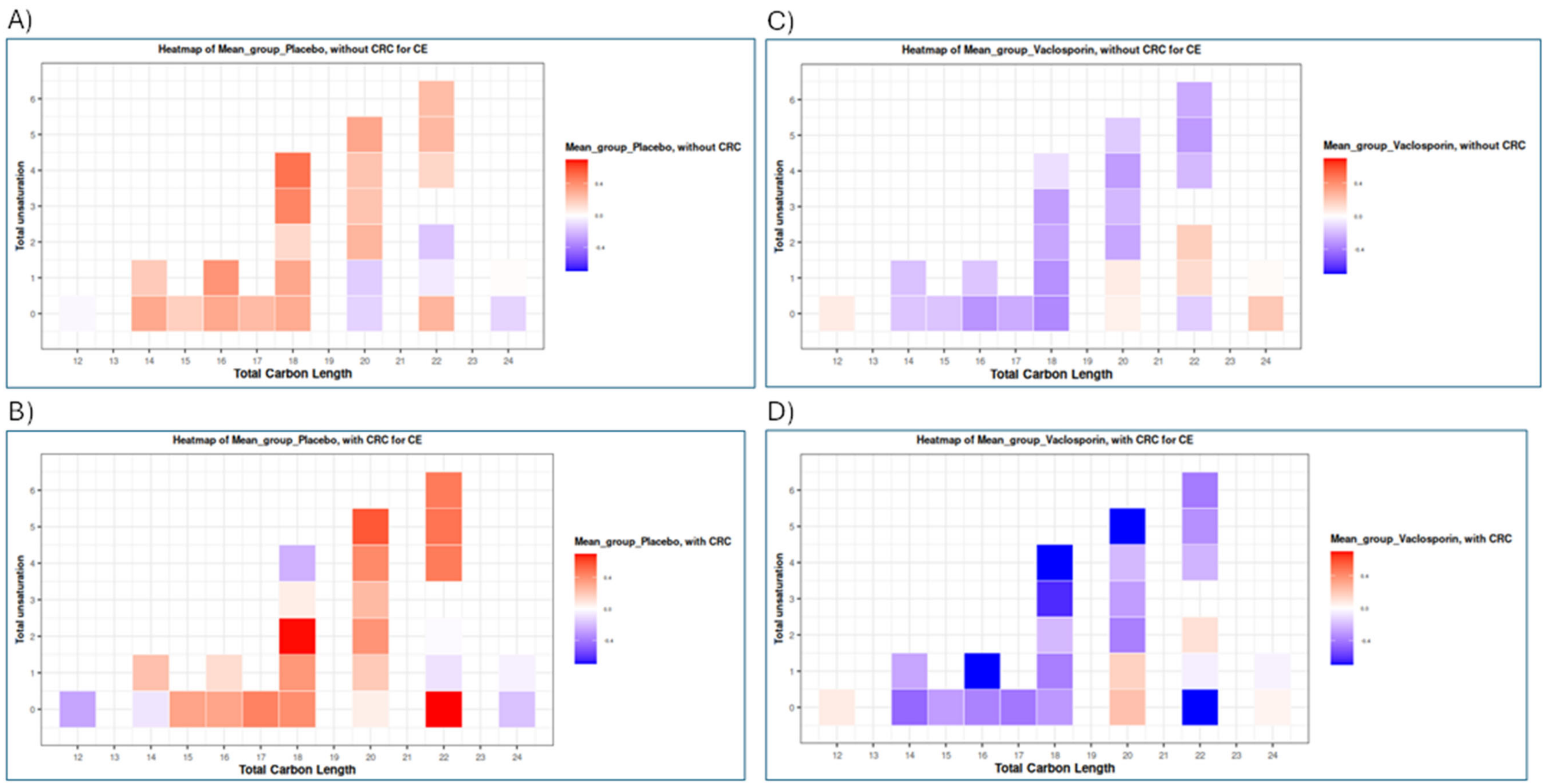

Figure S41: Replication of DMLH of cholesteryl esters (CE) in participants of lipidomic study of voclosporin in systemic lupus erythematosus. Panel A represents no clinical response in placebo group. Panel B represents clinical response in placebo group. Panel C represents no clinical response in voclosporin group. Panel D represents clinical response in voclosporin group.

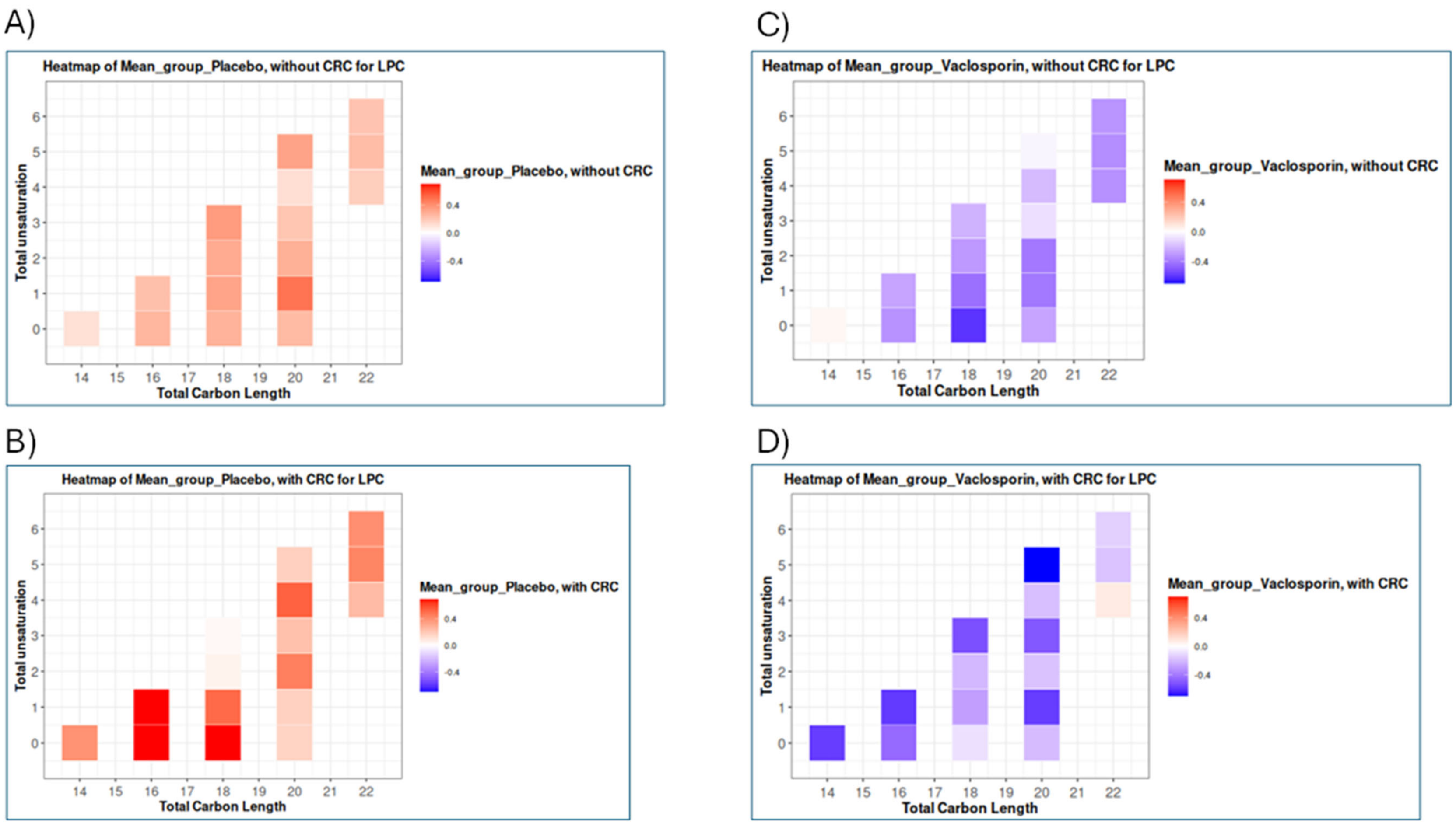

Figure S42: Replication of DMLH of lysophosphatidylcholines (LPC) in participants of lipidomic study of voclosporin in systemic lupus erythematosus. Panel A represents no clinical response in placebo group. Panel B represents clinical response in placebo group. Panel C represents no clinical response in voclosporin group. Panel D represents clinical response in voclosporin group.

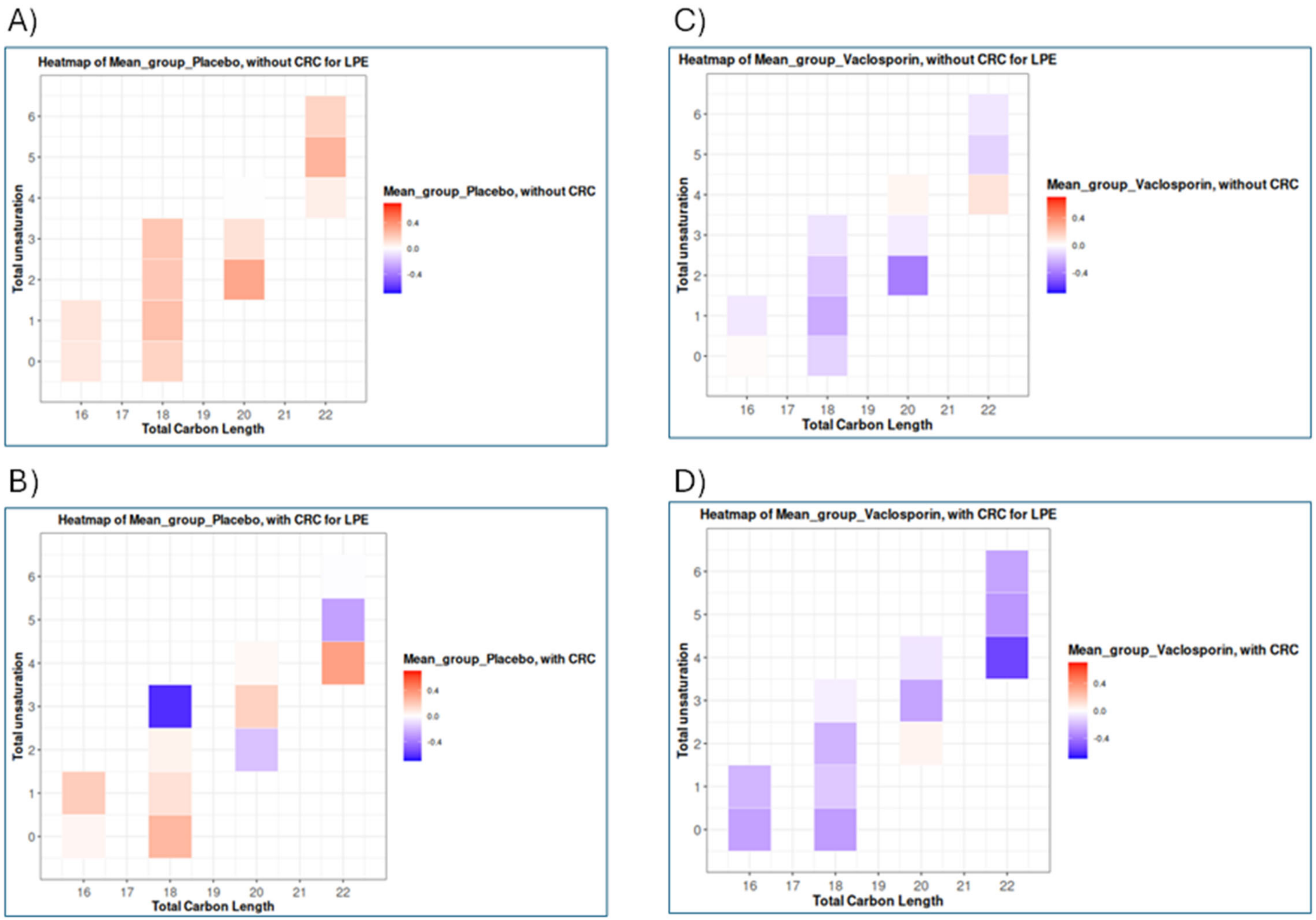

Figure S43: Replication of DMLH of lysophosphatidylethanolamines (LPE) in participants of lipidomic study of voclosporin in systemic lupus erythematosus. Panel A represents no clinical response in placebo group. Panel B represents clinical response in placebo group. Panel C represents no clinical response in voclosporin group. Panel D represents clinical response in voclosporin group.

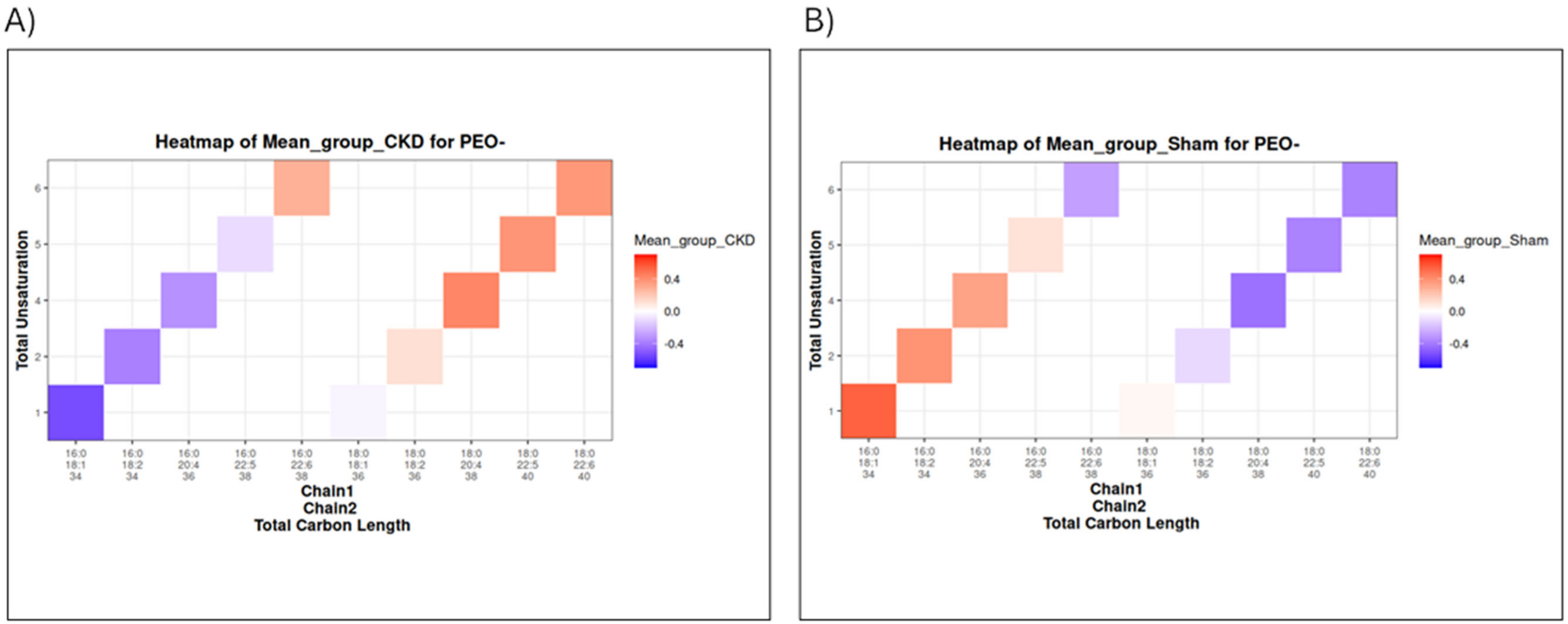

Figure S44: Sensitivity analysis of DMLH of phosphatidylethanolamine-O (PE-O) with lipid class sum normalization, logit transformation, and auto-scaling. Panel A represents the chronic kidney disease group. Panel B represents the sham group.

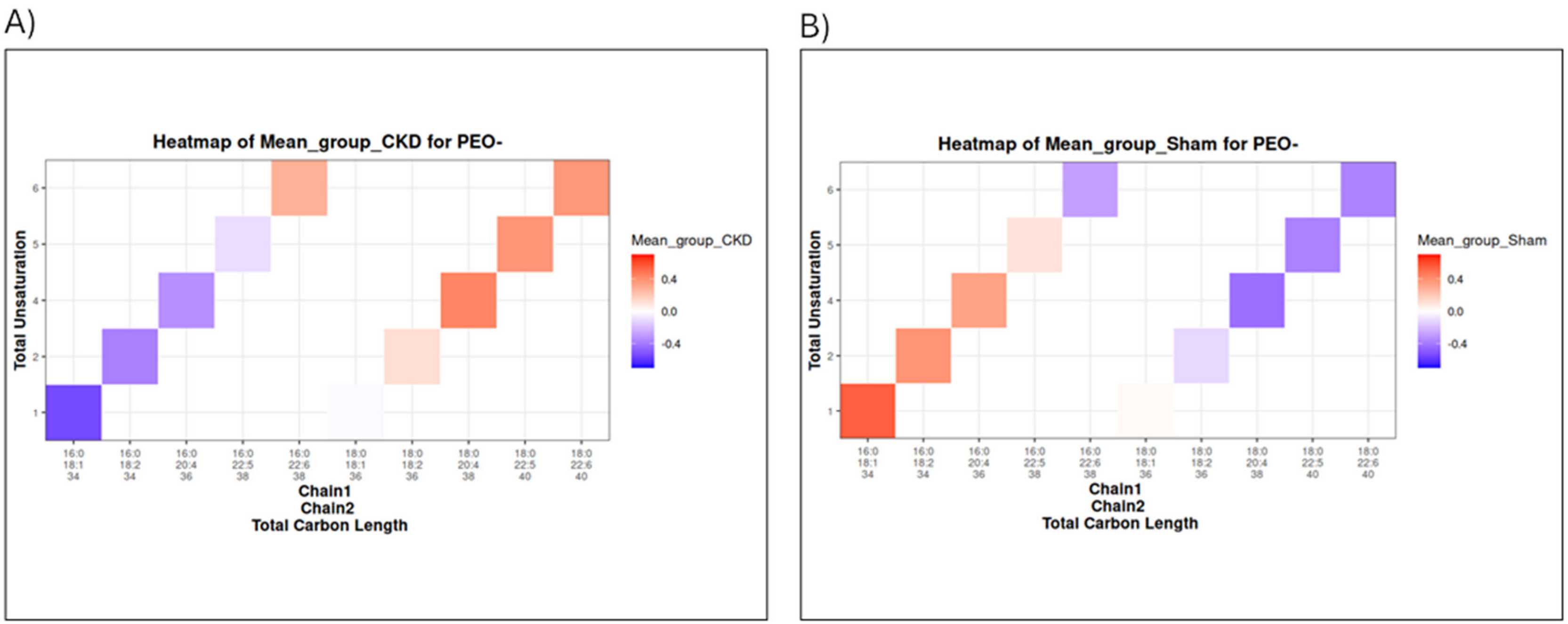

Figure S45: Sensitivity analysis of DMLH of phosphatidylethanolamine-O (PE-O) with lipid class mean normalization, log transformation, and auto-scaling. Panel A represents the chronic kidney disease group. Panel B represents the sham group.

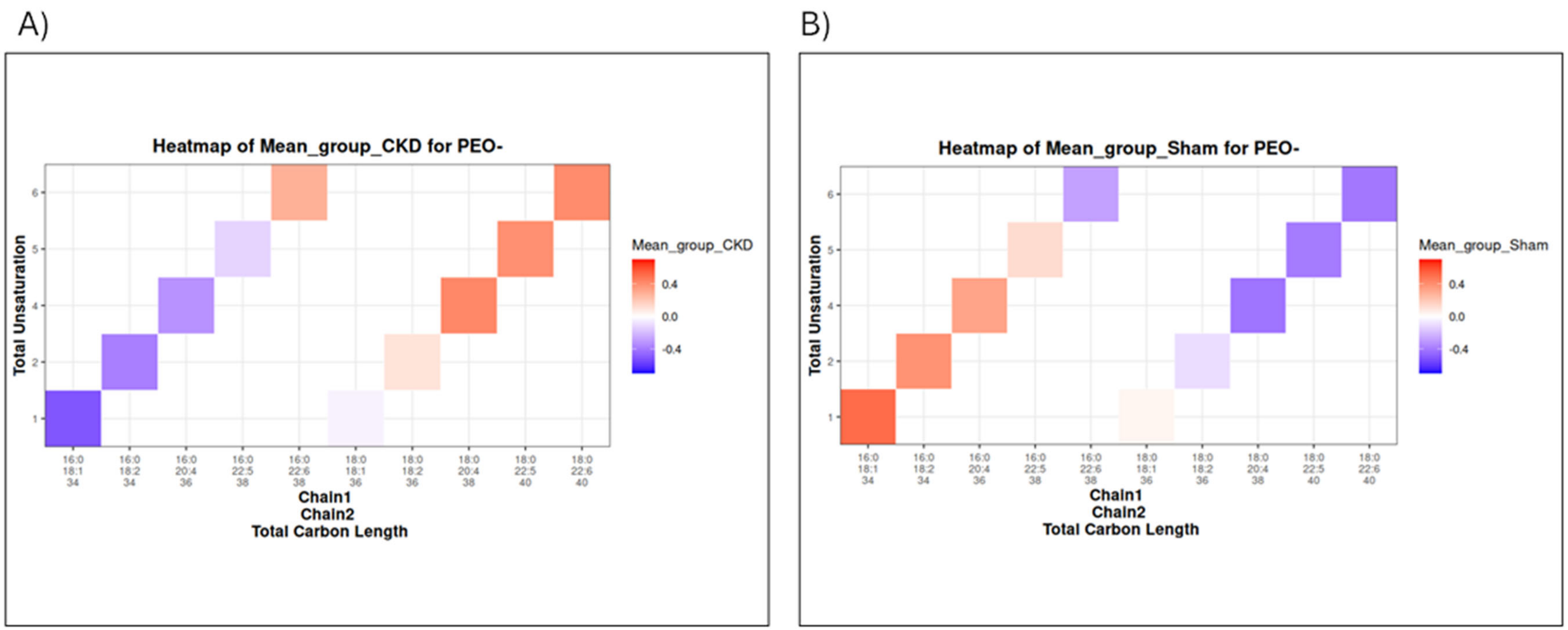

Figure S46: Sensitivity analysis of DMLH of phosphatidylethanolamine-O (PE-O) with lipid class mean normalization, square root transformation, and auto-scaling. Panel A represents the chronic kidney disease group. Panel B represents the sham group.

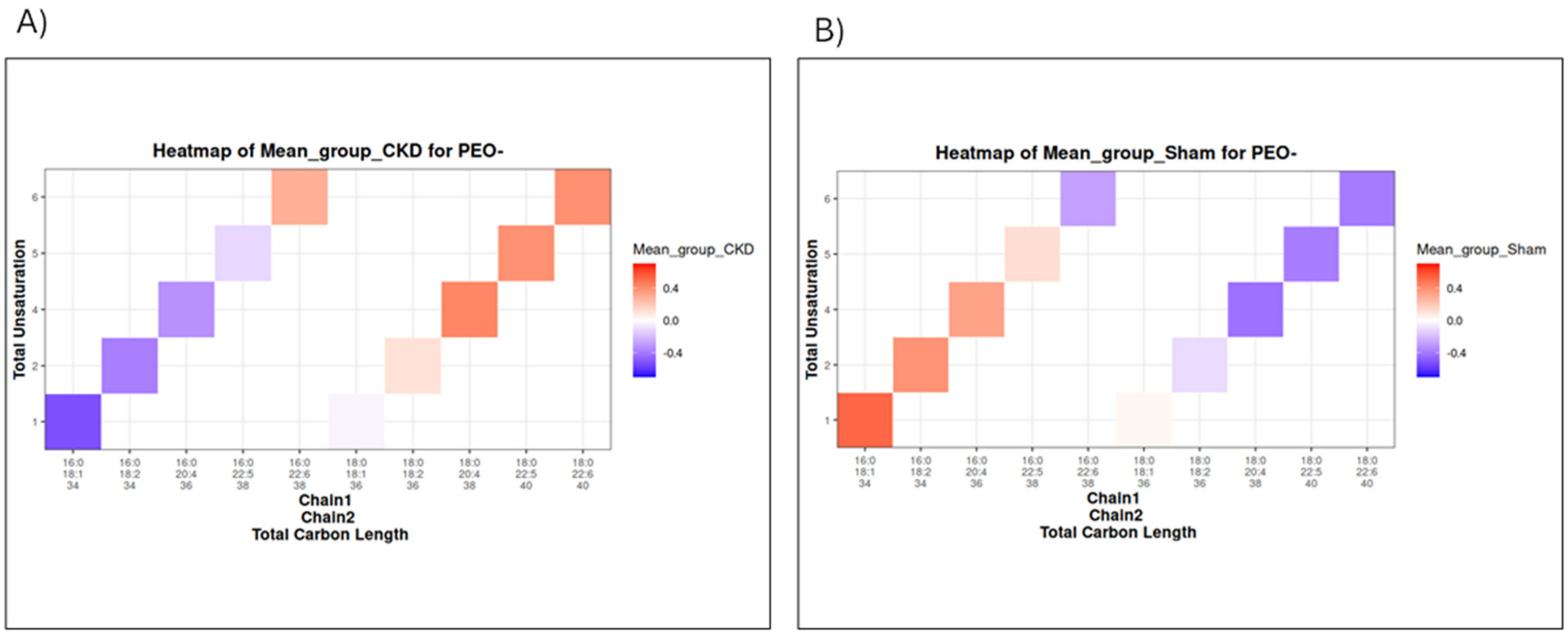

Figure S47: Sensitivity analysis of DMLH of phosphatidylethanolamine-O (PE-O) with lipid class mean normalization, cubic root transformation, and auto-scaling. Panel A represents the chronic kidney disease group. Panel B represents the sham group.

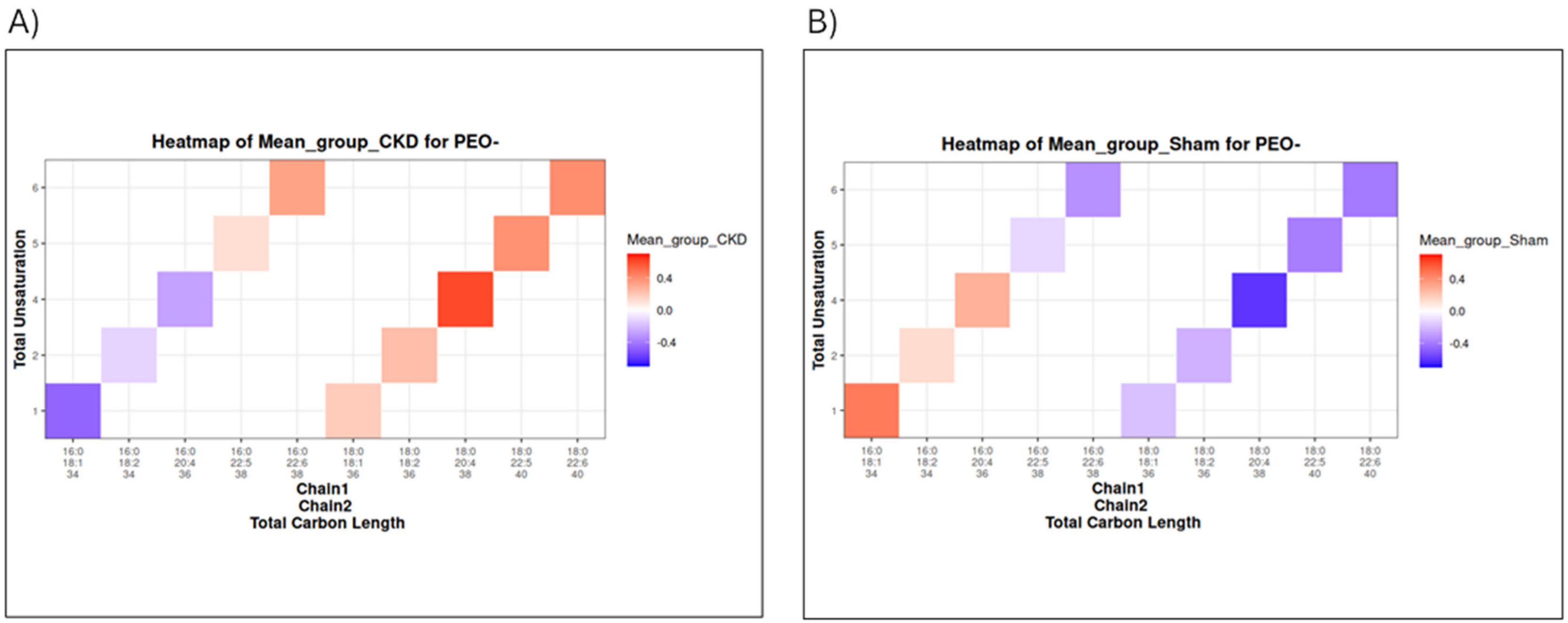

Figure S48: Sensitivity analysis of DMLH of phosphatidylethanolamine-O (PE-O) with lipid class median normalization, loge transformation, and auto-scaling. Panel A represents the chronic kidney disease group. Panel B represents the sham group.

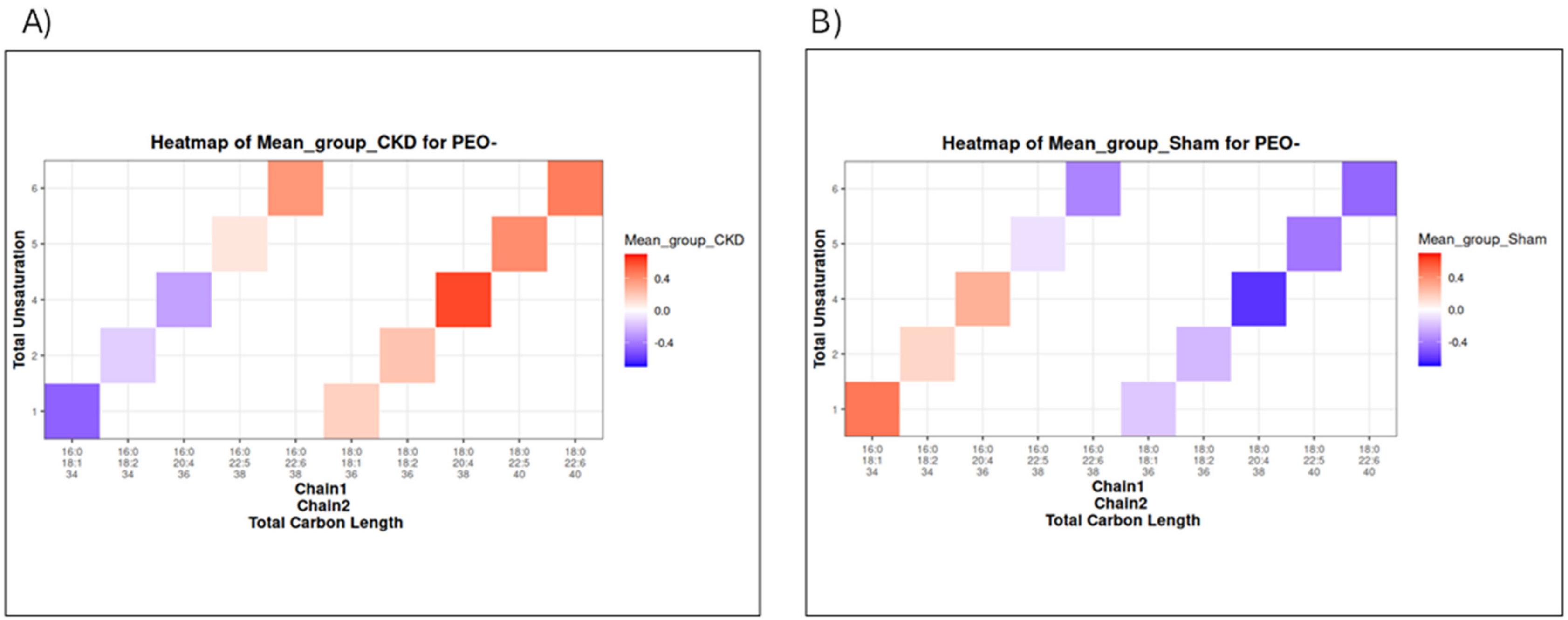

Figure S49: Sensitivity analysis of DMLH of phosphatidylethanolamine-O (PE-O) with lipid class median normalization, square root transformation, and auto-scaling. Panel A represents the chronic kidney disease group. Panel B represents the sham group.

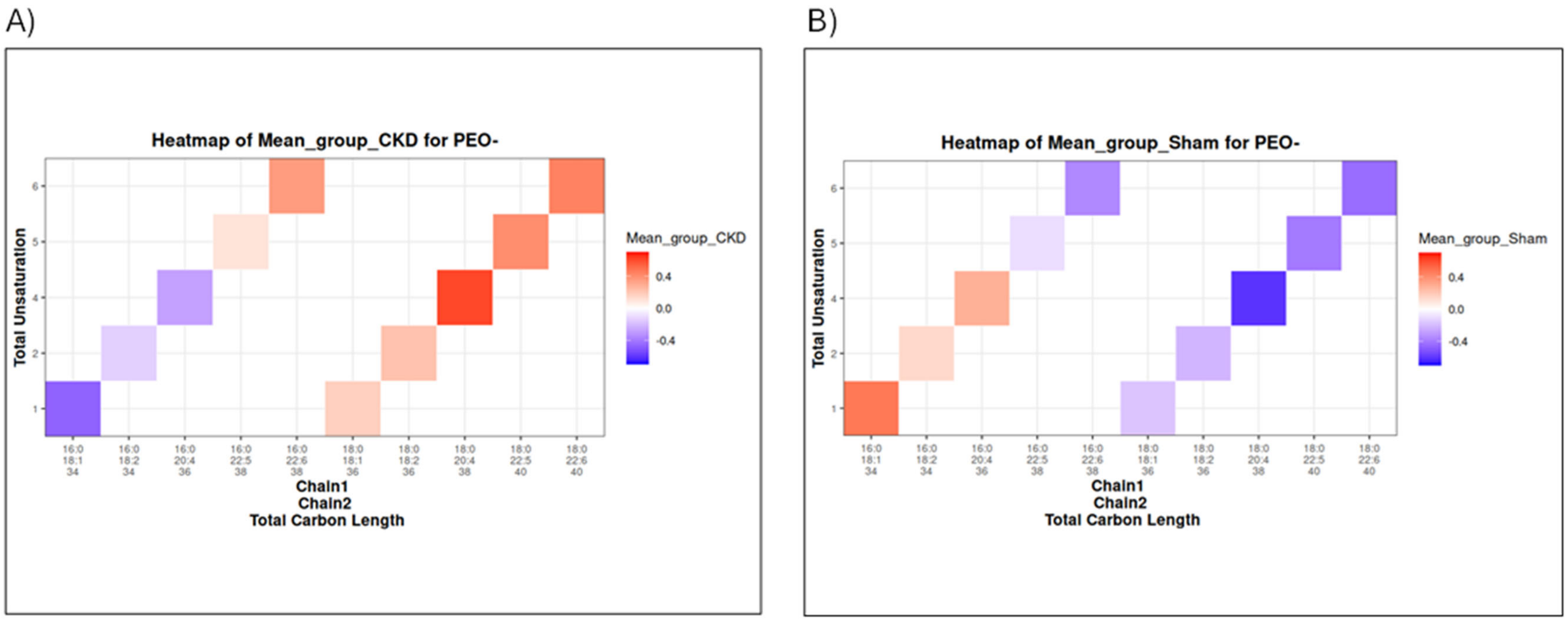

Figure S50: Sensitivity analysis of DMLH of phosphatidylethanolamine-O (PE-O) with lipid class median normalization, cube root transformation, and auto-scaling. Panel A represents the chronic kidney disease group. Panel B represents the sham group.

Troubleshooting guidance:

1- Lipidomic data file is not loading properly:
    a. Please ensure identifiers show up in column 1 when samples are shown in rows, as shown in the Example Lipidomics Data
2- Metadata is not loading properly:
    a. Please ensure sample names of metadata are consistent with the lipidomics data. To ensure proper format upload the Metadata Example file for review.
    b. Please ensure that the identifiers are properly listed in both lipidomics data file and the metadata.
3- Internal Standard data file is not loading properly:
    a. Please ensure identifiers for each sample correspond with that in the lipidomics and the metadata data files.
4- Lipid parsing: Lipid class, carbon numbers in lipid chains, and double bonds in acyl chain, and the total carbon number and number of double bonds do not align with prior knowledge about the lipid structure:
    a. Manually enter the correct carbon number, and number of double bonds for the correct chain and double bond position. The number of carbons and double bonds in lipid parsing are editable.
5- Correct Internal Standard is not selected or not assigned to proper lipid class for normalization:
    a. In the section of Internal Standard Selection, please select the proper internal standard for each lipid class to normalize by.
6- Graphs have low resolution:
    a. Adjust the "Output pixel dimension" in the corresponding page for optimal resolution.
7- DMLH may show overlapping numbers in X-axis:
    a. Adjust the font size in the corresponding or Y axes.

Downloading instructions from GitHub:

For users who wish to download the software for local use instead of working online, the following steps can be followed:

First, download the LipidAnalyst source code as a ZIP archive (or clone from the GitHub repository) and extract it. Open a terminal in the project directory, then build the Docker image and start the container using the commands below. Once the container is running, open http://localhost:3838/lipidanalyst/ in your web browser to access the application.

Run locally with Docker

1. Build image

```
docker build -t lipidanalyst:latest .
```

2. Run container

```
docker run --rm -p 3838:3838 lipidanalyst:latest
```

3. Open in browser

http://localhost:3838/lipidanalyst/
